# Combined Structural MR and Diffusion Tensor Imaging Classify the Presence of Alzheimer’s Disease With the Same Performance as MR Combined With Amyloid Positron Emission Tomography: A Data Integration Approach

**DOI:** 10.3389/fnins.2021.638175

**Published:** 2022-01-05

**Authors:** Daniel Agostinho, Francisco Caramelo, Ana Paula Moreira, Isabel Santana, Antero Abrunhosa, Miguel Castelo-Branco

**Affiliations:** ^1^Faculty of Medicine, Coimbra Institute for Biomedical Imaging and Translational Research (CIBIT), Institute for Nuclear Sciences Applied to Health (ICNAS), University of Coimbra, Coimbra, Portugal; ^2^Department of Neurology, Faculty of Medicine, Coimbra University Hospital (CHUC), University of Coimbra, Coimbra, Portugal

**Keywords:** Alzheimer’s disease (AD), multimodal classification, machine learning, ensemble learning, positron—emission tomography, MRI, DTI

## Abstract

**Background:** In recent years, classification frameworks using imaging data have shown that multimodal classification methods perform favorably over the use of a single imaging modality for the diagnosis of Alzheimer’s Disease. The currently used clinical approach often emphasizes the use of qualitative MRI and/or PET data for clinical diagnosis. Based on the hypothesis that classification of isolated imaging modalities is not predictive of their respective value in combined approaches, we investigate whether the combination of T1 Weighted MRI and diffusion tensor imaging (DTI) can yield an equivalent performance as the combination of quantitative structural MRI (sMRI) with amyloid-PET.

**Methods:** We parcellated the brain into regions of interest (ROI) following different anatomical labeling atlases. For each region of interest different metrics were extracted from the different imaging modalities (sMRI, PiB-PET, and DTI) to be used as features. Thereafter, the feature sets were reduced using an embedded-based feature selection method. The final reduced sets were then used as input in support vector machine (SVM) classifiers. Three different base classifiers were created, one for each imaging modality, and validated using internal (n = 41) and external data from the ADNI initiative (*n* = 330 for sMRI, *n* = 148 for DTI and *n* = 55 for PiB-PET) sources. Finally, the classifiers were ensembled using a weighted method in order to evaluate the performance of different combinations.

**Results:** For the base classifiers the following performance levels were found: sMRI-based classifier (accuracy, 92%; specificity, 97% and sensitivity, 87%), PiB-PET (accuracy, 91%; specificity, 89%; and sensitivity, 92%) and the lowest performance was attained with DTI (accuracy, 80%; specificity, 76%; and sensitivity, 82%). From the multimodal approaches, when integrating two modalities, the following results were observed: sMRI+PiB-PET (accuracy, 98%; specificity, 98%; and sensitivity, 99%), sMRI+DTI (accuracy, 97%; specificity, 99%; and sensitivity, 94%) and PiB-PET+DTI (accuracy, 91%; specificity, 90%; and sensitivity, 93%). Finally, the combination of all imaging modalities yielded an accuracy of 98%, specificity of 97% and sensitivity of 99%.

**Conclusion:** Although DTI in isolation shows relatively poor performance, when combined with structural MR, it showed a surprising classification performance which was comparable to MR combined with amyloid PET. These results are consistent with the notion that white matter changes are also important in Alzheimer’s Disease.

## Introduction

Alzheimer’s disease (AD), the most common form of dementia, is expected to affect 1 out of 85 people in the world in a near future, largely due to the increasing life expectancy (Brookmeyer et al., 2007; Dwyer, 2011). New therapeutic approaches are critical to mitigate its progression, as well as the implementation of biomarkers for early diagnosis (Paquerault, 2012; Wachinger and Reuter, 2016). Accordingly, the revised diagnostic criteria for Alzheimer’s disease (AD) emphasize the incorporation of neuroimaging biomarkers to support the diagnosis of AD (McKhann et al., 2011; Dubois et al., 2014). However, it remains unclear how the combination of imaging methods from a quantitative point of view can further contribute to imaging-based classification.

The use of neuroimaging biomarkers potentially provides sensitive and reliable measurement of AD progression than can help improve cognitive and clinical assessments (Ye et al., 2008; Green et al., 2012). Techniques such as magnetic resonance imaging (MRI) and positron emission tomography (PET) are routinely used in clinical cases for evaluating characteristic brain changes associated with AD (Bateman et al., 2012). However, in the clinical environment these biomarkers are generally used for subjective assessment using visual scales and sometimes complemented with isolated quantitative measurements extracted from the images, such as the hippocampal volume when accessing MRI (Scheltens et al., 2016).

Structural MRI (sMRI) can provide a non-invasive method that allows for the visualization, quantification and detection *in vivo* of structural alterations caused by AD. Volumetric measurements, from both the gray matter (GM) and white matter (WM), as well as cortical thickness estimations can be extracted and used reliably for the classification of AD (Frisoni et al., 2010; Beheshti and Demirel, 2015; Liu M. et al., 2015; Salvatore et al., 2015). Besides structural alterations, PET allows the visualization of functional and metabolic alterations *in vivo* through the use of different radiotracers. In AD studies, [18F]-Fluorodeoxyglucose (FDG) and [11C]-Pittsburgh Compound B (PiB) are used to extract measurements of glucose metabolism rates and the burden caused by the accumulation of abnormal Aβ protein, respectively (Nordberg et al., 2010; Leuzy et al., 2016; Oliveira et al., 2018).

Furthermore, the recent dissemination of the use of machine learning tools propelled the development of sophisticated, automatic, and objective classification frameworks capable of learning complex and subtle patterns of change across various imaging modalities without human subjectivity (Sajda, 2006). Using quantitative measurements extracted from neuroimaging modalities, it should be theoretically possible to construct a robust quantitative tool that offers a fast, systematic and standardized approach to aid the diagnosis of AD.

AD classification frameworks can be constructed using only a single imaging modality or through the combination of different modalities. The impact of the combination of modalities may be an important asset in the future, thus it deserves to be studied. Multimodal approaches have recently shown greater advantages over single image modalities, since different modalities can capture disease information from different perspectives, thereby improving the understanding of disease patterns over that presented by one modality (Zhang et al., 2011).

In Zhang et al. (2011), Liu L. et al. (2015) and Youssofzadeh et al. (2017), the combination of imaging data from the routinely used sMRI and PET achieved a higher classification accuracy. These results indicate that there is complementarity between the two imaging modalities. However, there are other imaging approaches that can be combined to improve the overall classification abilities.

Diffusion tensor imaging (DTI), an imaging modality of MRI, can be used to assess the integrity of cerebral WM fiber tracts and, hence, can potentially support the diagnosis of AD. DTI scalar measurements of anisotropic diffusion, such as fractional anisotropy (FA) and mean diffusivity (MD), can be extracted and used for classification (Dyrba et al., 2013, 2015; Maggipinto et al., 2017). Also in Dyrba et al. (2012) and Li et al. (2013), data from structural MRI and DTI were combined and, as seen before, DTI provided complementary information increasing the overall classification performance.

As seen in previous works (Zhang et al., 2011; Dyrba et al., 2012; Youssofzadeh et al., 2017), both PET and DTI provide complementary information to sMRI increasing the overall classification performance. However, there is no study where all these modalities are combined and all the possible effects analyzed.

In this paper, we explore this novel idea based on the unique combination of three imaging modalities, currently used in AD classification, and aim to explore the effects of all possible combinations between them. Furthermore, we want to evaluate if the combination of sMRI with DTI can achieve a comparable performance as sMRI combined with PET. These evaluations were performed by creating support vector machine (SVM) models for each modality (sMRI, DTI, PET) which were subsequently combined using a special ensemble technique.

## Materials and Methods

### Data Characteristics

In this paper, two different datasets were used. The internal dataset was obtained locally and divided into four different groups. Each group was constructed in the most balanced way possible and used for different objectives. The internal data were used to construct and initially validate the individual classifiers and is summarized in Table 1. All participants in the study generating the internal data gave their written informed consent, approved by the Ethics Committee of the University of Coimbra. The clinical group comprised individuals with early AD diagnosis (less than 2 years) recruited and prospectively evaluated by a neurologist (Head: IS), at the Memory Clinic of the Neurology Department of the Centro Hospitalar e Universitário de Coimbra (CHUC). The standard criteria for the diagnosis of AD were the Diagnostic and Statistical Manual of Mental Disorders—fourth edition (DSM-IV-TR) and the National Institute on Aging and the Alzheimer’s Association Workgroup (McKhann et al., 2011). They were in mild stages, according to the global staging scale Clinical Dementia Rating (CDR = 1). The control group was composed of age- and gender-matched individuals from the community, with no history of cognitive deterioration, neurological or acquired CNS disorders, traumatic brain injury, or psychiatric disorders. The control group was also submitted to a brief cognitive assessment to exclude the presence of cognitive impairment. Therefore, the individuals in the control group had no significant memory complaints (assessed by an SMC scale), a normal general cognitive function (assessed by MOCA), preserved daily living activities (assessed by Lawton and Brody scale) and no evidence of depressive symptoms (measured by Geriatric Depression Scale).

**TABLE 1:** Demographics and neuropsychologic characteristics for the study population.

	MRI		PiB-PET		DTI		Ensemble	
Condition	CN (*n* = 21)	AD (*n* = 20)	CN (*n* = 21)	AD (*n* = 17)	CN (*n* = 20)	AD (n = 17)	CN (n = 20)	AD (n = 15)
Age (years)	65.9 ± 6.8	66.3 ± 6.9	65.9 ± 6.8	66.4 ± 7.3	66.4 ± 6.5	65.8 ± 7.3	66.4 ± 6.5	65.6 ± 7.4
Gender (male/female)	10/11	10/10	10/11	8/9	10/10	9/8	10/10	8/7
MOCA	24.62 ± 4.44	14.35 ± 4.21	24.62 ± 4.44	14.18 ± 4.54	24.35 ± 4.38	14.41 ± 4.53	24.35 ± 4.38	14.41 ± 4.53
CDR	0.00 ± 0.00	1.00 ± 0.00	0.00 ± 0.00	1.00 ± 0.00	0.00 ± 0.00	1.00 ± 0.00	0.00 ± 0.00	1.00 ± 0.00

*CN, Cognitively Normal; AD, Alzheimer’s Disease; Age, MOCA, and CDR values are defined as mean ± standard deviation.*

Furthermore, the external data obtained from ADNI database were organized into three groups of data that were constructed aiming to externally validate the classifiers constructed using the internal data and is summarized in Table 2.

**TABLE 2:** Demographics and neuropsychologic characteristics for the ADNI external data.

	External MRI		External PiB-PET		External DTI	
Condition	CN (*n* = 164)	AD (*n* = 166)	CN (*n* = 31)	AD (*n* = 24)	CN (*n* = 71)	AD (*n* = 77)
Age (years)	76.5 ± 5.9	75.1 ± 7.9	79.5 ± 5.8	74.4 ± 8.0	72.7 ± 7.2	73.7 ± 8.4
Gender (male/female)	77/87	83/83	19/12	18/6	28/43	40/37
MMSE	29.17 ± 1.09	22.93 ± 2.23	29.00 ± 1.46	24.17 ± 1.83	29.13 ± 1.13	23.32 ± 1.88
CDR	0.01 ± 0.08	0.83 ± 0.36	0.00 ± 0.00	0.85 ± 0.35	0.00 ± 0.00	1.07 ± 0.39

*CN, Cognitively Normal; AD, Alzheimer’s Disease; Age, MMSE, and CDR values are defined as mean ± standard deviation.*

### Imaging Data Acquisition

#### [11C]-Pittsburgh Compound B (PiB) PET (PiB-PET)

A Philips Gemini GXL PET/CT scanner (Philips Medical Systems, Best, **T**he Netherlands) was used to perform a dynamic 3-dimensional PET [11C]-PiB scan of the entire brain (90 slices, 2-mm slice sampling) and a low-dose brain CT scan, for attenuation correction. PET scan started immediately after the intravenous bolus injection of approximately 555 MBq of [11C]-PiB and was acquired over a period of 90 min (37 frames: 4 × 15 s + 8 × 30 s + 9 × 60 s + 2 × 180 s + 14 × 300 s). To minimize head movement, the patients’ head was restrained with a soft elastic tape. PET data were reconstructed using a LOR-RAMLA algorithm (Sato et al., 2008), with attenuation and scatter correction.

#### Structural Magnetic Resonance Imaging

T1 MPRAGE anatomic acquisitions were performed with the following imaging parameters: repetition time (TR)/echo time (TE)/inversion time (TI)/flip angle = 2,530 ms/3.42 ms/1,100 ms/7°; a FOV (field of view) of 256×256 mm with a matrix size of 256×256; 176 sagittal slices were performed with voxel resolution 1.0×1.0×3.0 mm^3^; total time of acquisition (TA) = 6 min and 3 s.

#### Diffusion Tensor Imaging

Diffusion-tensor imaging (DTI) had TR/TE/number of excitations (NEX) = 7,800 ms/90 ms/1; matrix, 96 × 96 × 63 contiguous axial slices; isotropic voxel resolution of 2 × 2 × 2 mm^3^; bandwidth of 1,628 Hz/pixel and echo spacing of 0.72 ms. The diffusion tensor was acquired along 63 non-collinear directions (*b* = 1,000s/mm^2^), with one scan without diffusion weighting (*b* = 0 s/mm^2^, b0). Generalized Autocalibrating Partially Parallel Acquisitions (GRAPPA) was used to reduce the scanning time to around 9 min.

#### Data for External Validation

An external dataset was acquired in order to perform external validation. This external dataset was obtained from the three phases of the Alzheimer’s Disease Neuroimaging Initiative (ADNI) database (ADNI1, ADNI2 and ADNI3)^1^ (see sample size in Table 1). The primary goal of ADNI has been to test whether serial magnetic resonance imaging (MRI), positron emission tomography (PET), other biological markers, and clinical and neuropsychological assessment can be combined to measure the progression of mild cognitive impairment (MCI) and early Alzheimer’s disease (AD). For more information regarding the used external data (see Weiner et al., 2013, 2017).

### Data Processing

The first common step between all the image modalities was converting all of the images from DICOM format to NIFTI format. Afterward, standard preprocessing was applied, before further data processing.

Before any processing was done, T1 Weighted MR brain images were manually aligned, so that origin of the image was the anterior commissure (AC). This step is important since the used processing tools assume that the origin of the image is there.

After the alignment, T1 Weighted MR brain images were processed using the Computational Anatomy Toolbox version 12 (CAT12),^2^ for Statistical Parametric Mapping 12 (SPM12),^3^ in the MatLab environment.^4^ The images were processed using the segment data option of the toolbox, and features were generated using region- or label-based morphometry (RBM), given by the CAT12 toolbox. RBM is a predefined atlas-based analysis that allows the estimation of regional tissue volumes, as well as cortical thickness values from different volumes or surface-based atlas maps (Magnin et al., 2009; Rathore et al., 2017).

PiB-PET images were preprocessed using SPM12. Firstly, the sum image that reflects the total accumulation was calculated and then coregistered to the corresponded T1 Weighted image. This allows the application of the same spatial normalization transformation from the T1 Weighted image to the PiB-PET image, providing a more accurate spatial normalization. The sum image was spatially normalized to the T1 MRI template ICBM152, given by SPM12. The normalized images were then visually inspected in order to verify the existence of obvious imperfections. Lastly, the normalized images were smoothed, using SPM12 smoothing and a Gaussian smoothing kernel, with full width at half maximum (FWHM) of 8 mm. The preprocessed PET-PIB images were then analyzed using a similar approach to the T1 Weighted images through regions of interest. The Standard Uptake Value (SUV) for nineteen regions of interest, plus three reference regions were extracted considering the mean value of intensity for each region extracted. The SUV of each region was then normalized to the dose injected and the body mass index (BMI). Three different sets of features, containing the same nineteen regions of interest, were then considered and constructed using the Standard Uptake Value Ratio (SUVR). The SUVR was computed for all the nineteen regions of interest, using three different reference regions: Cerebellum, WM and GM, resulting in three different datasets (SUVR_*Cerebellum*_, SURV_*WM*_ and SUVR_*GM*_), each one containing the nineteen regions of interest that were normalized for a different reference region. All of the regions were defined on the T1 MRI template ICBM152 (Oliveira et al., 2018).

From Diffusion Weighted images (DWIs), the Diffusion Tensor Images (DTI) were constructed using ExploreDTI.^5^ DTI were then corrected for head motion, eddy currents and EPI distortions, with deformation axes set to [1 0 0] and image type set to FA. After the correction, the DTI data were spatially normalized to FA atlas template SRI24,^6^ or to FA atlas template IIT Human Brain Atlas,^7^ or to FA atlas template ICBM.^8^ The use of different atlases for normalization to a common space for the DTI data was necessary in order to be able to perform region-label analysis since the label atlases used for this analysis were constructed in these different spaces. DTI features were generated following a similar structure to T1 Weighted MR images, but instead of generating volumetric or surface values, in this case, diffusion metrics were extracted from different label atlas: lpba40 (see text footnote 6), Desikan, Destrieux (see text footnote 6), Hammers and JHU (see text footnote 8). Using the ExploreDTI software,^9^ the diffusion metrics were generated from the different atlases (Dyrba et al., 2015).

Only the mean fractional anisotropy (FA) and mean diffusivity (MD) of the various atlas regions were considered as features.

### Feature Selection

Due to the relatively small sample size (*n* = 40) of the internal data set and the vast number of features generated, feature selection methods were applied in order to select the best feature subset for each imaging modality. The subset was limited to 8 features, 1 feature for each 5 cases to be selected. The total amount of features for each model can be consulted in Supplementary Table S1.

There are a great variety of methods that can be used for supervised feature selection. These can be broadly organized into three categories known as filter, wrapper, and embedded methods (Kumar, 2014; Tang et al., 2014; Jović et al., 2015). Taking this in consideration, we decided to apply an embedded-based method (EBM) and a filter-based method (FBM) to reduce the number of features. This was done in order to achieve a final set of features that could be considered unbiased of the feature selection method utilized. Both feature selection methods were implemented in R environment (R Core Team, 2020). In the EBM, we started by randomly dividing the data into two groups, assuring that both groups were balanced in order to simulate the final environment that will be used to train the classifiers. Following this, subsets of 8 features were randomly created from all the features and used to construct a random forest model (Liaw and Wiener, 2002). Using random forest allowed us to extract the relative importance given by the learning algorithm to each feature. The previous process was performed 2,000 times in each run and the importance of each feature was stored. The final importance value for each individual feature was established as the mean importance value of each feature. For the FBM, the data was divided into two groups, one containing the AD positive cases and the other containing the cognitively normal. Posteriorly, student *t*-test was performed for each feature comparing the two groups and the respective *t*- and *p*-values for each feature were extracted.

After extracting the final importance value in the EBM, the features were sorted from the biggest to the smallest importance values and a filter was applied to remove all of the features that exhibited an importance value lesser than 0.55 times the most important feature. This was done to prevent the use of possible irrelevant features. For FBM, the features were sorted in the same way but this time in relation to their *t*-value and those that showed a *p*-value greater than 0.05 were removed (Supplementary Data Sheet S1). Furthermore, a refined filter was applied, using the Pearson correlation (r). This step was common to both methods.

**FIGURE 1:**
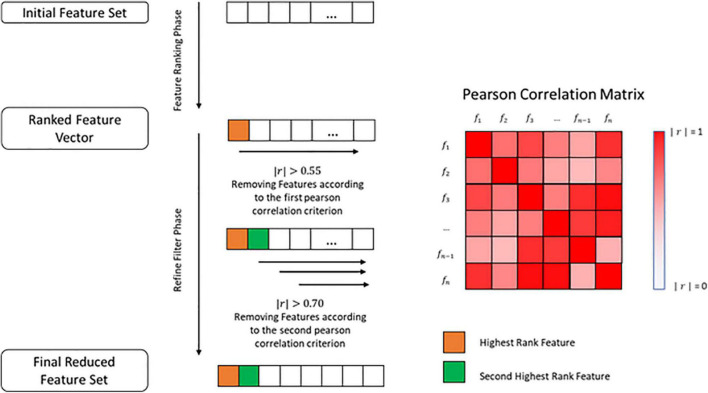
Illustration of the feature selection method used.

We used Pearson correlation to construct a n-by-n matrix that represented the correlation values between all features. In this matrix, each row represents a feature, and the columns represent the Pearson correlation between the feature and all the other features. Using this correlation matrix, we removed all the features that had an absolute correlation value greater than 0.55 (|*r*|>0.55) in relation to the most important feature, placed at the first row of the correlation matrix. Furthermore, the resultant features set were evaluated from the second row until the n-1 row. The cut-off criteria, in this case, were an absolute correlation value greater than 0.70 (|*r*| > 0.70), which means that those that exceeded that value were excluded (Figure 1 and Supplementary Data Sheet S1).

In the end, the remaining feature vectors from both methods were reduced to contain only the best 8 features as delimited in advance by the relatively small size of the data.

### Classification, External Validation and Ensemble

The construction and evaluation of the classifiers was performed in a Python environment (Van Rossum and Drake, 2009). Before any learning algorithm was applied, all feature vectors were standardized to zero mean and variance one. This was achieved by subtracting the mean to each feature vector, and then dividing it by the standard deviation (Equation 1). This was meant to improve the performance of the learning algorithm.


(1) $$x^{{}^{\prime }}=\frac{x-\bar{x}}{s}$$

The now standardized data were split into two groups, 80% used for training and 20% for testing. The splitting was performed in such way that both groups were as balanced as possible.

**FIGURE 2:**
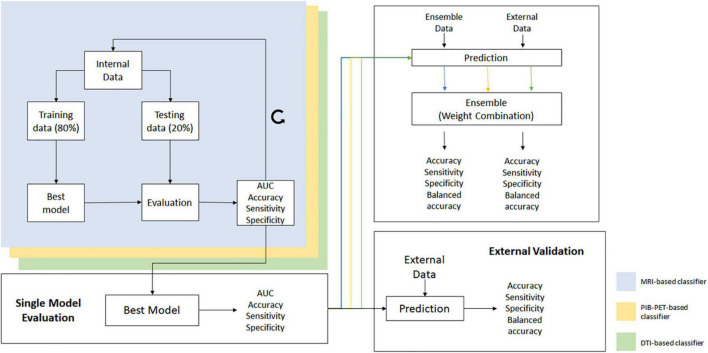
Overall scheme of all processes used for the construction and validation of the individual models and ensemble.

The training group was evaluated, using random permutation of the data, and at each cycle the data was shuffled and divided into training and testing data, assuring that the division created was balanced in training and testing groups. At the end of each cycle, the values of accuracy, sensitivity, specificity and the ROC curve and AUC were stored. This process was repeated 2,000 times, and the classifiers overall performance was primarily evaluated using the mean ROC curve and the mean AUC (Figure 2) (Supplementary Data Sheet S1).

Furthermore, parameters such as mean accuracy, sensitivity and specificity were used in order to distinguish the classifiers that have similar performance measurements, as well as to select those that will be used in the ensemble phase.

The learning algorithm used was support vector machine (SVM) which was constructed using the radial basis function (RBF) kernel, with gamma value set to “scale” and C value set to the default of 0.1, using the scikit-learning package (Pedregosa et al., 2011).

For the construction of the individual classifiers, different feature sets were extracted from different label atlases. For each feature set, a model was constructed using the aforementioned method. The most promising models, for each imaging modality, were then evaluated using the external data (thus preventing overfitting) and the ones with the best performance selected to be used in the ensemble phase.

The final selected classifiers were used to evaluate the internal ensemble data (Table 1) and the potential of combining all image modalities was assessed.

To ensemble the classifiers, a non-generative weighted fusion technique was used, and the previously constructed and validated classifiers were combined in order to generate a final decision. The base classifiers were combined using a weight value between 0 and 1, that was applied to the predicted probability, given from each of the base learning algorithm.


(2) Yi=c1⁢y1i+c2⁢y2i+c3⁢y3i

In Equation 2, *Y_i_* stands for the final ensemble prediction probability, y1i for the predicted probability, given by the classifier trained using a MRI-based model, y2i for the predicted probability, given by the classifier trained using a PiB-based model and y3i for the predicted probability, given by the classifier trained using a DTI-based model. Lastly, c_1_, c_2_, and c_3_ are weights applied to the different modalities, so that c_1_ + c_2_ + c_3_ = 1.

In order to evaluate the overall performance of the ensemble method, the individual classifiers, one for each modality, were first used alone to evaluate the ensemble data and to establish a base line performance. Afterward, the prediction of each classifier was combined using Equation (2) and the performance parameters of accuracy, sensitivity and specificity for the ensemble technique were stored (Figure 2 and Supplementary Data Sheet S1). Furthermore, the mean ROC curves from the individual classifiers and the ensemble classifier were also calculated.

The ensemble technique was further validated using the external data, but unfortunately, only the validation through the combination of MRI and DTI models was possible from the external data collected.

## Results

### Feature Selection

The results of the feature selection methods for all the models evaluated can be found in Supplementary Tabel S1. In Table 3, we summarize the initial number of features, as well as the final number of features that survived the different feature selection methods. Furthermore, in Table 4, we present the final set of regions that are used for extracting the features used for the construction of the selected individual classifiers (Table 5) for each of the evaluated imaging modalities.

**TABLE 3:** Summary of the feature selection methods for the selected models.

Model	Feature selection method	Number of total features	Number of surviving features	Number of the final set of features
MRI-based	EBM	142	41	8
	FBM		10	8
PiB-PET-based	EBM	19	2	2
	FBM		3	3
DTI-based	EBM	84	58	8
	FBM		6	6

**TABLE 4:** List of the regions used for extracting features that will be used in the construction of the individual classifiers.

MRI-based	PiB-PET-based	DTI-based
Left amygdala	Prefrontal cortex	Left inferior temporal gyrus
Right inferior temporal gyrus	Right anterior putamen	Left pericalcarine
Left thalamus proper		Left entorhinal
Left middle occipital gyrus		Right thalamus
Left putamen		Right insula
Left central operculum		Left amygdala
Right posterior cingulate gyrus		Left pars triangularis
Right superior frontal gyrus		Right temporal pole

**TABLE 5:** Classifier’s performance using embedded-based feature selection for all atlases.

Imaging modality	Atlas	ACC	SEN	SPEC	AUC
MRI structural	Cobra GM	76.13%	76.06%	76.19%	0.87 ± 0.13
	Cobra WM	78.43%	79.04%	77.04%	0.88 ± 0.12
	Hammers GM	74.79%	66.14%	83.44%	0.85 ± 0.15
	Hammers WM	58.36%	58.24%	58.49%	0.51 ± 0.24
	Hammers CSF	75.37%	77.01%	73.73%	0.85 ± 0.14
	Lpba40 GM	70.28%	61.16%	79.40%	0.76 ± 0.20
	**Neuromorphometrics GM**	**92.05%**	**86.78%**	**97.32%**	**0.96 ± 0.04**
	Neuromorphometrics CSF	76.13%	80.34%	71.91%	0.84 ± 0.15
MRI surface	a2009 Gyrification	83.01%	86.98%	79.04%	0.91 ± 0.10
	a2009 Thickness	81.89%	81.93%	81.87%	0.90 ± 0.11
	Dk40 Gyrification	69.13%	73.14%	65.11%	0.75 ± 0.19
	Dk40 Thickness	72.19%	71.01%	73.36%	0.79 ± 0.17
	HCP Gyrification	83.11%	73.65%	92.56%	0.94 ± 0.08
	HCP Thickness	84.27%	83.05%	85.49%	0.93 ± 0.09
DTI	Lpba40 FA	71.34%	65.37%	75.81%	0.72 ± 0.24
	Lpba40 MD	75.29%	67.32%	81.04%	0.86 ± 0.15
	**Desikan FA**	**79.84%**	**76.67%**	**82.23%**	**0.86 ± 0.15**
	Desikan MD	62.00%	52.70%	68.98%	0.66 ± 0.24
	Destrieux FA	65.66%	63.23%	67.49%	0.66 ± 0.25
	Destrieux MD	77.60%	72.15%	81.69%	0.84 ± 0.15
	Hammers FA	76.77%	63.80%	86.50%	0.79 ± 0.19
	Hammers MD	69.52%	55.63%	79.94%	0.72 ± 0.22
	JHU FA	77.24%	67.15%	84.81%	0.80 ± 0.19
	JHU MD	63.86%	47.47%	76.16%	0.61 ± 0.26
PiB-PET	SUVR Cerebellum	87.71%	95.92%	81.26%	0.94 ± 0.09
	SUVR GM	91.98%	98.66%	87.01%	0.97 ± 0.06
	**SUVR WM**	**90.68%**	**92.78%**	**89.10%**	**0.93 ± 0.10**

*ACC, Accuracy; SEN, Sensitivity; SPEC, Specificity. Bold values indicates the base models that were selected to use in the ensemble phase.*

Furthermore, the correlation between the selected features (Table 4) and the age and cognitive scores (MOCA) of the participants was also evaluated and can be found in Supplementary Figrue S1.

### Individual Classifiers

For each imaging modality, different sets of features were initially evaluated. The features were extracted from different label atlases and their performance evaluated on the internal data. In Table 5, we summarize the results from the classifier’s performance using a single modality and using the EBM feature selection method for the RBF kernel. Each model from the Table 5 was only constructed using features from only one label atlases and is named after the used label atlas and the used feature selection method. Furthermore, Supplementary Table S2 shows the results for the models found in Table 5 using of a SVM model built using a linear kernel and Supplementary Table S3 shows the results for the same models using the FBM feature selection method.

Furthermore, by testing the most promising models in the internal data with the external data (Table 2), we were able to select the most promising model, i.e., the one that showed the best performance in both internal and external data, in order to represent each imaging modality in the ensemble phase. After the individual evaluation of all the models, those which were selected were the Neuromorphometrics-GM EBM model, for sMRI, the SUVR-WM EBM model, for PiB-PET and the Desikan-FA EBM model for DTI.

In Table 6, we summarize the performance of the selected models on both the internal and external data.

**TABLE 6:** Best performing models validated on both the internal and external data.

		Internal data					External data				
Imaging modality	Model name	AUC	ACC	SEN	SPEC	BACC	AUC	ACC	SEN	SPEC	BACC
MRI	Neuromorphometrics GM EBM	0.96 ± 0.07	92.05%	86.78%	86.78%	92.05%	0.81 ± 0.02	78.02%	74.12%	82.29%	78.20%
PiB-PET	SUVR WM EBM	0.93 ± 0.10	90.53%	92.00%	89.43%	90.53%	0.81 ± 0.04	76.87%	87.90%	68.33%	78.12%
DTI	Desikan FA EBM	0.86 ± 0.14	76.84%	76.17%	82.09%	79.84%	0.69 ± 0.04	62.79%	54.31%	71.98%	63.15%

*ACC, Accuracy; SEN, Sensitivity; SPEC, Specificity; BACC, Balanced Accuracy.*

### Ensemble Classification

Four different combinations of data integration were tested. Three multimodal approaches combining two different imaging modalities, using a weight value of 1/2 for each modality involved, and one novel approach combining three imaging modalities using a weight of 1/3 for all modalities. Using the models from Table 6 to evaluate the ensemble data group (Table 1), we obtained the following performances for the base classifiers, MRI-based (AUC, 0.99 ± 0.01; Accuracy, 95.04%; Sensitivity, 90.04%; Specificity, 99.04%; Balanced Accuracy, 95.04%), PiB-PET-based (AUC, 0.92 ± 0.02; Accuracy, 88.75%; Sensitivity, 88.23%; Specificity, 89.17%; Balanced Accuracy, 88.75%) and DTI-based (AUC, 0.97 ± 0.01; Accuracy, 92.55%; Sensitivity, 90.58%; Specificity, 94.13%; Balanced Accuracy, 92.55%).

**FIGURE 3:**
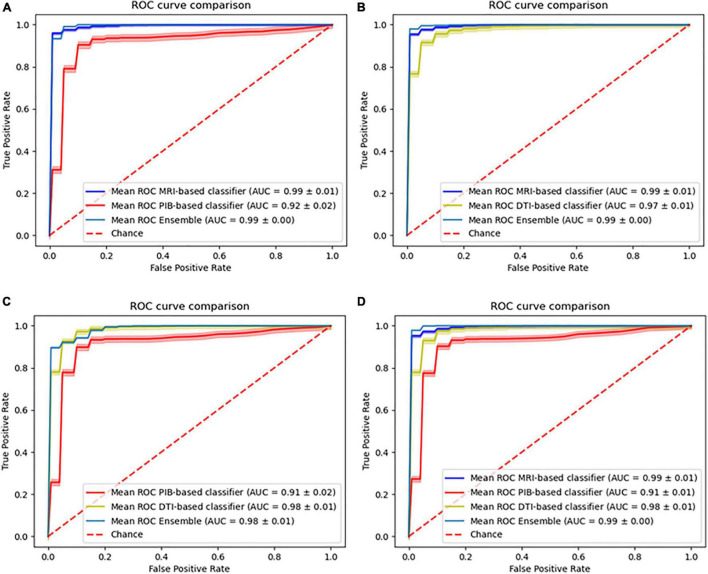
Comparison of the ROC curves of the individual models and the ensemble models. **(A)** Combination of MRI with PiB-PET, **(B)** Combination of MRI with DTI, **(C)** Combination of PiB-PET with DTI, **(D)** Combination of all.

For the ensemble classifiers performances, we obtained for the different combinations MRI+PiB-PET (AUC, 0.99 ± 0.00; Accuracy, 98.05%; Sensitivity, 98.59%; Specificity, 97.62%; Balanced Accuracy, 98.05%), MRI+DTI (AUC, 0.99 ± 0.00; Accuracy, 97.30%; Sensitivity, 94.62%; Specificity, 99.43%; Balanced Accuracy, 97.30%), PiB-PET+DTI (AUC, 0.98 ± 0.01; Accuracy, 91.28%; Sensitivity, 92.61%; Specificity, 90.02%; Balanced Accuracy, 91.28%) and for all (AUC, 0.99 ± 0.00; Accuracy, 98.11%; Sensitivity, 99.14%; Specificity, 97.27%; Balanced Accuracy, 98.11%) (Figure 3).

**FIGURE 4:**
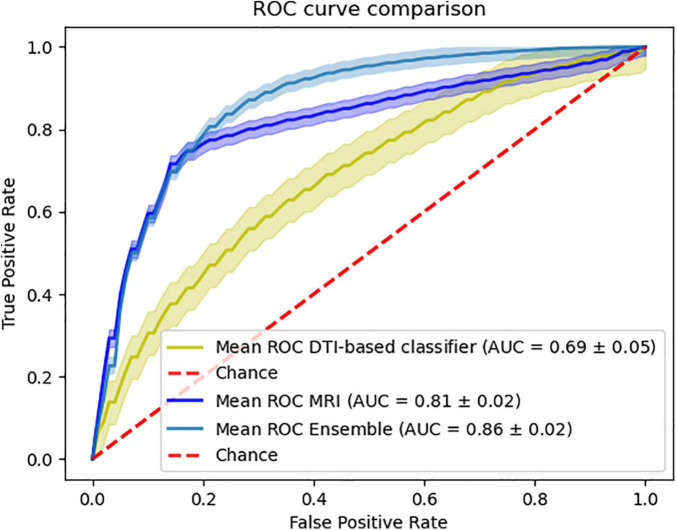
Ensemble results of combining MRI and DTI data. Comparison of the ROC curve of the classifiers using the external data.

Furthermore, we were able to test our ensemble method on the external data combining the sMRI and DTI-based classifiers. This combination yields an accuracy of 78.59%, sensitivity of 77.15% specificity of 80.16% and a balanced accuracy of 78.66% (Figure 4).

## Discussion

In this paper, we have proposed to evaluate the different effects regarding all the possible combinations of different imaging modalities used for AD classification.

The results obtained for the base, single modality classifiers’ performance (Table 5) were in accordance with the literature (Dyrba et al., 2012, 2013; Beheshti and Demirel, 2015; Möller et al., 2016; Rathore et al., 2017; Oliveira et al., 2018). In particular, it is possible to observe that the individual classifiers constructed using sMRI or PiB-PET data can achieve a relatively high-performance values by themselves, in contrast with those using DTI data, which alone do not yield the same performance. Furthermore, from the Table 4 and Supplementary Table S3 is possible to see that the selection of the label atlas used for feature extraction can have a significant impact in the performance of the models. This finding was also observed in other AD related studies (Ota et al., 2015) suggesting that different label atlas allow the capture of different information within the same image modality and between imaging modalities.

Regarding the selected classifiers (Table 6), it is also important to point out that the final anatomical features, that remained after feature selection (Table 4), generally showed a significant correlation with cognitive scores as measured with MOCA and they also correspond to regions where biologically significance has been ascribed in the context of Alzheimer’s Disease, thereby providing clinical meaning to our results (Frisoni et al., 2010; Nordberg et al., 2010).

The apparent discrepancy between the performance values from the base classifiers (Table 5 and Supplementary Table S3) and the individual performances in the ensemble phase (Table 6) can be attributed to the lower number of cases being evaluated (Table 1) and the training-test procedure. However, we did not expect that the DTI-base classifier would outperform the PiB-PET-based classifier.

Also, there is the concern that our data normalization approach may cause data leakage which is a limitation in this study and may be inflating the performance of our single modality classifiers. However, in our classification approach (Figure 2) we took measures to mitigate this issue, mainly using random permutation and cross validation. Furthermore, we went beyond this and search for an external dataset, containing data very different from our internal dataset and used it to validate our model, which showed that in fact our models were not overfitted to the internal data.

Regarding the multi-model approaches, there are several studies that evaluate the effects of combining information from different imaging modalities or other physiological data on the classification of AD. The results that we obtained from the combination of sMRI with PiB-PET (accuracy = 98.05%) and sMRI with DTI (accuracy = 97.30%) are consistent with other studies that evaluated the same combination. Regarding the sMRI combination with PiB-PET, Youssofzadeh et al. (2017) obtained an accuracy value of 95.70% and Liu L. et al. (2015) achieved an accuracy value around 90%. Regarding the sMRI combination with DTI, Dyrba et al. (2012), obtained an accuracy of 89.2% and Dyrba et al. (2015) achieved an accuracy of 85.00%.

As expected from the previous studies found in the literature, these combinations showed a performance improvement when compared to single modality models. These findings bolster the idea that both PiB-PET and DTI convey independent information that complements that of the sMRI data.

Also, we were surprised to see that the combination of sMRI with DTI shows an improvement in the overall classification performance that can be comparable to the improvement caused by the combination of sMRI with PiB-PET. This surprising finding can be further emphasized by the fact that, in spite of the isolated low performance of DTI, the combination of MRI with DTI when using only external data also provides a significant improvement in performance (Figure 4). Furthermore, the analysis of Figure 4 showed that the combination of the sMRI and the DTI model, built using the internal data, shows the same behavior when analyzing the samples from the external data. Unfortunately, the nature of external data renders unfeasible to perform this same analysis for other combinations.

Furthermore, we evaluated two novel combinations combining PiB-PET with DTI and also combining all three modalities. The results of combining PiB-PET with DTI showed that this combination does not yield an improvement of performance for the overall classifier. This new information leads to the suggestion that both PiB-PET and DTI are not complementary because they may be reflecting non-independent biological processes. This finding suggests that, although in practice multi-modality approaches show better results than single modality approaches (Cabral and Silveira, 2013; Gupta et al., 2019), one should carefully select the type of data as a function of its biological significance and the corresponding acquisition protocols in order to achieve a synergic effect on the classification. In particular our results suggests that even if a single modality is not very useful in isolation it can provide strong added value in combination, in particular if it provides independent biological information.

Finally, the results of combining all the modalities do not yield an improvement in relation to the combination of MRI with PiB-PET or DTI, emphasizing the fact that perhaps DTI and PiB-PET contribute with the same (redundant) information for the classification problem. Such redundancy might be explained by the notion that changes in white matter might be present in PIB images (Oliveira et al., 2018) which are probably correlated with the changes detected using DTI.

These last two findings may suggest some underlying unknown biological factor involving the WM in the pathophysiology of the disease, which is related to amyloid pathology.

## Conclusion

The goal of this work was to evaluate the effects of combining three imaging modalities that are vastly used in AD classification problems. The results of our ensemble models reached the surprising conclusion that, in fact, the addition of DTI data to the sMRI can provide a classification performance that can be as good as the addition of PiB-PET data.

Furthermore, by analyzing the results from the combination of PiB-PET with DTI, we can see that there is no complementarity between the two data sources, suggesting redundancy. This can indicate that both imaging modalities are communicating non-independent information in the case of AD classification.

This last finding should be further explored, since it also indicates that there is some implicit unknown alteration on the WM, related to amyloid pathology, that can be further investigated and used not only for the development of new AD classification frameworks but to improve our understanding of disease pathophysiology at the level of the WM.

Also, these results suggest that, in the future, it could be possible to base the diagnosis of AD in only sMRI and DTI imaging data, potentially without the need of using PiB-PET images. This could bring some benefits for the patient, who will not be submitted to radiation and subjected to a lower discomfort from lengthy acquisition protocols of PiB-PET (∼90 min) in comparison to those of MRI (∼6 min) and DTI (∼9 min), which is relevant in old participants with cognitive impairment. Furthermore, the use of DTI presents a less expensive approach since it does not require the acquisition or production, as well as maintenance of radioactive isotopes.

Finally, the use of MRI and DTI images only requires the use of one machine. Nevertheless, and in spite of these practical advantages, the use of PiB-PET images is still a powerful and useful tool for the study of the disease’s pathology.

## Data Availability Statement

The raw data supporting the conclusions of this article will be made available by the authors, without undue reservation.

## Ethics Statement

The studies involving human participants were reviewed and approved by Faculdade de Medicina da Universidade de Coimbra. The patients/participants provided their written informed consent to participate in this study.

## Author Contributions

DA, MC-B, and FC: conceptualization, analysis, and writing. AM, AA, and IS: analysis and writing. All authors contributed to the article and approved the submitted version.

## Conflict of Interest

The authors declare that the research was conducted in the absence of any commercial or financial relationships that could be construed as a potential conflict of interest..

## Publisher’s Note

All claims expressed in this article are solely those of the authors and do not necessarily represent those of their affiliated organizations, or those of the publisher, the editors and the reviewers. Any product that may be evaluated in this article, or claim that may be made by its manufacturer, is not guaranteed or endorsed by the publisher.

## Footnotes

1
adni.loni.usc.edu

2
www.neuro.uni-jena.de/cat/index.html

3
www.fil.ion.ucl.ac.uk/spm/software/spm12/

4
www.mathworks.com

5
www.exploredti.com

6
www.nitrc.org/frs/?group_id=214

7
www.nitrc.org/frs/?group_id=432

8
identifiers.org/neurovault.collection:264

9
www.exploredti.com/
