# Supplementary material for: Combined Structural MR and Diffusion Tensor Imaging Classify the Presence of Alzheimer’s Disease With the Same Performance as MR Combined With Amyloid Positron Emission Tomography: A Data Integration Approach
Source: Front Neurosci. 2022 Jan 5;15:638175. doi: 10.3389/fnins.2021.638175 (PMC8766722; doi:10.3389/fnins.2021.638175)
Supplement: Supplementary file 1 [file Data_Sheet_1.docx]

**Algorithm1.** Feature selection method (Feature ranking)

**m** = number of participants

**n** = number of features

**Data** = the full data set matrix (**m** x **n**)

**I** = matrix for storing the values of importance for each feature for all runs (2000 x **n**). (EBM)

**SoF** = set containing k subsets of 8 features constructed from the total number of features. (EBM)

**FtV** = Features t value (FBM)

**FpV** =Features p value (FBM)

**if** EBM

**repeat** 2000 times:

**Training Data** = Balance Split with random permutation (**Data**) only 80% of the total data

**repeat for** s in **SoF**:

**Model** = RandomForest (Training data [, s])

Store the values of importance for the features in **s in I**

**FI** = mean feature importance of **I** (vector 1 x **n**)**.**

**sort** the **FI** vector

Remove importance values that were less than 0.55 times the most important feature (**FI[0]**)

**if** FBM

**repeat** for all n features:

Perform the student t test and store the value of t and p for all features in **FtV** and **FpV** respectively**.**

Remove features with p value greater than 0.05.

**sort** the **FpV** vector

**Algorithm 2.** Feature selection (Refine filter)

**FV** = ranked feature vector

**P** = Matrix containing the pearson correlation between the features in **FV**

**Function** First filter(**feature vector**):

**rvector** = vector containing the r values for the first feature P[0, ]

**repeat** for the length of the **feature vector** times:

**if** **rvector[i]** greater than 0.55 remove feature i.

update **P** removing the features removed

**Function** Second filter(**feature vector**):

**repeat** for the n-1 feature in **feature vector**:

**rvector** = vector containing the r values for the in feature **P**[i, ]

**repeat** from the next index feature until the n feature in **feature vector**:

if **P**[k, ] is greater than 0.7 remove feature k

update **P**

**final feature set** = Second filter (First filter (**FV**))

**Algorithm 3.** Classification

**data** = input data

**repeat** 2000 times:

**training data, testing data** = Balance splitting with random permutation of **data** (80% training 20% testing)

**best model** = 8-fold-cross validation (**training data**)

use the **best model** to predict the **testing data** and store the performance metrics (accuracy, sensibility, sensitivity, and AUC).

**Algorithm 4.** Ensemble

**data** = dataset containing all the participants with complementary imaging data from all modalities.

**k** = number of imaging modalities (MRI, PET, and DTI)

**imaging modalities weights** = weights for each imaging modality to be used in the weight fusion part of the ensemble.

**function** weight ensemble (**models, weights, testing data**):

**models predictions** = list storing the predictions for each model

**repeat for** **model** in **models:**

**model prediction = model.predict_probability (testing data)**

append model prediction to **models predictions.**

**final prediction =** weight sum of the predictions in **models predictions** using **weights** and threshold of 0.5.

**repeat** 100 times**:**

**training data**, **testing data** = Balance splitting with random permutation of **data** (80% training 20% testing)

**models =** store the models one for each modality

**repeat** for all imaging modalities in k:

**new** **model =** 8-fold-cross validation (**training data**)

add the **new** **model** to **models**

**ensemble prediction** = **weight ensemble (models, imaging modalities weights, testing data)**

compare the ensemble prediction to the true labels and store the performance metrics (accuracy, sensibility, sensitivity, and AUC)
