# Supplementary material for: Combined Structural MR and Diffusion Tensor Imaging Classify the Presence of Alzheimer’s Disease With the Same Performance as MR Combined With Amyloid Positron Emission Tomography: A Data Integration Approach
Source: Front Neurosci. 2022 Jan 5;15:638175. doi: 10.3389/fnins.2021.638175 (PMC8766722; doi:10.3389/fnins.2021.638175)
Supplement: Supplementary file 2 [file Image_1.pdf]

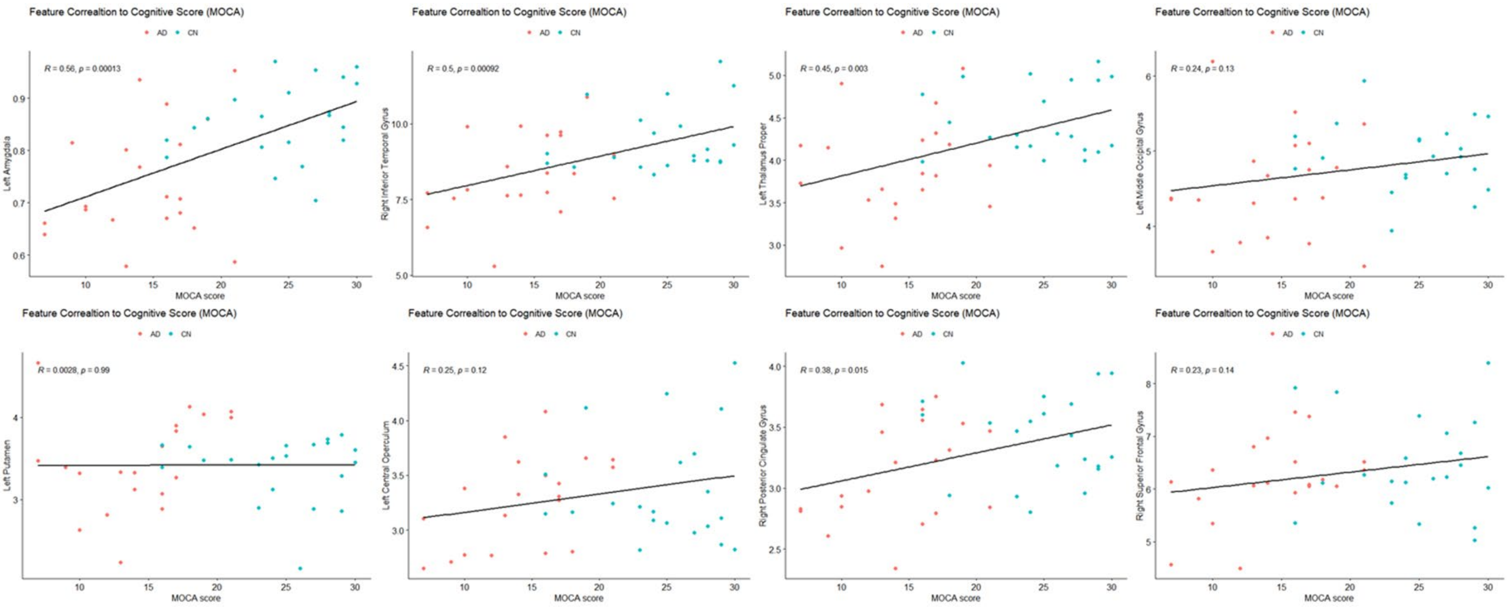

Figure S2.1. Correlation between selected features from the MRI-based model and the cognitive MOCA score

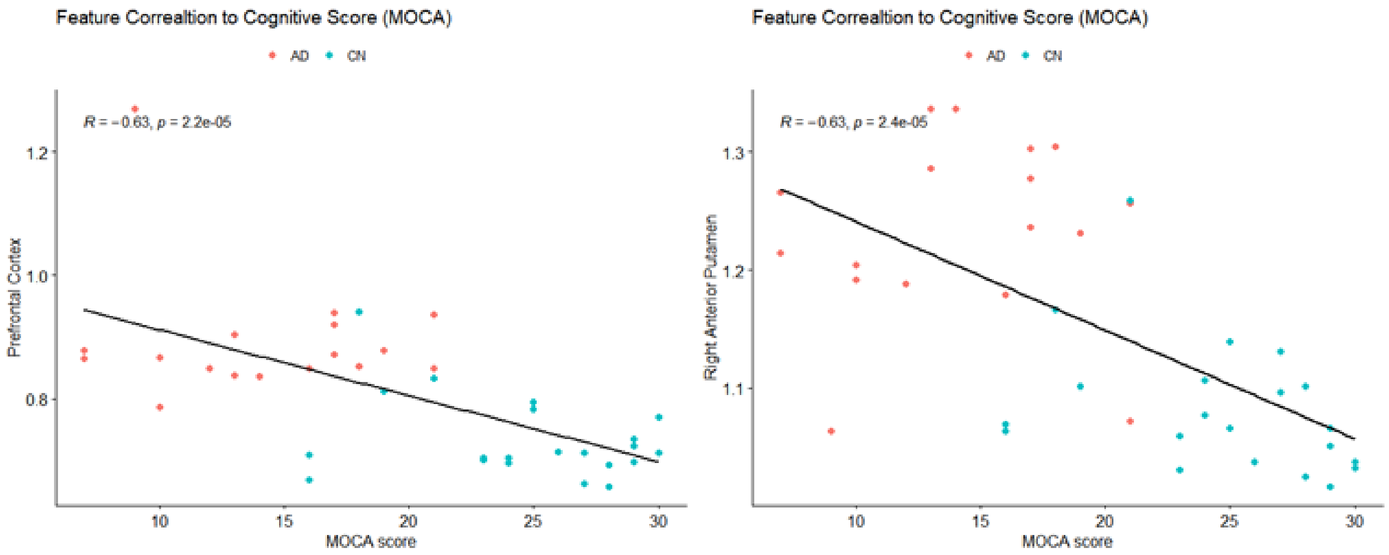

Figure S2.2 Correlation between selected features from the PiB-PET-based model and the cognitive MOCA score.

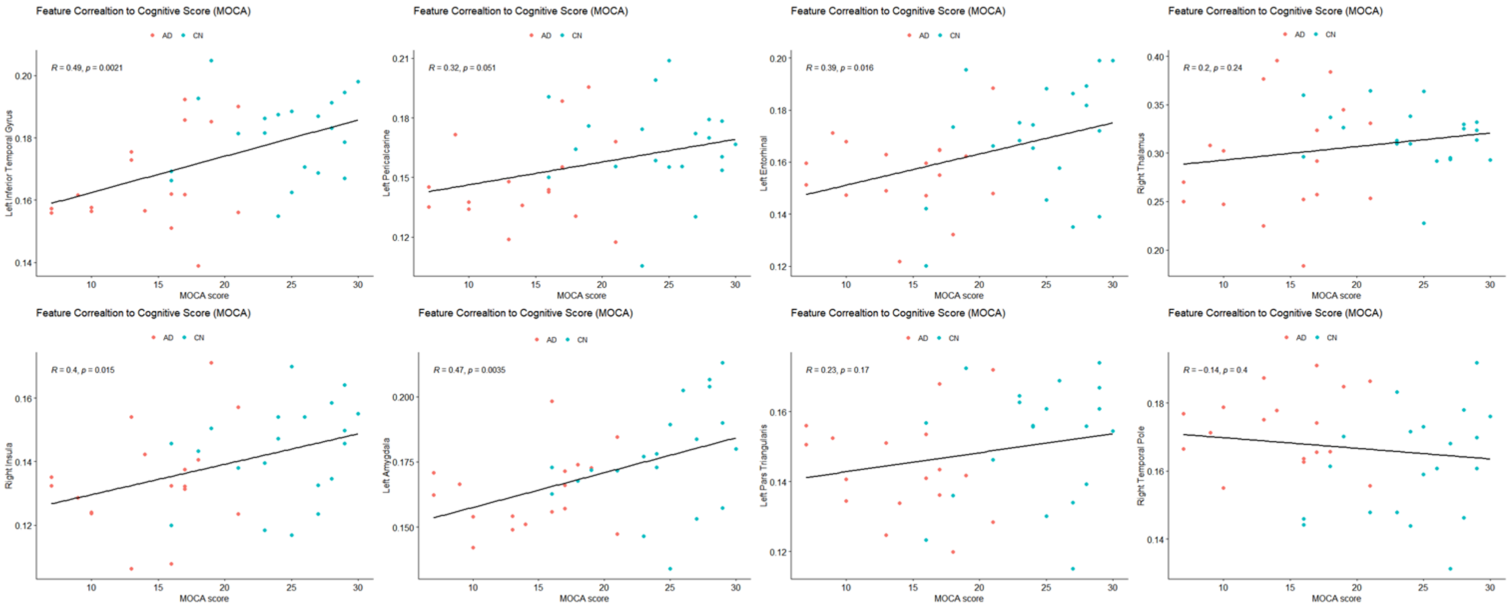

Figure S2.3 Correlation between selected features from the DTI-based model and the cognitive MOCA score.

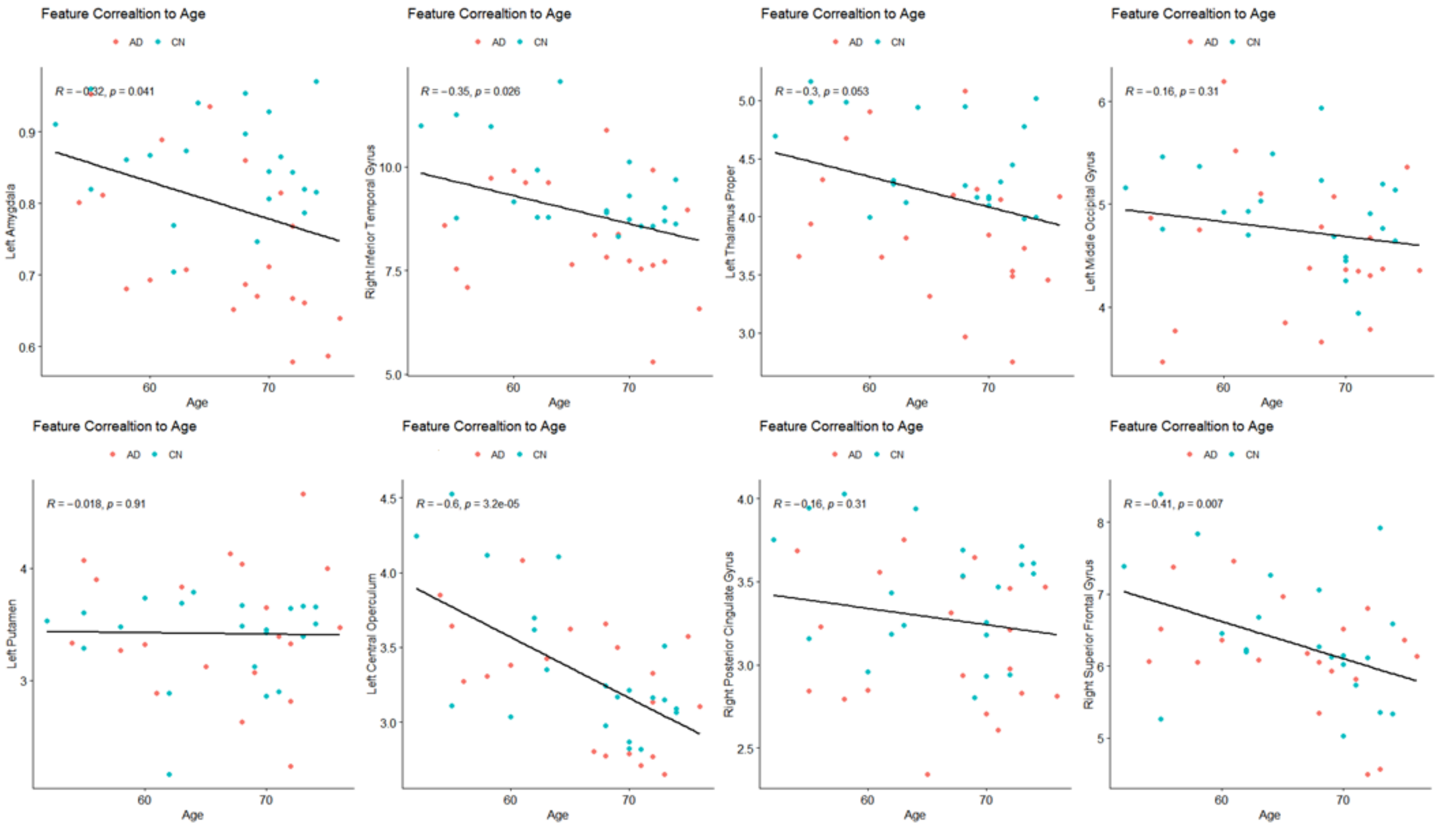

Figure S2.4 Correlation between selected features from the MRI-based model and the participant's age.

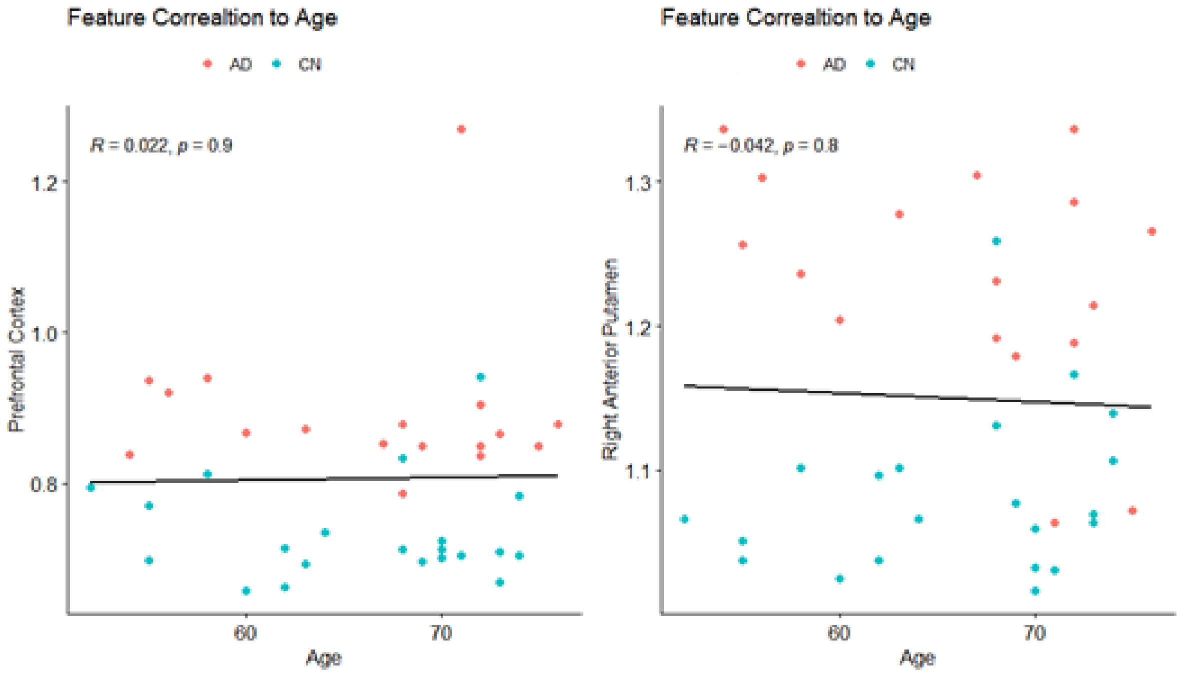

Figure S2.5 Correlation between selected features from the PiB-PET-based model and the participant's age.

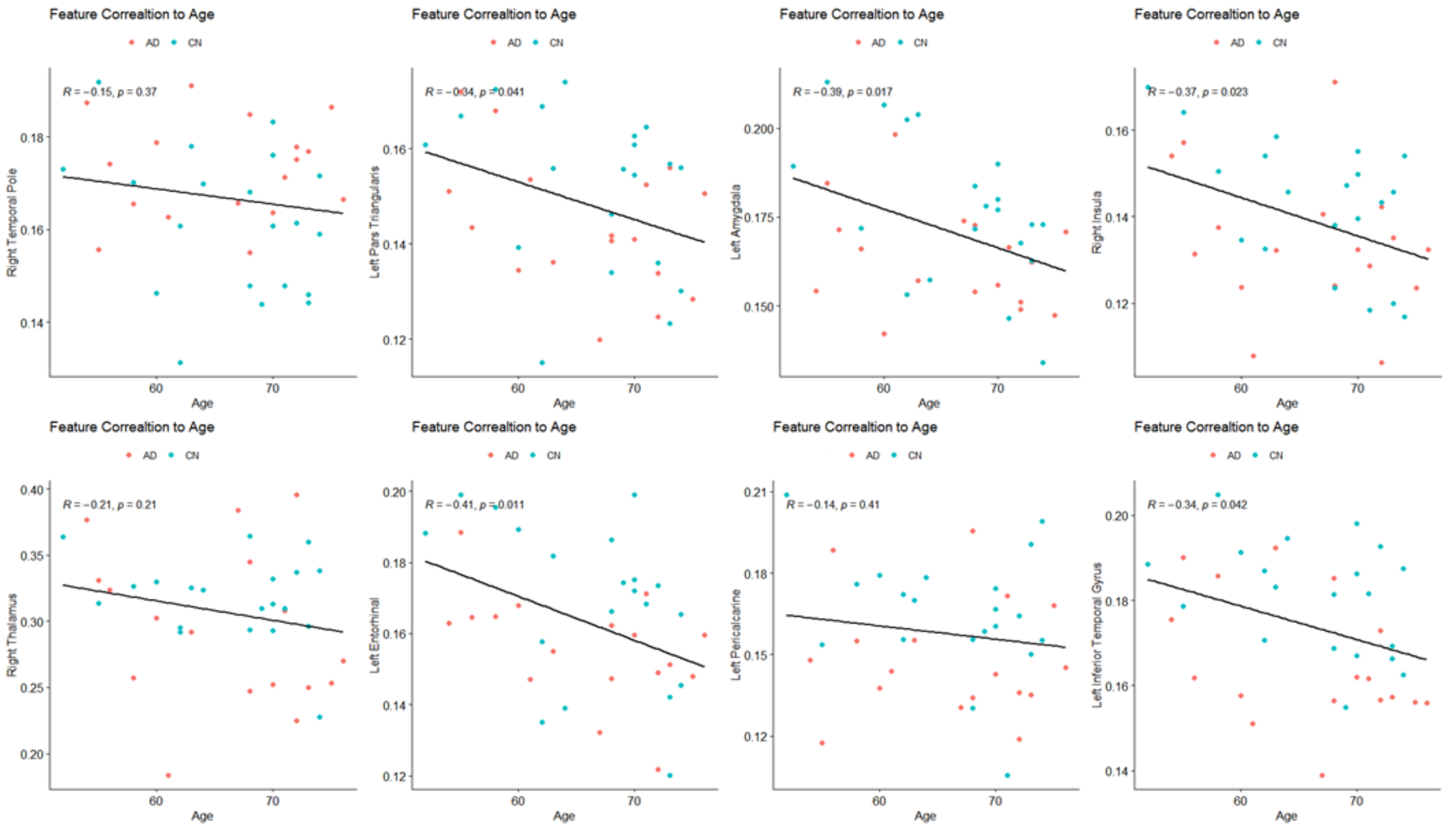

Figure S2.6 Correlation between selected features from the DTI-based model and the participant's age.
