# Supplementary material for: Combined Structural MR and Diffusion Tensor Imaging Classify the Presence of Alzheimer’s Disease With the Same Performance as MR Combined With Amyloid Positron Emission Tomography: A Data Integration Approach
Source: Front Neurosci. 2022 Jan 5;15:638175. doi: 10.3389/fnins.2021.638175 (PMC8766722; doi:10.3389/fnins.2021.638175)
Supplement: Supplementary file 3 [file Table_1.DOCX]

**S1.1. Ranked features for both the EBM and FBM feature selection methods for the Cobra GM model.**

| **EBM** | **FBM** |
| --- | --- |
| Left Amygdala | Left Amygdala |
| Right Alveus | Right CA4/Dentate Gyrus |
| Right CA4/Dentate Gyrus | Right Hippocampus CA1 |
| Right Hippocampus CA1 | Right Striatum |
| Right Striatum | Right Alveus |
| Left CA4/Dentate Gyrus | Left Hippocampus CA1 |
| Right Amygdala | Left Subiculum |
| Left Alveus | Left Alveus |
| Left Thalamus | Left Thalamus |
| Left Hippocampus CA1 | Left CA4/Dentate Gyrus |
| Left Subiculum | Right Amygdala |
| Left Stratum Radiatum/Lacunosum/Moleculare | Right Thalamus |
| Left Fimbria | Right CA2/CA3 |
| Right Thalamus | Left Stratum Radiatum/Lacunosum/Moleculare |
| Right CA2/CA3 | Left Fimbria |
| Right Fimbria | Right Fimbria |
| Left CA2/CA3 | Left CA2/CA3 |
| Left Mammillary body | Right Subiculum |
| Right Inferior Posterior Cerebellar Lobule X | Left Mammillary body |
| Left Anterior Cerebellar Lobule III | Left Fornix |
| Left Superior Posterior Cerebellar Lobule VIIB | Right Inferior Posterior Cerebellar Lobule X |
| Right Subiculum | Left Superior Posterior Cerebellar Lobule VI |
| Left Cerebellar White Matter | Left Anterior Cerebellar Lobule V |
| Right Inferior Posterior Cerebellar Lobule VIIIA | Right Fornix |
| Left Anterior Cerebellar Lobule V | Left Cerebellar White Matter |
| Right Striatum | Right Striatum |
| Left Striatum | Left Superior Posterior Cerebellar Lobule VIIB |
| Right Anterior Cerebellar Lobule III | Left Inferior Posterior Cerebellar Lobule VIIIA |
| Left Fornix | Right Anterior Cerebellar Lobule V |
| Left Superior Posterior Cerebellar Lobule Crus II | Left Anterior Cerebellar Lobule III |
| Right Superior Posterior Cerebellar Lobule VIIB | Left Striatum |
| Right Superior Posterior Cerebellar Lobule Crus II | Right Anterior Cerebellar Lobule I-II |
| Left Superior Posterior Cerebellar Lobule VI | Left Inferior Posterior Cerebellar Lobule X |
| Left Inferior Posterior Cerebellar Lobule VIIIA | Right Inferior Posterior Cerebellar Lobule VIIIA |
| Right Anterior Cerebellar Lobule V | Left Anterior Cerebellar Lobule I-II |
| Right Globus Pallidus | Right Globus Pallidus |
| Right Superior Posterior Cerebellar Lobule VI | Left Inferior Posterior Cerebellar Lobule IX |
| Right Inferior Posterior Cerebellar Lobule IX | Left Superior Posterior Cerebellar Lobule Crus I |
| Left Anterior Cerebellar Lobule IV | Left Superior Posterior Cerebellar Lobule Crus II |
| Right Anterior Cerebellar Lobule IV | Right Superior Posterior Cerebellar Lobule Crus II |
| Left Globus Pallidus | Right Mammillary body |
| Right Fornix | Right Anterior Cerebellar Lobule III |
| Left Anterior Cerebellar Lobule I-II | Right Superior Posterior Cerebellar Lobule Crus I |
| Right Mammillary body | Right Inferior Posterior Cerebellar Lobule IX |
| Left Inferior Posterior Cerebellar Lobule X | Right Anterior Cerebellar Lobule IV |
| Right Cerebellar White Matter | Left Globus Pallidus |
| Right Superior Posterior Cerebellar Lobule Crus I | Right Superior Posterior Cerebellar Lobule VIIB |
| Right Inferior Posterior Cerebellar Lobule VIIIB | Left Inferior Posterior Cerebellar Lobule VIIIB |
| Left Superior Posterior Cerebellar Lobule Crus I | Left Anterior Cerebellar Lobule IV |
| Left Inferior Posterior Cerebellar Lobule IX | Right Cerebellar White Matter |
| Left Inferior Posterior Cerebellar Lobule VIIIB | Right Inferior Posterior Cerebellar Lobule VIIIB |
| Right Anterior Cerebellar Lobule I-II | Right Superior Posterior Cerebellar Lobule VI |

**S1.2. Ranked features for both the EBM and FBM feature selection methods for the Cobra WM model.**

| **EBM** | **FBM** |
| --- | --- |
| Right Anterior Cerebellar Lobule III | Right Anterior Cerebellar Lobule III |
| Right Inferior Posterior Cerebellar Lobule X | Left Mammillary body |
| Left Mammillary body | Left Globus Pallidus |
| Left Globus Pallidus | Right Globus Pallidus |
| Left Inferior Posterior Cerebellar Lobule X | Left Thalamus |
| Left Fimbria | Right CA2/CA3 |
| Left Striatum | Right Stratum Radiatum/Lacunosum/Moleculare |
| Left Thalamus | Left Subiculum |
| Right Subiculum | Right Thalamus |
| Right Fornix | Right Fornix |
| Right Stratum Radiatum/Lacunosum/Moleculare | Left Superior Posterior Cerebellar Lobule VI |
| Right CA4/Dentate Gyrus | Left Striatum |
| Right Anterior Cerebellar Lobule IV | Right Subiculum |
| Right Globus Pallidus | Left Fimbria |
| Left Anterior Cerebellar Lobule III | Left Anterior Cerebellar Lobule III |
| Left Stratum Radiatum/Lacunosum/Moleculare | Left Inferior Posterior Cerebellar Lobule X |
| Right CA2/CA3 | Left Stratum Radiatum/Lacunosum/Moleculare |
| Right Striatum | Right Striatum |
| Right Thalamus | Left Fornix |
| Left Anterior Cerebellar Lobule V | Right Inferior Posterior Cerebellar Lobule X |
| Left Subiculum | Left CA2/CA3 |
| Left Superior Posterior Cerebellar Lobule VI | Right Superior Posterior Cerebellar Lobule VI |
| Right Cerebellar White Matter | Left Anterior Cerebellar Lobule V |
| Right Inferior Posterior Cerebellar Lobule VIIIA | Left Inferior Posterior Cerebellar Lobule VIIIB |
| Left Inferior Posterior Cerebellar Lobule IX | Right Anterior Cerebellar Lobule V |
| Right Superior Posterior Cerebellar Lobule VIIB | Right Mammillary body |
| Left Cerebellar White Matter | Left Superior Posterior Cerebellar Lobule Crus I |
| Right Anterior Cerebellar Lobule V | Right CA4/Dentate Gyrus |
| Left Hippocampus CA1 | Left Inferior Posterior Cerebellar Lobule IX |
| Right Anterior Cerebellar Lobule I-II | Right Superior Posterior Cerebellar Lobule Crus I |
| Left Inferior Posterior Cerebellar Lobule VIIIB | Right Anterior Cerebellar Lobule IV |
| Left Fornix | Left Hippocampus CA1 |
| Right Superior Posterior Cerebellar Lobule VI | Right Hippocampus CA1 |
| Right Superior Posterior Cerebellar Lobule Crus I | Right Fimbria |
| Left Superior Posterior Cerebellar Lobule VIIB | Left Amygdala |
| Right Inferior Posterior Cerebellar Lobule IX | Right Cerebellar White Matter |
| Right Fimbria | Left Anterior Cerebellar Lobule IV |
| Right Alveus | Left Inferior Posterior Cerebellar Lobule VIIIA |
| Left Anterior Cerebellar Lobule IV | Right Inferior Posterior Cerebellar Lobule IX |
| Left Superior Posterior Cerebellar Lobule Crus I | Left Cerebellar White Matter |
| Right Mammillary body | Right Superior Posterior Cerebellar Lobule Crus II |
| Left Amygdala | Right Superior Posterior Cerebellar Lobule VIIB |
| Right Hippocampus CA1 | Right Anterior Cerebellar Lobule I-II |
| Left Superior Posterior Cerebellar Lobule Crus II | Right Amygdala |
| Right Inferior Posterior Cerebellar Lobule VIIIB | Right Alveus |
| Right Superior Posterior Cerebellar Lobule Crus II | Right Inferior Posterior Cerebellar Lobule VIIIA |
| Right Amygdala | Left Superior Posterior Cerebellar Lobule Crus II |
| Left Alveus | Left CA4/Dentate Gyrus |
| Left CA4/Dentate Gyrus | Left Alveus |
| Left Inferior Posterior Cerebellar Lobule VIIIA | Left Superior Posterior Cerebellar Lobule VIIB |
| Left CA2/CA3 | Right Inferior Posterior Cerebellar Lobule VIIIB |
| Left Anterior Cerebellar Lobule I-II | Left Anterior Cerebellar Lobule I-II |

**S1.3 Ranked features for both the EBM and FBM feature selection methods for the Hammers GM model.**

| **EBM** | **FBM** |
| --- | --- |
| Left Amygdala | Right Hippocampus |
| Left Thalamus | Left Fusiform Gyrus |
| Right Hippocampus | Left Amygdala |
| Left Fusiform Gyrus | Right Fusiform Gyrus |
| Right Posterior Temporal Lobe | Left Thalamus |
| Right Fusiform Gyrus | Left Anterior Medial Temporal Lobe |
| Left Posterior Temporal Lobe | Right Thalamus |
| Left Ambient and Parahippocampus Gyri | Left Hippocampus |
| Left Anterior Medial Temporal Lobe | Left Ambient and Parahippocampus Gyri |
| Left Hippocampus | Left Posterior Temporal Lobe |
| Right Amygdala | Right Amygdala |
| Right Thalamus | Left Inferior Middle Temporal Gyri |
| Right Anterior Medial Temporal Lobe | Right Anterior Medial Temporal Lobe |
| Left Inferior Lateral Pariatal Lobe | Left Anterior Lateral Temporal Lobe |
| Right Inferior Middle Temporal Gyri | Right Corpus Callosum |
| Left Anterior Lateral Temporal Lobe | Right Posterior Cinguli Gyrus |
| Right Inferior Lateral Pariatal Lobe | Right Inferior Middle Temporal Gyri |
| Right Posterior Cinguli Gyrus | Left Corpus Callosum |
| Left Accumbens Nucleus | Left Inferior Lateral Pariatal Lobe |
| Left Middle Frontal Gyrus | Right Posterior Temporal Lobe |
| Left Putamen | Left Third Ventricle |
| Right Lingual Gyrus | Left Anterior Cinguli Gyrus |
| Right Anterior Lateral Temporal Lobe | Right Third Ventricle |
| Left Inferior Middle Temporal Gyri | Left Superior Temporal Gyrus |
| Right Superior Temporal Gyrus | Right Anterior Lateral Temporal Lobe |
| Right Anterior Cinguli Gyrus | Left Posterior Cinguli Gyrus |
| Left Superior Parietal Gyrus | Left Superior Parietal Gyrus |
| Left Superior Temporal Gyrus | Right Inferior Lateral Pariatal Lobe |
| Right Corpus Callosum | Left Middle Frontal Gyrus |
| Right Caudate Nucleus | Right Superior Parietal Gyrus |
| Left Corpus Callosum | Right Ambient and Parahippocampus Gyri |
| Right Superior Parietal Gyrus | Left Accumbens Nucleus |
| Right Third Ventricle | Right Anterior Cinguli Gyrus |
| Left Posterior Cinguli Gyrus | Left Lateral Temporal Ventricle |
| Right Lateral Occipital Lobe | Right Insula |
| Right Superior Frontal Gyrus | Right Superior Temporal Gyrus |
| Left Lateral Occipital Lobe | Right Superior Frontal Gyrus |
| Left Orbito-Frontal Gyri | Right Inferior Frontal Gyrus |
| Right Precentral Gyrus | Left Superior Frontal Gyrus |
| Right Insula | Right Middle Frontal Gyrus |
| Right Cerebellum | Right Caudate Nucleus |
| Left Cerebellum | Left Insula |
| Left Lingual Gyrus | Left Orbito-Frontal Gyri |
| Right Ambient and Parahippocampus Gyri | Left Cuneus |
| Left Third Ventricle | Left Lateral Occipital Lobe |
| Right Lateral Temporal Ventricle | Left Gyrus Rectus |
| Left Lateral Temporal Ventricle | Right Lateral Occipital Lobe |
| Left Anterior Cinguli Gyrus | Right Lateral Temporal Ventricle |
| Right Putamen | Left Caudate Nucleus |
| Left Precentral Gyrus | Right Pallidum |
| Right Postcentral Gyrus | Right Postcentral Gyrus |
| Left Caudate Nucleus | Right Putamen |
| Right Orbito-Frontal Gyri | Right Accumbens Nucleus |
| Left Inferior Frontal Gyrus | Right Gyrus Rectus |
| Right Middle Frontal Gyrus | Left Inferior Frontal Gyrus |
| Right Gyrus Rectus | Left Lingual Gyrus |
| Left Superior Frontal Gyrus | Right Cerebellum |
| Left Postcentral Gyrus | Right Cuneus |
| Right Cuneus | Right Brainstem |
| Left Insula | Left Brainstem |
| Right Inferior Frontal Gyrus | Left Putamen |
| Right Accumbens Nucleus | Left Cerebellum |
| Left Gyrus Rectus | Right Precentral Gyrus |
| Left Cuneus | Right Orbito-Frontal Gyri |
| Right Brainstem | Left Postcentral Gyrus |
| Left Brainstem | Right Lingual Gyrus |
| Right Pallidum | Left Precentral Gyrus |
| Left Pallidum | Left Pallidum |

**S1.4 Ranked features for both the EBM and FBM feature selection methods for the Hammers WM model.**

| **EBM** | **FBM** |
| --- | --- |
| Left Thalamus | Left Caudate Nucleus |
| Left Caudate Nucleus | Right Caudate Nucleus |
| Left Brainstem | Left Thalamus |
| Right Pallidum | Right Pallidum |
| Right Caudate Nucleus | Right Thalamus |
| Right Insula | Left Pallidum |
| Right Brainstem | Left Brainstem |
| Right Thalamus | Left Putamen |
| Left Ambient and Parahippocampus Gyri | Right Insula |
| Left Putamen | Left Insula |
| Left Pallidum | Right Brainstem |
| Left Insula | Left Inferior Frontal Gyrus |
| Left Superior Frontal Gyrus | Left Third Ventricle |
| Left Cuneus | Left Lateral Temporal Ventricle |
| Left Middle Frontal Gyrus | Right Orbito-Frontal Gyri |
| Left Amygdala | Left Inferior Middle Temporal Gyri |
| Right Fusiform Gyrus | Left Accumbens Nucleus |
| Left Hippocampus | Left Ambient and Parahippocampus Gyri |
| Left Lateral Temporal Ventricle | Left Amygdala |
| Right Anterior Lateral Temporal Lobe | Right Lateral Temporal Ventricle |
| Right Putamen | Right Fusiform Gyrus |
| Left Posterior Cinguli Gyrus | Right Hippocampus |
| Right Amygdala | Right Accumbens Nucleus |
| Left Posterior Temporal Lobe | Right Lingual Gyrus |
| Right Accumbens Nucleus | Left Precentral Gyrus |
| Left Accumbens Nucleus | Right Precentral Gyrus |
| Left Inferior Middle Temporal Gyri | Right Putamen |
| Right Middle Frontal Gyrus | Left Postcentral Gyrus |
| Right Anterior Medial Temporal Lobe | Right Cuneus |
| Left Superior Temporal Gyrus | Left Superior Parietal Gyrus |
| Left Postcentral Gyrus | Left Posterior Temporal Lobe |
| Left Fusiform Gyrus | Left Orbito-Frontal Gyri |
| Right Anterior Cinguli Gyrus | Right Inferior Middle Temporal Gyri |
| Right Precentral Gyrus | Right Superior Parietal Gyrus |
| Right Hippocampus | Left Anterior Medial Temporal Lobe |
| Right Postcentral Gyrus | Left Lingual Gyrus |
| Right Orbito-Frontal Gyri | Right Ambient and Parahippocampus Gyri |
| Right Inferior Middle Temporal Gyri | Right Postcentral Gyrus |
| Right Posterior Temporal Lobe | Left Cerebellum |
| Right Superior Parietal Gyrus | Left Posterior Cinguli Gyrus |
| Right Corpus Callosum | Right Corpus Callosum |
| Left Anterior Lateral Temporal Lobe | Left Fusiform Gyrus |
| Left Orbito-Frontal Gyri | Left Gyrus Rectus |
| Left Precentral Gyrus | Right Anterior Lateral Temporal Lobe |
| Right Cuneus | Left Anterior Lateral Temporal Lobe |
| Right Lateral Temporal Ventricle | Right Amygdala |
| Right Inferior Frontal Gyrus | Right Third Ventricle |
| Right Superior Frontal Gyrus | Left Corpus Callosum |
| Right Posterior Cinguli Gyrus | Right Anterior Cinguli Gyrus |
| Right Gyrus Rectus | Left Middle Frontal Gyrus |
| Right Ambient and Parahippocampus Gyri | Right Superior Frontal Gyrus |
| Right Inferior Lateral Pariatal Lobe | Right Inferior Frontal Gyrus |
| Left Anterior Cinguli Gyrus | Right Cerebellum |
| Left Inferior Frontal Gyrus | Right Inferior Lateral Pariatal Lobe |
| Left Superior Parietal Gyrus | Left Inferior Lateral Pariatal Lobe |
| Left Anterior Medial Temporal Lobe | Right Gyrus Rectus |
| Left Gyrus Rectus | Left Cuneus |
| Right Lingual Gyrus | Left Hippocampus |
| Left Cerebellum | Right Posterior Cinguli Gyrus |
| Right Superior Temporal Gyrus | Right Middle Frontal Gyrus |
| Left Third Ventricle | Left Superior Frontal Gyrus |
| Left Lateral Occipital Lobe | Right Superior Temporal Gyrus |
| Right Lateral Occipital Lobe | Right Posterior Temporal Lobe |
| Left Inferior Lateral Pariatal Lobe | Right Anterior Medial Temporal Lobe |
| Left Lingual Gyrus | Left Superior Temporal Gyrus |
| Right Third Ventricle | Right Lateral Occipital Lobe |
| Left Corpus Callosum | Left Anterior Cinguli Gyrus |
| Right Cerebellum | Left Lateral Occipital Lobe |

**S1.5 Ranked features for both the EBM and FBM feature selection methods for the Hammers CSF model.**

| **EBM** | **FBM** |
| --- | --- |
| Left Inferior Frontal Gyrus | Left Inferior Frontal Gyrus |
| Left Insula | Left Insula |
| Left Anterior Lateral Temporal Lobe | Left Anterior Lateral Temporal Lobe |
| Right Hippocampus | Left Gyrus Rectus |
| Left Superior Temporal Gyrus | Left Superior Temporal Gyrus |
| Right Superior Temporal Gyrus | Left Amygdala |
| Left Orbito-Frontal Gyri | Right Anterior Medial Temporal Lobe |
| Left Hippocampus | Left Anterior Cinguli Gyrus |
| Right Posterior Temporal Lobe | Left Ambient and Parahippocampus Gyri |
| Right Orbito-Frontal Gyri | Left Orbito-Frontal Gyri |
| Right Inferior Frontal Gyrus | Left Fusiform Gyrus |
| Left Amygdala | Right Anterior Cinguli Gyrus |
| Right Amygdala | Right Superior Temporal Gyrus |
| Right Gyrus Rectus | Left Anterior Medial Temporal Lobe |
| Left Inferior Middle Temporal Gyri | Left Inferior Middle Temporal Gyri |
| Right Lateral Temporal Ventricle | Right Orbito-Frontal Gyri |
| Left Posterior Temporal Lobe | Right Inferior Frontal Gyrus |
| Right Insula | Right Gyrus Rectus |
| Right Anterior Cinguli Gyrus | Right Inferior Lateral Pariatal Lobe |
| Left Fusiform Gyrus | Right Insula |
| Left Lateral Temporal Ventricle | Left Inferior Lateral Pariatal Lobe |
| Left Inferior Lateral Pariatal Lobe | Right Third Ventricle |
| Left Gyrus Rectus | Right Posterior Temporal Lobe |
| Right Inferior Middle Temporal Gyri | Left Hippocampus |
| Right Middle Frontal Gyrus | Left Third Ventricle |
| Left Anterior Cinguli Gyrus | Right Amygdala |
| Right Anterior Medial Temporal Lobe | Right Inferior Middle Temporal Gyri |
| Right Corpus Callosum | Left Thalamus |
| Right Thalamus | Right Superior Frontal Gyrus |
| Left Ambient and Parahippocampus Gyri | Right Hippocampus |
| Right Inferior Lateral Pariatal Lobe | Right Anterior Lateral Temporal Lobe |
| Left Anterior Medial Temporal Lobe | Left Superior Frontal Gyrus |
| Left Caudate Nucleus | Right Fusiform Gyrus |
| Left Corpus Callosum | Left Accumbens Nucleus |
| Right Anterior Lateral Temporal Lobe | Right Ambient and Parahippocampus Gyri |
| Left Postcentral Gyrus | Left Posterior Temporal Lobe |
| Left Middle Frontal Gyrus | Right Middle Frontal Gyrus |
| Right Ambient and Parahippocampus Gyri | Left Brainstem |
| Right Third Ventricle | Left Precentral Gyrus |
| Right Lateral Occipital Lobe | Left Middle Frontal Gyrus |
| Left Third Ventricle | Left Superior Parietal Gyrus |
| Left Lateral Occipital Lobe | Right Lateral Temporal Ventricle |
| Left Superior Frontal Gyrus | Left Postcentral Gyrus |
| Left Precentral Gyrus | Right Thalamus |
| Left Thalamus | Right Lateral Occipital Lobe |
| Right Lingual Gyrus | Right Superior Parietal Gyrus |
| Left Lingual Gyrus | Right Corpus Callosum |
| Left Cuneus | Left Cuneus |
| Right Caudate Nucleus | Left Lateral Occipital Lobe |
| Right Cuneus | Right Lingual Gyrus |
| Right Posterior Cinguli Gyrus | Left Posterior Cinguli Gyrus |
| Right Superior Frontal Gyrus | Right Caudate Nucleus |
| Left Putamen | Right Brainstem |
| Right Fusiform Gyrus | Left Caudate Nucleus |
| Right Precentral Gyrus | Right Postcentral Gyrus |
| Left Superior Parietal Gyrus | Left Corpus Callosum |
| Right Postcentral Gyrus | Right Precentral Gyrus |
| Left Brainstem | Right Posterior Cinguli Gyrus |
| Right Superior Parietal Gyrus | Left Lingual Gyrus |
| Left Accumbens Nucleus | Left Lateral Temporal Ventricle |
| Left Posterior Cinguli Gyrus | Left Putamen |
| Right Cerebellum | Left Cerebellum |
| Left Cerebellum | Right Cuneus |
| Right Brainstem | Left Pallidum |
| Right Pallidum | Right Putamen |
| Right Accumbens Nucleus | Right Cerebellum |
| Right Putamen | Right Accumbens Nucleus |
| Left Pallidum | Right Pallidum |

**S1.6 Ranked features for both the EBM and FBM feature selection methods for the Lpba40 GM model.**

| **EBM** | **FBM** |
| --- | --- |
| Right Inferior Temporal Gyrus | Right Hippocampus |
| Left Hippocampus | Left Inferior Temporal Gyrus |
| Left Inferior Temporal Gyrus | Left Fusiform Gyrus |
| Right Hippocampus | Left Hippocampus |
| Left Fusiform Gyrus | Left Middle Temporal Gyrus |
| Right Fusiform Gyrus | Left Parahippocampal Gyrus |
| Right Angular Gyrus | Right Inferior Temporal Gyrus |
| Right Middle Temporal Gyrus | Right Fusiform Gyrus |
| Right Cingulate Gyrus | Left Superior Temporal Gyrus |
| Left Parahippocampal Gyrus | Left Supramarginal Gyrus |
| Left Middle Temporal Gyrus | Right Middle Temporal Gyrus |
| Right Lingual Gyrus | Right Angular Gyrus |
| Left Putamen | Left Angular Gyrus |
| Left Middle Occipital Gyrus | Right Cingulate Gyrus |
| Left Superior Temporal Gyrus | Left Cingulate Gyrus |
| Right Superior Parietal Gyrus | Left Middle Frontal Gyrus |
| Left Middle Frontal Gyrus | Left Precuneus |
| Left Supramarginal Gyrus | Left Superior Parietal Gyrus |
| Right Supramarginal Gyrus | Right Superior Parietal Gyrus |
| Right Inferior Occipital Gyrus | Right Parahippocampal Gyrus |
| Right Superior Temporal Gyrus | Right Supramarginal Gyrus |
| Right Middle Occipital Gyrus | Right Superior Temporal Gyrus |
| Left Angular Gyrus | Right Middle Frontal Gyrus |
| Left Superior Parietal Gyrus | Right Precuneus |
| Right Superior Frontal Gyrus | Left Superior Occipital Gyrus |
| Left Cingulate Gyrus | Left Superior Frontal Gyrus |
| Left Lingual Gyrus | Right Insula |
| Right Precentral Gyrus | Left Cuneus |
| Left Precuneus | Left Insula |
| Right Parahippocampal Gyrus | Right Inferior Frontal Gyrus |
| Right Postcentral Gyrus | Right Caudate |
| Bothside Cerebellar Lobe | Right Inferior Occipital Gyrus |
| Right Putamen | Right Middle Occipital Gyrus |
| Left Superior Occipital Gyrus | Left Middle Occipital Gyrus |
| Right Superior Occipital Gyrus | Right Superior Frontal Gyrus |
| Left Superior Frontal Gyrus | Left Inferior Occipital Gyrus |
| Right Precuneus | Right Superior Occipital Gyrus |
| Right Caudate | Left Middle Orbitofrontal Gyrus |
| Left Precentral Gyrus | Left Inferior Frontal Gyrus |
| Left Caudate | Left Lateral Orbitofrontal Gyrus |
| Right Middle Frontal Gyrus | Right Putamen |
| Left Postcentral Gyrus | Left Caudate |
| Left Inferior Occipital Gyrus | Right Precentral Gyrus |
| Right Gyrus Rectus | Right Postcentral Gyrus |
| Left Lateral Orbitofrontal Gyrus | Bothside Brainstem |
| Right Insula | Left Putamen |
| Right Cuneus | Right Cuneus |
| Right Lateral Orbitofrontal Gyrus | Left Gyrus Rectus |
| Right Middle Orbitofrontal Gyrus | Right Gyrus Rectus |
| Left Inferior Frontal Gyrus | Left Lingual Gyrus |
| Left Middle Orbitofrontal Gyrus | Bothside Cerebellar Lobe |
| Left Insula | Right Middle Orbitofrontal Gyrus |
| Right Inferior Frontal Gyrus | Right Lateral Orbitofrontal Gyrus |
| Bothside Brainstem | Left Precentral Gyrus |
| Left Gyrus Rectus | Right Lingual Gyrus |
| Left Cuneus | Left Postcentral Gyrus |

**S1.7 Ranked features for both the EBM and FBM feature selection methods for the Neuromorphometrics GM model.**

| **EBM** | **FBM** |
| --- | --- |
| Left Amygdala | Left Amygdala |
| Left Entorhinal Area | Right Hippocampus |
| Right Inferior Temporal Gyrus | Left Fusiform Gyrus |
| Left Thalamus Proper | Left Thalamus Proper |
| Left Fusiform Gyrus | Left Entorhinal Area |
| Right Hippocampus | Left Inferior Temporal Gyrus |
| Left Hippocampus | Left Middle Temporal Gyrus |
| Right Amygdala | Right Thalamus Proper |
| Left Calcarine Cortex | Right Amygdala |
| Right Fusiform Gyrus | Left Hippocampus |
| Right Thalamus Proper | Left Parahippocampus Gyrus |
| Left Parahippocampus Gyrus | Left Temporal Pole |
| Left Middle Temporal Gyrus | Left Basal Forebrain |
| Left Middle Frontal Gyrus | Right Inferior Temporal Gyrus |
| Left Temporal Pole | Right Fusiform Gyrus |
| Right Temporal Pole | Right 3rd Ventricle |
| Left Basal Forebrain | Right Temporal Pole |
| Left Inferior Temporal Gyrus | Left Middle Frontal Gyrus |
| Right Calcarine Cortex | Left 3rd Ventricle |
| Left Middle Occipital Gyrus | Left Supramarginal Gyrus |
| Right Superior Temporal Gyrus | Right Posterior Cingulate Gyrus |
| Right Angular Gyrus | Right Entorhinal Area |
| Right Inferior Occipital Gyrus | Right Middle Temporal Gyrus |
| Right Middle Temporal Gyrus | Left Calcarine Cortex |
| Left Putamen | Left Superior Parietal Lobule |
| Left Central Operculum | Right Angular Gyrus |
| Right Entorhinal Area | Left Precuneus |
| Right Supramarginal Gyrus | Right Posterior Insula |
| Right 3rd Ventricle | Left Transverse Temporal Gyrus |
| Right Supplementary Motor Cortex | Right Basal Forebrain |
| Right Posterior Cingulate Gyrus | Left Middle Occipital Gyrus |
| Left Superior Parietal Lobule | Left Planum Polare |
| Left 4th Ventricle | Right Supramarginal Gyrus |
| Left Precuneus | Right Calcarine Cortex |
| Right Superior Frontal Gyrus | Left Superior Temporal Gyrus |
| Right Lingual Gyrus | Right Superior Parietal Lobule |
| Right Planum Polare | Right Ventral DC |
| Left 3rd Ventricle | Left Superior Frontal Gyrus |
| Right Cerebellar Vermal Lobules VI.VII | Left Angular Gyrus |
| Right 4th Ventricle | Left Ventral DC |
| Left Lateral Ventricle | Left Lateral Ventricle |
| Right Lateral Ventricle | Left Anterior Cingulate Gyrus |
| Right Basal Forebrain | Right Lateral Ventricle |
| Left Frontal Pole | Left Planum Temporale |
| Left Supramarginal Gyrus | Right Superior Frontal Gyrus |
| Left Medial Frontal Cortex | Right Parahippocampus Gyrus |
| Left Cerebral White Matter | Left Middle Cingulate Gyrus |
| Right Middle Cingulate Gyrus | Right Frontal Operculum |
| Right Superior Parietal Lobule | Left Posterior Cingulate Gyrus |
| Left Ventral DC | Left Anterior Orbital Gyrus |
| Right Accumbens | Left Medial Frontal Cortex |
| Left Transverse Temporal Gyrus | Left Lateral Orbital Gyrus |
| Left Supplementary Motor Cortex | Right Precuneus |
| Right Gyrus Rectus | Right Inferior Occipital Gyrus |
| Left Angular Gyrus | Right Middle Frontal Gyrus |
| Right Precentral Gyrus | Right Middle Cingulate Gyrus |
| Left Inferior Occipital Gyrus | Left Posterior Insula |
| Left Planum Polare | Right Middle Occipital Gyrus |
| Right Middle Frontal Gyrus | Right Superior Temporal Gyrus |
| Right Medial Frontal Cortex | Left Inferior Occipital Gyrus |
| Left Superior Temporal Gyrus | Right Cerebral White Matter |
| Left Posterior Insula | Left Cerebral White Matter |
| Left Caudate | Right 4th Ventricle |
| Right Occipital Fusiform Gyrus | Left 4th Ventricle |
| Right Putamen | Right Central Operculum |
| Right Middle Occipital Gyrus | Right Planum Polare |
| Left Superior Frontal Gyrus | Right Anterior Cingulate Gyrus |
| Right Parahippocampus Gyrus | Left Cuneus |
| Left Planum Temporale | Right Medial Frontal Cortex |
| Left Subcallosal Area | Right Occipital Fusiform Gyrus |
| Right Caudate | Right Caudate |
| Right Precuneus | Left Frontal Operculum |
| Left Middle Cingulate Gyrus | Left Supplementary Motor Cortex |
| Left Occipital Fusiform Gyrus | Left Frontal Pole |
| Right Frontal Operculum | Right Superior Frontal Gyrus Medial Segment |
| Right Cerebellum Exterior | Left Occipital Fusiform Gyrus |
| Left Anterior Cingulate Gyrus | Right Subcallosal Area |
| Left Lingual Gyrus | Right Cuneus |
| Left Posterior Orbital Gyrus | Right Triangular Part of the Inferior Frontal Gyrus |
| Left Cerebellum Exterior | Left Parietal Operculum |
| Left Precentral Gyrus | Right Transverse Temporal Gyrus |
| Right Posterior Insula | Right Anterior Insula |
| Left Accumbens | Left Superior Occipital Gyrus |
| Left Medial Orbital Gyrus | Left Anterior Insula |
| Right Planum Temporale | Right Cerebellar Vermal Lobules VI.VII |
| Right Anterior Cingulate Gyrus | Left Lingual Gyrus |
| Left Anterior Orbital Gyrus | Left Opercular Part of the Inferior Frontal Gyrus |
| Right Anterior Orbital Gyrus | Left Superior Frontal Gyrus Medial Segment |
| Left Posterior Cingulate Gyrus | Right Opercular Part of the Inferior Frontal Gyrus |
| Right Ventral DC | Left Cerebellar Vermal Lobules VI.VII |
| Right Cuneus | Right Precentral Gyrus |
| Right Central Operculum | Left Caudate |
| Left Superior Occipital Gyrus | Right Cerebellar Vermal Lobules VIII.X |
| Right Medial Orbital Gyrus | Right Putamen |
| Right Superior Frontal Gyrus Medial Segment | Right Superior Occipital Gyrus |
| Right Precentral Gyrus Medial Segment | Left Medial Orbital Gyrus |
| Right Lateral Orbital Gyrus | Left Subcallosal Area |
| Left Lateral Orbital Gyrus | Left Central Operculum |
| Left Postcentral Gyrus | Left Cerebellar Vermal Lobules VIII.X |
| Right Cerebellar Vermal Lobules I.V | Right Planum Temporale |
| Left Cuneus | Left Cerebellum White Matter |
| Right Inferior Lateral Ventricle | Right Pallidum |
| Right Opercular Part of the Inferior Frontal Gyrus | Left Gyrus Rectus |
| Left Parietal Operculum | Right Lingual Gyrus |
| Right Posterior Orbital Gyrus | Left Posterior Orbital Gyrus |
| Left Brainstem | Right Medial Orbital Gyrus |
| Right Occipital Pole | Right Postcentral Gyrus |
| Left Gyrus Rectus | Right Orbital Part of the Inferior Frontal Gyrus |
| Right Parietal Operculum | Left Triangular Part of the Inferior Frontal Gyrus |
| Left Cerebellar Vermal Lobules VIII.X | Right Parietal Operculum |
| Right Superior Occipital Gyrus | Right Frontal Pole |
| Left Cerebellar Vermal Lobules VI.VII | Left Accumbens |
| Right Postcentral Gyrus | Left Putamen |
| Left Superior Frontal Gyrus Medial Segment | Left Brainstem |
| Right Anterior Insula | Left Cerebellar Vermal Lobules I.V |
| Left Opercular Part of the Inferior Frontal Gyrus | Right Lateral Orbital Gyrus |
| Right Cerebral White Matter | Right Cerebellum Exterior |
| Right Optic Chiasm | Right Supplementary Motor Cortex |
| Right Triangular Part of the Inferior Frontal Gyrus | Right Posterior Orbital Gyrus |
| Right Orbital Part of the Inferior Frontal Gyrus | Right Brainstem |
| Left Anterior Insula | Right Precentral Gyrus Medial Segment |
| Right Transverse Temporal Gyrus | Left Cerebellum Exterior |
| Left Frontal Operculum | Right Accumbens |
| Left Cerebellum White Matter | Left Postcentral Gyrus Medial Segment |
| Right Cerebellum White Matter | Right Anterior Orbital Gyrus |
| Left Occipital Pole | Right Occipital Pole |
| Left Cerebellar Vermal Lobules I.V | Left Precentral Gyrus |
| Right Brainstem | Left Precentral Gyrus Medial Segment |
| Left Orbital Part of the Inferior Frontal Gyrus | Right Inferior Lateral Ventricle |
| Right Subcallosal Area | Right Postcentral Gyrus Medial Segment |
| Left Pallidum | Right Optic Chiasm |
| Right Frontal Pole | Right Gyrus Rectus |
| Left Precentral Gyrus Medial Segment | Left Postcentral Gyrus |
| Left Postcentral Gyrus Medial Segment | Right Cerebellum White Matter |
| Right Postcentral Gyrus Medial Segment | Left Pallidum |
| Left Triangular Part of the Inferior Frontal Gyrus | Right Cerebellar Vermal Lobules I.V |
| Right Pallidum | Left Occipital Pole |
| Right Cerebellar Vermal Lobules VIII.X | Left Orbital Part of the Inferior Frontal Gyrus |
| Left Inferior Lateral Ventricle | Left Inferior Lateral Ventricle |
| Left Optic Chiasm | Left Optic Chiasm |

**S1.8 Ranked features for both the EBM and FBM feature selection methods for the Neuromorphometrics CSF model.**

| **EBM** | **FBM** |
| --- | --- |
| Left Anterior Insula | Left Anterior Cingulate Gyrus |
| Left Frontal Operculum | Left Anterior Insula |
| Left Middle Temporal Gyrus | Left Frontal Operculum |
| Left Planum Polare | Left Transverse Temporal Gyrus |
| Left Posterior Insula | Right Anterior Cingulate Gyrus |
| Left Anterior Cingulate Gyrus | Left Planum Polare |
| Right Hippocampus | Left Posterior Insula |
| Right Anterior Orbital Gyrus | Left Amygdala |
| Left Hippocampus | Left Central Operculum |
| Right Anterior Cingulate Gyrus | Right Transverse Temporal Gyrus |
| Right Transverse Temporal Gyrus | Right Superior Frontal Gyrus Medial Segment |
| Right Planum Temporale | Left Middle Temporal Gyrus |
| Right Cerebral White Matter | Left Parietal Operculum |
| Left Transverse Temporal Gyrus | Left Posterior Orbital Gyrus |
| Right Triangular Part of the Inferior Frontal Gyrus | Right Medial Frontal Cortex |
| Right Anterior Insula | Left Planum Temporale |
| Right Lateral Ventricle | Left Medial Frontal Cortex |
| Right Temporal Pole | Left Superior Frontal Gyrus Medial Segment |
| Right Calcarine Cortex | Right Amygdala |
| Right Lateral Orbital Gyrus | Right Frontal Operculum |
| Right Middle Temporal Gyrus | Left Fusiform Gyrus |
| Right Parietal Operculum | Right Central Operculum |
| Left Amygdala | Left Superior Temporal Gyrus |
| Right Central Operculum | Left Subcallosal Area |
| Left Frontal Pole | Right Middle Temporal Gyrus |
| Left Opercular Part of the Inferior Frontal Gyrus | Right Posterior Orbital Gyrus |
| Right Posterior Insula | Left Opercular Part of the Inferior Frontal Gyrus |
| Right Frontal Operculum | Left Triangular Part of the Inferior Frontal Gyrus |
| Left Posterior Orbital Gyrus | Right Planum Temporale |
| Left Parietal Operculum | Right Posterior Insula |
| Left Central Operculum | Right Anterior Orbital Gyrus |
| Left Middle Frontal Gyrus | Right Parietal Operculum |
| Left Subcallosal Area | Left Angular Gyrus |
| Right Planum Polare | Right Basal Forebrain |
| Left Orbital Part of the Inferior Frontal Gyrus | Right Fusiform Gyrus |
| Right Medial Frontal Cortex | Right Anterior Insula |
| Left Triangular Part of the Inferior Frontal Gyrus | Left Orbital Part of the Inferior Frontal Gyrus |
| Right Amygdala | Right 3rd Ventricle |
| Right Superior Frontal Gyrus Medial Segment | Left Temporal Pole |
| Right Inferior Lateral Ventricle | Left Gyrus Rectus |
| Left Caudate | Left Hippocampus |
| Left Planum Temporale | Right Planum Polare |
| Left Lateral Ventricle | Right Angular Gyrus |
| Right Posterior Orbital Gyrus | Right Superior Temporal Gyrus |
| Right Medial Orbital Gyrus | Left Inferior Temporal Gyrus |
| Right Subcallosal Area | Left Ventral DC |
| Right Thalamus Proper | Left Middle Occipital Gyrus |
| Left Medial Frontal Cortex | Left Supramarginal Gyrus |
| Left Temporal Pole | Left Thalamus Proper |
| Right Middle Frontal Gyrus | Left Middle Frontal Gyrus |
| Right Frontal Pole | Left 3rd Ventricle |
| Right Caudate | Left Inferior Occipital Gyrus |
| Right Gyrus Rectus | Left Entorhinal Area |
| Left Cerebral White Matter | Right Ventral DC |
| Right Accumbens | Right Opercular Part of the Inferior Frontal Gyrus |
| Left Superior Temporal Gyrus | Left Anterior Orbital Gyrus |
| Left Angular Gyrus | Right Lateral Orbital Gyrus |
| Left Supramarginal Gyrus | Right Temporal Pole |
| Left Lateral Orbital Gyrus | Right Hippocampus |
| Right Inferior Occipital Gyrus | Left Medial Orbital Gyrus |
| Left Medial Orbital Gyrus | Right Subcallosal Area |
| Left Inferior Occipital Gyrus | Right Middle Frontal Gyrus |
| Left Middle Occipital Gyrus | Left Parahippocampus Gyrus |
| Left Superior Frontal Gyrus Medial Segment | Right Caudate |
| Right Opercular Part of the Inferior Frontal Gyrus | Right Medial Orbital Gyrus |
| Right Ventral DC | Right Middle Occipital Gyrus |
| Left Fusiform Gyrus | Right Gyrus Rectus |
| Left Calcarine Cortex | Left Lingual Gyrus |
| Right Fusiform Gyrus | Left Postcentral Gyrus |
| Left Gyrus Rectus | Left Precuneus |
| Left Anterior Orbital Gyrus | Right Thalamus Proper |
| Right Superior Temporal Gyrus | Right Cerebral White Matter |
| Right Supramarginal Gyrus | Right Lateral Ventricle |
| Right Basal Forebrain | Left Basal Forebrain |
| Left Inferior Temporal Gyrus | Left Caudate |
| Left Thalamus Proper | Right Frontal Pole |
| Left Lingual Gyrus | Right Optic Chiasm |
| Right 3rd Ventricle | Right Supramarginal Gyrus |
| Right Lingual Gyrus | Left Frontal Pole |
| Right Superior Occipital Gyrus | Left Brainstem |
| Right Angular Gyrus | Right Accumbens |
| Left Accumbens | Right Inferior Occipital Gyrus |
| Right Middle Occipital Gyrus | Right Triangular Part of the Inferior Frontal Gyrus |
| Left Entorhinal Area | Left Posterior Cingulate Gyrus |
| Left Ventral DC | Left Precentral Gyrus |
| Left 3rd Ventricle | Left Superior Parietal Lobule |
| Right Entorhinal Area | Left Occipital Fusiform Gyrus |
| Left CSF | Left Cuneus |
| Left Precentral Gyrus | Right Inferior Lateral Ventricle |
| Right CSF | Right Superior Occipital Gyrus |
| Left Putamen | Right Precuneus |
| Right Optic Chiasm | Right Superior Parietal Lobule |
| Left Parahippocampus Gyrus | Right Inferior Temporal Gyrus |
| Right Cuneus | Left Cerebellum Exterior |
| Right Parahippocampus Gyrus | Right Lingual Gyrus |
| Right Cerebellum Exterior | Right Cerebellum Exterior |
| Left Brainstem | Left Cerebral White Matter |
| Right Posterior Cingulate Gyrus | Right Brainstem |
| Right Precuneus | Left Occipital Pole |
| Right Orbital Part of the Inferior Frontal Gyrus | Left CSF |
| Left Basal Forebrain | Right Superior Frontal Gyrus |
| Right Occipital Fusiform Gyrus | Left Lateral Ventricle |
| Left Postcentral Gyrus | Left Accumbens |
| Right Brainstem | Right 4th Ventricle |
| Right Inferior Temporal Gyrus | Left Lateral Orbital Gyrus |
| Left Occipital Fusiform Gyrus | Right Precentral Gyrus |
| Right Postcentral Gyrus | Right Posterior Cingulate Gyrus |
| Left Precuneus | Left 4th Ventricle |
| Right Superior Parietal Lobule | Right Parahippocampus Gyrus |
| Left Middle Cingulate Gyrus | Right Entorhinal Area |
| Left Cerebellum Exterior | Left Middle Cingulate Gyrus |
| Right Precentral Gyrus | Right CSF |
| Right Superior Frontal Gyrus | Left Superior Frontal Gyrus |
| Left Occipital Pole | Right Middle Cingulate Gyrus |
| Right Occipital Pole | Right Occipital Pole |
| Left Superior Frontal Gyrus | Left Cerebellar Vermal Lobules VIII.X |
| Left Cerebellar Vermal Lobules VI.VII | Right Postcentral Gyrus |
| Left Posterior Cingulate Gyrus | Right Occipital Fusiform Gyrus |
| Left Cuneus | Right Cerebellar Vermal Lobules VIII.X |
| Left Superior Parietal Lobule | Left Superior Occipital Gyrus |
| Left Cerebellum White Matter | Right Orbital Part of the Inferior Frontal Gyrus |
| Left Cerebellar Vermal Lobules VIII.X | Left Pallidum |
| Right Cerebellum White Matter | Right Cerebellum White Matter |
| Left Supplementary Motor Cortex | Left Cerebellum White Matter |
| Left Superior Occipital Gyrus | Right Cuneus |
| Right Middle Cingulate Gyrus | Left Supplementary Motor Cortex |
| Right Cerebellar Vermal Lobules VI.VII | Right Calcarine Cortex |
| Right 4th Ventricle | Left Putamen |
| Left Precentral Gyrus Medial Segment | Right Supplementary Motor Cortex |
| Right Cerebellar Vermal Lobules I.V | Left Cerebellar Vermal Lobules I.V |
| Right Supplementary Motor Cortex | Right Pallidum |
| Right Cerebellar Vermal Lobules VIII.X | Left Cerebellar Vermal Lobules VI.VII |
| Right Precentral Gyrus Medial Segment | Right Cerebellar Vermal Lobules I.V |
| Left 4th Ventricle | Right Postcentral Gyrus Medial Segment |
| Left Cerebellar Vermal Lobules I.V | Right Cerebellar Vermal Lobules VI.VII |
| Left Postcentral Gyrus Medial Segment | Left Postcentral Gyrus Medial Segment |
| Right Postcentral Gyrus Medial Segment | Right Putamen |
| Right Putamen | Left Calcarine Cortex |
| Left Pallidum | Left Precentral Gyrus Medial Segment |
| Right Pallidum | Right Precentral Gyrus Medial Segment |
| Left Inferior Lateral Ventricle | NA |
| Left Optic Chiasm | NA |

**S1.9 Ranked features for both the EBM and FBM feature selection methods for the a2009 Gyrification model.**

| **EBM** | **FBM** |
| --- | --- |
| rS_precentral.sup.part | rS_precentral.inf.part |
| rUnknown | rS_precentral.sup.part |
| rLat_Fis.post | rUnknown |
| rS_temporal_sup | rS_central |
| lUnknown | lG_occipital_sup |
| lG_occipital_sup | lS_circular_insula_inf |
| lS_front_inf | lS_circular_insula_sup |
| rS_central | rLat_Fis.post |
| lS_circular_insula_inf | lUnknown |
| lG_Ins_lg_and_S_cent_ins | rS_orbital.H_Shaped |
| rG_orbital | lS_precentral.inf.part |
| rS_front_middle | lS_central |
| lS_precentral.inf.part | rG_orbital |
| rS_precentral.inf.part | rS_circular_insula_inf |
| lG_and_S_cingul.Mid.Ant | rS_temporal_sup |
| rS_circular_insula_inf | lG_oc.temp_lat.fusifor |
| lG_and_S_cingul.Ant | lG_and_S_paracentral |
| lS_central | rG_and_S_cingul.Mid.Post |
| lPole_occipital | lS_front_inf |
| lG_and_S_subcentral | rG_front_sup |
| lLat_Fis.ant.Horizont | rS_intrapariet_and_P_trans |
| lS_circular_insula_sup | rS_collat_transv_ant |
| lG_insular_short | lS_pericallosal |
| lG_oc.temp_lat.fusifor | lLat_Fis.ant.Vertical |
| lLat_Fis.ant.Vertical | rS_front_sup |
| rG_and_S_subcentral | rG_and_S_subcentral |
| rS_orbital_lateral | lG_and_S_frontomargin |
| lG_occipital_middle | lG_Ins_lg_and_S_cent_ins |
| rS_collat_transv_ant | lG_occipital_middle |
| lG_postcentral | lLat_Fis.post |
| rS_orbital.H_Shaped | rG_front_inf.Triangul |
| rG_front_sup | rPole_occipital |
| rS_calcarine | lG_front_sup |
| rS_front_sup | rS_circular_insula_ant |
| rG_cuneus | lG_and_S_cingul.Ant |
| rG_and_S_cingul.Mid.Post | lS_calcarine |
| rG_temporal_middle | rG_insular_short |
| lS_pericallosal | lS_oc_sup_and_transversal |
| rG_oc.temp_med.Lingual | lLat_Fis.ant.Horizont |
| lS_oc_sup_and_transversal | rS_calcarine |
| lG_and_S_frontomargin | rS_front_inf |
| lPole_temporal | rS_temporal_inf |
| rG_subcallosal | rLat_Fis.ant.Horizont |
| lG_temp_sup.Plan_polar | lS_orbital.H_Shaped |
| rS_front_inf | lG_cingul.Post.ventral |
| lS_suborbital | lS_suborbital |
| lS_front_middle | lG_and_S_cingul.Mid.Ant |
| rS_intrapariet_and_P_trans | rS_oc_middle_and_Lunatus |
| rLat_Fis.ant.Vertical | lG_temp_sup.Lateral |
| lG_front_inf.Opercular | rS_orbital_lateral |
| lLat_Fis.post | lS_orbital_lateral |
| rPole_occipital | lS_occipital_ant |
| lS_subparietal | rG_and_S_cingul.Ant |
| lS_occipital_ant | rS_front_middle |
| lG_and_S_transv_frontopol | lG_cuneus |
| rG_front_inf.Orbital | rG_Ins_lg_and_S_cent_ins |
| lS_calcarine | lS_front_middle |
| rS_temporal_inf | lG_temp_sup.Plan_polar |
| rS_circular_insula_ant | rG_oc.temp_lat.fusifor |
| lS_circular_insula_ant | rG_subcallosal |
| rG_precuneus | lPole_occipital |
| lS_precentral.sup.part | lG_and_S_transv_frontopol |
| lS_oc_middle_and_Lunatus | rG_front_middle |
| rG_insular_short | lG_cingul.Post.dorsal |
| rS_oc.temp_med_and_Lingual | rS_oc.temp_med_and_Lingual |
| rS_oc_middle_and_Lunatus | rS_subparietal |
| lG_pariet_inf.Angular | lS_collat_transv_ant |
| lG_cingul.Post.dorsal | rS_oc_sup_and_transversal |
| rG_rectus | rG_oc.temp_med.Lingual |
| lS_collat_transv_ant | rS_interm_prim.Jensen |
| lG_and_S_paracentral | lS_front_sup |
| rG_front_inf.Opercular | rG_cingul.Post.ventral |
| lG_front_middle | lG_pariet_inf.Angular |
| lG_cuneus | rG_cuneus |
| lG_temporal_middle | rG_temp_sup.G_T_transv |
| rG_and_S_occipital_inf | lG_front_middle |
| rS_subparietal | lS_temporal_sup |
| rG_postcentral | lS_parieto_occipital |
| lG_and_S_occipital_inf | lG_parietal_sup |
| rG_front_inf.Triangul | rG_and_S_transv_frontopol |
| rS_collat_transv_post | lG_and_S_subcentral |
| lG_subcallosal | rG_precentral |
| lS_intrapariet_and_P_trans | lS_temporal_transverse |
| lG_temp_sup.Plan_tempo | rLat_Fis.ant.Vertical |
| rLat_Fis.ant.Horizont | rS_collat_transv_post |
| lG_pariet_inf.Supramar | rG_front_inf.Orbital |
| lS_orbital.H_Shaped | lS_intrapariet_and_P_trans |
| lS_orbital_lateral | rG_occipital_middle |
| lG_front_sup | lG_pariet_inf.Supramar |
| rG_Ins_lg_and_S_cent_ins | rG_temporal_inf |
| lS_temporal_transverse | rS_cingul.Marginalis |
| lG_front_inf.Orbital | rG_and_S_cingul.Mid.Ant |
| rG_front_middle | lG_front_inf.Opercular |
| lG_cingul.Post.ventral | rG_postcentral |
| rS_circular_insula_sup | lG_subcallosal |
| rS_pericallosal | lG_oc.temp_med.Lingual |
| rS_parieto_occipital | rG_and_S_occipital_inf |
| rG_cingul.Post.ventral | rS_circular_insula_sup |
| lS_front_sup | rG_temp_sup.Lateral |
| rG_temp_sup.G_T_transv | rG_temp_sup.Plan_tempo |
| lS_temporal_sup | rS_postcentral |
| rG_temporal_inf | lS_collat_transv_post |
| rS_interm_prim.Jensen | lS_circular_insula_ant |
| lS_parieto_occipital | lG_precentral |
| rG_occipital_sup | lS_cingul.Marginalis |
| rG_and_S_cingul.Ant | rS_pericallosal |
| rG_temp_sup.Lateral | lG_and_S_occipital_inf |
| rG_oc.temp_lat.fusifor | lG_temp_sup.Plan_tempo |
| lG_front_inf.Triangul | rG_and_S_paracentral |
| lG_and_S_cingul.Mid.Post | lS_subparietal |
| rS_orbital_med.olfact | lS_oc_middle_and_Lunatus |
| lS_orbital_med.olfact | rG_cingul.Post.dorsal |
| rS_oc.temp_lat | lG_temporal_inf |
| lG_oc.temp_med.Lingual | lS_temporal_inf |
| rG_and_S_cingul.Mid.Ant | lG_temp_sup.G_T_transv |
| rPole_temporal | rG_oc.temp_med.Parahip |
| lG_temp_sup.Lateral | rPole_temporal |
| rS_temporal_transverse | lS_postcentral |
| rS_cingul.Marginalis | rG_temporal_middle |
| lS_interm_prim.Jensen | lG_rectus |
| rS_suborbital | rS_parieto_occipital |
| lG_parietal_sup | lG_postcentral |
| rG_pariet_inf.Supramar | rG_rectus |
| lG_precuneus | lG_insular_short |
| rG_and_S_transv_frontopol | rG_occipital_sup |
| rS_oc_sup_and_transversal | rS_occipital_ant |
| lG_orbital | rG_and_S_frontomargin |
| rS_postcentral | lG_front_inf.Triangul |
| rG_and_S_paracentral | lG_front_inf.Orbital |
| rG_occipital_middle | lG_precuneus |
| rG_and_S_frontomargin | lS_orbital_med.olfact |
| rG_oc.temp_med.Parahip | lS_precentral.sup.part |
| rG_precentral | rS_suborbital |
| lG_temporal_inf | rS_orbital_med.olfact |
| rG_parietal_sup | rG_front_inf.Opercular |
| lS_cingul.Marginalis | lPole_temporal |
| lS_collat_transv_post | rG_precuneus |
| rG_pariet_inf.Angular | lG_temporal_middle |
| lG_oc.temp_med.Parahip | rG_pariet_inf.Supramar |
| lG_rectus | lS_interm_prim.Jensen |
| rG_cingul.Post.dorsal | rS_oc.temp_lat |
| lS_oc.temp_lat | lS_oc.temp_lat |
| lS_temporal_inf | lG_and_S_cingul.Mid.Post |
| rG_temp_sup.Plan_polar | rG_pariet_inf.Angular |
| rS_occipital_ant | rS_temporal_transverse |
| rG_temp_sup.Plan_tempo | lG_oc.temp_med.Parahip |
| lG_precentral | lG_orbital |
| lG_temp_sup.G_T_transv | rG_parietal_sup |
| lS_oc.temp_med_and_Lingual | lS_oc.temp_med_and_Lingual |
| lS_postcentral | rG_temp_sup.Plan_polar |

**S1.10 Ranked features for both the EBM and FBM feature selection methods for the a2009 Thickness model.**

| **EBM** | **FBM** |
| --- | --- |
| lG_pariet_inf.Supramar | lUnknown |
| lG_temp_sup.Plan_tempo | lG_pariet_inf.Supramar |
| lG_temp_sup.Plan_polar | lG_and_S_cingul.Mid.Ant |
| lG_and_S_cingul.Mid.Ant | lS_front_middle |
| lPole_temporal | lG_oc.temp_med.Parahip |
| lUnknown | lG_temp_sup.Plan_tempo |
| lS_front_middle | lG_pariet_inf.Angular |
| lS_intrapariet_and_P_trans | lG_temp_sup.Plan_polar |
| rS_front_inf | rG_temp_sup.Lateral |
| rG_pariet_inf.Angular | lG_front_inf.Triangul |
| lG_pariet_inf.Angular | rG_pariet_inf.Angular |
| lG_front_middle | rS_circular_insula_inf |
| rG_temp_sup.Lateral | lG_cingul.Post.dorsal |
| lG_oc.temp_med.Parahip | lG_and_S_cingul.Mid.Post |
| rG_cingul.Post.dorsal | rS_front_inf |
| rUnknown | rUnknown |
| rS_circular_insula_inf | lG_temp_sup.Lateral |
| lS_front_inf | rG_cingul.Post.ventral |
| lG_temp_sup.Lateral | lG_Ins_lg_and_S_cent_ins |
| rS_temporal_sup | rG_and_S_cingul.Mid.Ant |
| lG_cingul.Post.dorsal | lPole_temporal |
| rG_cingul.Post.ventral | rG_temporal_middle |
| lG_Ins_lg_and_S_cent_ins | rS_temporal_sup |
| rG_and_S_cingul.Mid.Ant | lS_intrapariet_and_P_trans |
| rLat_Fis.ant.Vertical | rG_cingul.Post.dorsal |
| lLat_Fis.post | rG_Ins_lg_and_S_cent_ins |
| lG_front_inf.Triangul | lS_circular_insula_sup |
| rG_temporal_middle | lG_front_middle |
| lS_circular_insula_inf | lG_front_inf.Opercular |
| lG_parietal_sup | rG_pariet_inf.Supramar |
| rG_oc.temp_med.Parahip | lS_cingul.Marginalis |
| lS_orbital_lateral | lG_temporal_middle |
| lG_temporal_middle | rS_precentral.inf.part |
| lPole_occipital | rS_collat_transv_ant |
| rG_pariet_inf.Supramar | rG_parietal_sup |
| rG_front_middle | rS_oc_sup_and_transversal |
| lG_front_inf.Opercular | rG_temp_sup.Plan_polar |
| rS_collat_transv_ant | lS_circular_insula_inf |
| lG_and_S_cingul.Mid.Post | lG_and_S_transv_frontopol |
| rG_precuneus | rG_insular_short |
| rS_oc_sup_and_transversal | lLat_Fis.post |
| rS_cingul.Marginalis | lG_cingul.Post.ventral |
| lS_cingul.Marginalis | rLat_Fis.post |
| lS_circular_insula_sup | rG_oc.temp_med.Parahip |
| lG_insular_short | rLat_Fis.ant.Vertical |
| rG_Ins_lg_and_S_cent_ins | lS_temporal_sup |
| lG_cingul.Post.ventral | lS_front_inf |
| rS_orbital.H_Shaped | lG_parietal_sup |
| rG_parietal_sup | rS_orbital.H_Shaped |
| lG_occipital_sup | rS_interm_prim.Jensen |
| lS_circular_insula_ant | rG_occipital_middle |
| lS_orbital.H_Shaped | lS_orbital.H_Shaped |
| rS_subparietal | lG_insular_short |
| rLat_Fis.post | lG_temp_sup.G_T_transv |
| rS_precentral.inf.part | lS_circular_insula_ant |
| lG_temporal_inf | rG_front_middle |
| rS_front_middle | rG_temp_sup.Plan_tempo |
| lS_temporal_sup | rS_parieto_occipital |
| rS_interm_prim.Jensen | lS_parieto_occipital |
| lG_and_S_transv_frontopol | rG_temporal_inf |
| lS_parieto_occipital | lG_precuneus |
| lG_and_S_subcentral | lS_front_sup |
| rS_pericallosal | rS_postcentral |
| rG_occipital_middle | rG_temp_sup.G_T_transv |
| rG_insular_short | lS_orbital_lateral |
| rG_temporal_inf | rS_intrapariet_and_P_trans |
| rS_precentral.sup.part | rG_oc.temp_lat.fusifor |
| lS_pericallosal | lG_and_S_frontomargin |
| rG_and_S_transv_frontopol | lS_collat_transv_ant |
| rG_oc.temp_lat.fusifor | rS_circular_insula_sup |
| lG_cuneus | rS_temporal_inf |
| lG_precuneus | rS_orbital_lateral |
| lS_postcentral | lG_temporal_inf |
| lG_orbital | rS_front_middle |
| rS_orbital_lateral | lLat_Fis.ant.Vertical |
| lG_and_S_frontomargin | rS_subparietal |
| rS_postcentral | lG_oc.temp_lat.fusifor |
| rG_temp_sup.Plan_polar | rS_cingul.Marginalis |
| rS_oc_middle_and_Lunatus | rG_oc.temp_med.Lingual |
| lS_front_sup | rPole_temporal |
| lS_oc.temp_med_and_Lingual | lS_postcentral |
| rG_front_sup | rG_precuneus |
| rS_intrapariet_and_P_trans | rG_and_S_transv_frontopol |
| lS_oc_sup_and_transversal | rG_occipital_sup |
| rS_parieto_occipital | rS_pericallosal |
| rG_temp_sup.G_T_transv | rG_front_inf.Opercular |
| rG_temp_sup.Plan_tempo | lS_precentral.inf.part |
| lS_collat_transv_ant | lS_interm_prim.Jensen |
| rG_front_inf.Opercular | lS_pericallosal |
| rG_oc.temp_med.Lingual | lPole_occipital |
| rG_occipital_sup | lS_oc.temp_med_and_Lingual |
| lS_precentral.sup.part | rS_front_sup |
| rS_temporal_inf | rLat_Fis.ant.Horizont |
| lG_occipital_middle | lS_occipital_ant |
| rS_occipital_ant | rG_and_S_frontomargin |
| rG_front_inf.Triangul | rG_front_sup |
| lG_temp_sup.G_T_transv | lS_oc_sup_and_transversal |
| lG_and_S_cingul.Ant | lG_and_S_subcentral |
| rS_circular_insula_sup | lG_orbital |
| rG_rectus | lG_front_sup |
| lG_precentral | rG_subcallosal |
| rS_front_sup | lG_and_S_cingul.Ant |
| lLat_Fis.ant.Vertical | lG_occipital_middle |
| rG_and_S_frontomargin | lS_precentral.sup.part |
| lG_front_sup | rS_circular_insula_ant |
| lS_interm_prim.Jensen | lG_front_inf.Orbital |
| rPole_occipital | lS_subparietal |
| lS_calcarine | lS_temporal_transverse |
| lLat_Fis.ant.Horizont | lG_rectus |
| lS_precentral.inf.part | rG_and_S_cingul.Mid.Post |
| lG_and_S_paracentral | rG_precentral |
| lG_oc.temp_lat.fusifor | rG_postcentral |
| rG_precentral | lS_oc.temp_lat |
| rPole_temporal | rG_and_S_subcentral |
| lS_temporal_inf | rG_orbital |
| rG_and_S_subcentral | rS_orbital_med.olfact |
| lS_temporal_transverse | rS_oc_middle_and_Lunatus |
| lG_front_inf.Orbital | rPole_occipital |
| rG_front_inf.Orbital | rS_temporal_transverse |
| lG_postcentral | lS_collat_transv_post |
| rS_calcarine | rG_front_inf.Triangul |
| lS_collat_transv_post | lG_subcallosal |
| rG_and_S_cingul.Mid.Post | rS_oc.temp_med_and_Lingual |
| rS_suborbital | lS_temporal_inf |
| rS_central | lG_cuneus |
| lS_orbital_med.olfact | rS_collat_transv_post |
| rS_oc.temp_lat | rG_and_S_cingul.Ant |
| lG_rectus | lLat_Fis.ant.Horizont |
| rG_and_S_paracentral | rG_rectus |
| rG_and_S_cingul.Ant | lG_and_S_paracentral |
| lS_central | lG_precentral |
| lG_subcallosal | rG_cuneus |
| lS_subparietal | rS_oc.temp_lat |
| lG_and_S_occipital_inf | rS_occipital_ant |
| rS_collat_transv_post | lS_calcarine |
| lS_oc_middle_and_Lunatus | lG_oc.temp_med.Lingual |
| rG_orbital | lS_central |
| rS_orbital_med.olfact | lS_orbital_med.olfact |
| lS_suborbital | rG_and_S_occipital_inf |
| rLat_Fis.ant.Horizont | rS_precentral.sup.part |
| rG_and_S_occipital_inf | lS_oc_middle_and_Lunatus |
| rG_postcentral | rS_suborbital |
| rG_cuneus | lS_suborbital |
| lS_occipital_ant | lG_occipital_sup |
| rS_circular_insula_ant | lG_postcentral |
| rS_temporal_transverse | lG_and_S_occipital_inf |
| lG_oc.temp_med.Lingual | rG_and_S_paracentral |
| rS_oc.temp_med_and_Lingual | rS_calcarine |
| rG_subcallosal | rG_front_inf.Orbital |
| lS_oc.temp_lat | rS_central |

**S1.11 Ranked features for both the EBM and FBM feature selection methods for the DK40 Gyrification model.**

| **EBM** | **FBM** |
| --- | --- |
| rlateralorbitofrontal | rlateralorbitofrontal |
| rprecentral | rprecentral |
| rinsula | rsuperiorfrontal |
| rrostralmiddlefrontal | rsuperiortemporal |
| rsuperiorfrontal | lrostralmiddlefrontal |
| lcaudalanteriorcingulate | lcaudalanteriorcingulate |
| lrostralmiddlefrontal | lsuperiorfrontal |
| rsuperiortemporal | lrostralanteriorcingulate |
| rparstriangularis | listhmuscingulate |
| rparahippocampal | rsuperiorparietal |
| lpericalcarine | lparstriangularis |
| rsuperiorparietal | lpericalcarine |
| linsula | linsula |
| lsuperiorfrontal | rparstriangularis |
| lrostralanteriorcingulate | rparsopercularis |
| lposteriorcingulate | rsupramarginal |
| llateraloccipital | rrostralmiddlefrontal |
| rparacentral | llateraloccipital |
| rbankssts | ltransversetemporal |
| llateralorbitofrontal | lprecentral |
| listhmuscingulate | lentorhinal |
| rlingual | lsuperiortemporal |
| rsupramarginal | rinsula |
| rmiddletemporal | rposteriorcingulate |
| rposteriorcingulate | llateralorbitofrontal |
| rparsopercularis | rpostcentral |
| lparstriangularis | rmiddletemporal |
| rpericalcarine | rtransversetemporal |
| lpostcentral | lparahippocampal |
| rrostralanteriorcingulate | rinferiortemporal |
| lentorhinal | rlateraloccipital |
| lsuperiortemporal | rpericalcarine |
| llingual | rparahippocampal |
| linferiortemporal | lbankssts |
| rpostcentral | lpostcentral |
| lprecentral | rbankssts |
| rprecuneus | rcaudalmiddlefrontal |
| lunknown | rrostralanteriorcingulate |
| rlateraloccipital | lcaudalmiddlefrontal |
| linferiorparietal | lcuneus |
| rcaudalmiddlefrontal | rlingual |
| rtemporalpole | lmedialorbitofrontal |
| ltemporalpole | rparacentral |
| rinferiortemporal | rparsorbitalis |
| rparsorbitalis | rcaudalanteriorcingulate |
| lmiddletemporal | linferiortemporal |
| rtransversetemporal | rprecuneus |
| lbankssts | lposteriorcingulate |
| lmedialorbitofrontal | rentorhinal |
| lfusiform | ltemporalpole |
| ltransversetemporal | lparsopercularis |
| rcaudalanteriorcingulate | lparsorbitalis |
| rmedialorbitofrontal | risthmuscingulate |
| rfusiform | lunknown |
| lparacentral | llingual |
| lfrontalpole | lparacentral |
| lcuneus | rtemporalpole |
| lprecuneus | lfusiform |
| rcuneus | lsuperiorparietal |
| rinferiorparietal | lsupramarginal |
| rfrontalpole | lprecuneus |
| risthmuscingulate | linferiorparietal |
| lcaudalmiddlefrontal | lmiddletemporal |
| lparsopercularis | lfrontalpole |
| rentorhinal | rfusiform |
| lsupramarginal | rinferiorparietal |
| lparahippocampal | rcuneus |
| lsuperiorparietal | rmedialorbitofrontal |
| lparsorbitalis | rfrontalpole |

**S1.12 Ranked features for both the EBM and FBM feature selection methods for the DK40 Thickness model.**

| **EBM** | **FBM** |
| --- | --- |
| lsupramarginal | lposteriorcingulate |
| ltemporalpole | lentorhinal |
| lentorhinal | lsupramarginal |
| lposteriorcingulate | lsuperiortemporal |
| rinferiorparietal | rtemporalpole |
| lparsopercularis | rinferiorparietal |
| linsula | rinsula |
| linferiorparietal | lrostralmiddlefrontal |
| lcaudalmiddlefrontal | rmiddletemporal |
| lrostralmiddlefrontal | rsuperiortemporal |
| lsuperiortemporal | linferiorparietal |
| linferiortemporal | linsula |
| rinsula | rsupramarginal |
| lsuperiorparietal | lparsopercularis |
| lmiddletemporal | lparstriangularis |
| rsupramarginal | lmiddletemporal |
| rpericalcarine | risthmuscingulate |
| rsuperiortemporal | listhmuscingulate |
| listhmuscingulate | ltemporalpole |
| rtemporalpole | rsuperiorparietal |
| rmiddletemporal | rcaudalanteriorcingulate |
| rcaudalanteriorcingulate | rbankssts |
| rrostralmiddlefrontal | lfusiform |
| rfusiform | rrostralmiddlefrontal |
| lparstriangularis | lcaudalmiddlefrontal |
| rsuperiorfrontal | rfusiform |
| risthmuscingulate | ltransversetemporal |
| rparsopercularis | lsuperiorparietal |
| lprecentral | lprecuneus |
| rparahippocampal | rparsopercularis |
| llateralorbitofrontal | linferiortemporal |
| rinferiortemporal | rparahippocampal |
| rentorhinal | lparsorbitalis |
| rsuperiorparietal | rprecuneus |
| lfrontalpole | lfrontalpole |
| rbankssts | rposteriorcingulate |
| lparahippocampal | rsuperiorfrontal |
| rposteriorcingulate | lcaudalanteriorcingulate |
| rcaudalmiddlefrontal | rinferiortemporal |
| rlateralorbitofrontal | rtransversetemporal |
| lprecuneus | lrostralanteriorcingulate |
| rprecuneus | rpericalcarine |
| lfusiform | rparstriangularis |
| lcaudalanteriorcingulate | rlingual |
| lpericalcarine | rcaudalmiddlefrontal |
| lsuperiorfrontal | lsuperiorfrontal |
| ltransversetemporal | lparahippocampal |
| rparstriangularis | llateralorbitofrontal |
| rfrontalpole | llingual |
| lrostralanteriorcingulate | rentorhinal |
| llateraloccipital | rlateralorbitofrontal |
| lcuneus | lunknown |
| rlingual | lbankssts |
| lbankssts | rpostcentral |
| rparacentral | lpericalcarine |
| rtransversetemporal | rcuneus |
| lparsorbitalis | rprecentral |
| rpostcentral | lprecentral |
| lpostcentral | llateraloccipital |
| lmedialorbitofrontal | lmedialorbitofrontal |
| lparacentral | lparacentral |
| rprecentral | rparsorbitalis |
| llingual | rlateraloccipital |
| rrostralanteriorcingulate | rmedialorbitofrontal |
| rmedialorbitofrontal | lcuneus |
| rlateraloccipital | lpostcentral |
| rcuneus | rparacentral |
| lunknown | rfrontalpole |
| rparsorbitalis | rrostralanteriorcingulate |

**S1.13 Ranked features for both the EBM and FBM feature selection methods for the HCP Gyrification model.**

| **EBM** | | | | | **FBM** | | | | |
| --- | --- | --- | --- | --- | --- | --- | --- | --- | --- |
| rMI | rFOP2 | lp47r | lPHA1 | la47r | r6r | rAIP | rA4 | rp24pr | r5m |
| rOP1 | lVMV1 | rTE2p | l44 | rTPOJ2 | rMI | lMT | rSTGa | lv23ab | lIFSp |
| r6r | l55b | rTF | l52 | lV6A | rOP1 | l8Ad | rPoI2 | rMBelt | la47r |
| rSTSda | rIFSa | rEC | ld23ab | lOP4 | rSTSda | r9a | lLO3 | l9m | l25 |
| lIg | lMT | rp32 | rp24pr | r44 | r8Ad | r6v | lMIP | l6d | r6d |
| r8Ad | rPHA2 | rPOS2 | rFFC | rpOFC | rLIPv | lFOP5 | rIPS1 | rVIP | l3b |
| r4 | rV4 | rv23ab | rPSL | ri6.8 | lPir | ld32 | lPGs | la24pr | lFEF |
| lIFJp | rIFJp | rPF | l8BL | rTGv | lp10p | lV3A | li6.8 | r10v | rPF |
| r7Pm | rV2 | lIFJa | rs32 | rPHA3 | lIFJp | lIP1 | l10d | rV1 | lPreS |
| lV7 | rA5 | rPFcm | l8C | lTPOJ3 | l3a | rPFt | rFFC | lPHA3 | lLO2 |
| lp32pr | lFOP2 | lp9.46v | l6mp | r24dv | r4 | r23c | r2 | lOFC | rTPOJ2 |
| lTPOJ1 | l46 | r47m | lVMV2 | l5L | rSCEF | lPGp | r24dv | rTPOJ1 | l24dd |
| lPir | lSCEF | rPoI1 | rV7 | lV6 | la24 | r13l | lV3B | lPFop | l5m |
| rSCEF | r6v | rProS | lPoI2 | l5mv | r3a | rIg | lTA2 | rPHT | r10r |
| lp10p | r13l | lSTSva | lPreS | rV3A | rIFSp | rOP4 | lPFm | lV3CD | lLO1 |
| rIg | rFOP3 | lMI | lLO1 | l8Av | l9a | rVMV2 | lH | rTE2p | rV3B |
| lSTSda | rIP2 | lv23ab | r31a | lFOP1 | ra24 | rFOP5 | ld23ab | r23d | lpOFC |
| rPBelt | r43 | rp32pr | lPFt | lIPS1 | lIFSa | r10pp | rPEF | l44 | lAAIC |
| la9.46v | l10r | r45 | rV1 | lIP2 | rp9.46v | l23d | lVMV2 | lLIPv | r5L |
| lIFSa | r1 | lAIP | rPGi | rPFm | lAVI | r24dd | l6mp | r6ma | rV7 |
| rRI | rPFt | r9a | l6v | lPeEc | r7Pm | rA5 | rIP0 | rPOS1 | l43 |
| rAIP | la10p | lOFC | lPFcm | rV6A | lTPOJ1 | r31pv | rLO2 | lPH | rV8 |
| rPI | rOP4 | l6ma | lPOS2 | l7Pm | rp10p | l46 | lPFcm | l8BM | rPGi |
| lFFC | l9p | lSTGa | rTE2a | l7AL | rPBelt | r9m | rPOS2 | ls32 | lFOP1 |
| lAVI | lTE1p | rA4 | r8C | l43 | rFOP2 | rV4 | r31pd | r10d | rSTSvp |
| lA5 | rAAIC | rMBelt | rPEF | r7AL | lIg | r45 | lTPOJ3 | rpOFC | lPEF |
| ra24 | r9p | l7PL | r7m | lIFSp | lOP1 | lVVC | r1 | lTE2p | rMT |
| rd32 | lOP2.3 | rPeEc | lPF | r5L | lVMV3 | l9p | rH | r25 | r31a |
| l3a | rVMV1 | l8Ad | lV3 | lPHA3 | r11l | rIFJp | l9.46d | rSFL | lTGd |
| lV3A | r6a | lPEF | rV4t | l9.46d | lFFC | rs6.8 | rPHA2 | rOFC | r47l |
| r23c | lFOP5 | rSTSdp | rFOP4 | rH | lRI | l47m | lV1 | rs32 | l7Am |
| rIFSp | r23d | r31pd | rLO1 | l47s | rIFJa | lSTV | l6r | r5mv | rV4t |
| rs6.8 | l23c | rV3B | r8BL | lPOS1 | lV7 | rOP2.3 | l33pr | rPIT | rTGd |
| l10v | lPHA2 | ra24pr | rd23ab | r47l | lV2 | lMI | r8BM | rd23ab | lPCV |
| rp10p | rFST | lEC | lVIP | lSTSdp | lPI | lEC | rTE1m | lp24pr | lPGi |
| l9a | lPI | lPBelt | lFOP4 | l6d | r47s | lSTSda | lVIP | r43 | rTE1p |
| rLIPd | rPreS | lH | lV4 | rLBelt | lA5 | rA1 | r9.46d | rDVT | lOP4 |
| rLIPv | r9m | lV1 | l2 | lFST | lProS | l31pv | rEC | lPBelt | l31pd |
| la24 | rPGs | lSTSvp | rSFL | r7Am | lFOP2 | lPoI1 | ra47r | rMST | l8Av |
| r33pr | r2 | l23d | l24dd | lPHT | la10p | l8C | lA1 | lPFt | rLO1 |
| lOP1 | rIFJa | lA1 | r25 | l10d | l45 | rPFcm | lLIPd | rTA2 | r44 |
| r24dd | lPoI1 | li6.8 | rp47r | r7PL | l4 | rV2 | rPreS | l6ma | lp32 |
| rRSC | rDVT | rSTGa | r10v | rTE1p | lMST | rSTSdp | lPHT | lDVT | lPOS2 |
| lFOP3 | r10r | lTE2p | r6ma | lp32 | rRI | rp32 | lSTGa | ri6.8 | ra9.46v |
| lVMV3 | rPHT | rPFop | lMBelt | r46 | rFOP3 | lV8 | rp24 | l6v | r7AL |
| lPGp | lTE1m | lLO3 | rV6 | l47l | rPI | l55b | l24dv | r7Am | rV6A |
| lIP1 | rTPOJ1 | rA1 | l3b | rPCV | rProS | rPFop | lPHA1 | lFST | lPF |
| rIP1 | l11l | rMST | rVMV3 | rMT | rIP2 | lIFJa | rMIP | l8BL | rPCV |
| rp9.46v | la32pr | rIPS1 | lpOFC | r5mv | r52 | rAVI | rVVC | rTPOJ3 | lPeEc |
| ld32 | l6a | rAVI | rVIP | rPIT | r6mp | lp47r | rV6 | lSTSdp | lp9.46v |
| rV3CD | r10d | l31pd | rFOP1 | lV4t | rLIPd | rPeEc | l2 | rTE2a | l5L |
| r47s | lPFm | rSTSva | ra32pr | lTE1a | l52 | rPH | rSTV | lV6A | rVMV1 |
| lTGd | lVVC | rMIP | lTA2 | lp24pr | l1 | lIP0 | l5mv | r8Av | rLBelt |
| lMST | rOP2.3 | r5m | lPGi | lAAIC | rIFSa | lV3 | l10v | lRSC | lPOS1 |
| r3a | lPGs | l24dv | l31a | rPHA1 | r6a | rFOP1 | rSTSva | r8BL | l7PL |
| r52 | rIP0 | rFEF | r10pp | r55b | lVMV1 | r9p | lLBelt | rFOP4 | r7PL |
| lRI | r9.46d | ra47r | l8BM | r3b | lTE1p | r47m | rV3A | l31a | l7m |
| r11l | lIP0 | lMIP | lPSL | l10pp | rRSC | ra24pr | l6a | rPHA1 | lAIP |
| rPH | ra9.46v | rTGd | rTPOJ3 | lTPOJ2 | lOP2.3 | l11l | rd32 | r7PC | lV6 |
| l4 | lPFop | lDVT | r8Av | l7Am | rIP1 | lTE1m | la32pr | lIPS1 | lPSL |
| r8BM | lV8 | rPGp | rVVC | ls32 | lp32pr | l13l | lV4 | l10pp | rPFm |
| r6mp | rVMV2 | l33pr | rV8 | lFEF | l10r | lMBelt | rFEF | rFST | rTGv |
| lProS | l6r | lPCV | rPir | lSFL | ls6.8 | rPSL | rTE1a | r55b | lV4t |
| ls6.8 | rp24 | lLO2 | rOFC | lTE2a | rp32pr | lPoI2 | lA4 | rPHA3 | lIP2 |
| l45 | l13l | lPH | la24pr | l25 | lSCEF | rAAIC | rv23ab | r3b | lTE2a |
| l47m | lA4 | lLIPd | r6d | rSTSvp | rV3CD | rPir | rLO3 | r7m | ra32pr |
| rTA2 | rPoI2 | rTE1a | l7m | lTF | la9.46v | lp24 | l47s | lSTSvp | ra10p |
| lSTV | r7PC | lLBelt | l7PC | lLIPv | rV3 | lSTSva | r33pr | rVMV3 | r46 |
| rFOP5 | rLO3 | lV3B | lTGv | l5m | rPGp | rp47r | rPGs | r8C | lTE1a |
| lV2 | rV3 | rSTV | lV3CD | lPIT | rPoI1 | l47l | lFOP4 | lPIT | lTPOJ2 |
| l31pv | r31pv | lp24 | lRSC | rLO2 | rTF | lPHA2 | lSFL | l7AL | l7PC |
| l1 | ra10p | l9m | rPOS1 | rTE1m | lFOP3 | l7Pm | lTF | lTGv | l23c |

**S1.14 Ranked features for both the EBM and FBM feature selection methods for the HCP Thickness model.**

| **EBM** | | | | | **FBM** | | | | |
| --- | --- | --- | --- | --- | --- | --- | --- | --- | --- |
| lIP2 | rPOS2 | rLIPd | r8Ad | l5m | lTA2 | lp24 | lFOP1 | rA1 | lpOFC |
| lPF | l7PL | l47s | r3b | rA4 | lPF | l52 | lPFt | rLIPd | rOP1 |
| rPoI1 | rTGd | lFOP3 | r8Av | r45 | l23d | rSTSva | rIPS1 | rPEF | r25 |
| rPGi | r13l | lV1 | rFOP1 | l1 | lIP2 | r23d | r31pv | r7m | r6v |
| r9a | lPoI2 | r8BL | rs32 | rPIT | rPGs | lAIP | lPI | rFFC | r6mp |
| lPFm | rp24 | rp10p | r5m | l10r | r9a | rTA2 | l7PC | rLIPv | l2 |
| lTA2 | lVMV1 | lV7 | lPoI1 | rFOP3 | rPoI1 | r9.46d | rv23ab | rAIP | lTPOJ1 |
| r52 | rSTSvp | lTF | l8BM | rOP1 | lPeEc | rSTSvp | r46 | l47s | lV3A |
| rp9.46v | lRI | r8BM | r6d | rFOP2 | lp32pr | rA5 | r8Ad | ra32pr | r5L |
| rPGs | lAAIC | rSTGa | rPEF | rTPOJ3 | rVMV1 | r7PC | la32pr | rIFSp | l31pv |
| lTGv | rIP2 | lV3A | l6a | l33pr | rPGi | lIP0 | rp9.46v | l31a | lPIT |
| lTGd | l46 | rpOFC | rPreS | l31a | lSTGa | r8C | lPEF | lLO2 | lV3B |
| lPSL | ra24pr | rTE1a | lTPOJ2 | r47m | rPHT | rPI | l8C | l7AL | lTPOJ3 |
| rVMV1 | r9m | l7m | r7Pm | lSTSvp | lPSL | lIFSp | ra10p | lLBelt | l6a |
| lPGs | lPir | lVMV3 | rTE2p | rPBelt | lA5 | rIFJp | lAVI | lOP4 | lPHA3 |
| rp32pr | lIFSp | l8BL | lV3B | rFOP4 | lPGs | lVMV1 | lTPOJ2 | r11l | l47l |
| lPeEc | lTE1a | lPOS1 | l10d | lTE2a | l9.46d | lp9.46v | rIg | rs32 | rOP4 |
| lSTGa | lPH | l11l | rPFt | rVMV2 | lTE1a | lLIPv | r8BL | lV7 | l13l |
| l23d | r31a | lFST | rV2 | rFEF | lDVT | rRI | r23c | rFOP2 | lOFC |
| l9a | l45 | rIg | rV3CD | r3a | lVMV2 | lLIPd | lPOS1 | l33pr | ra24 |
| la9.46v | rMI | rRI | lp24pr | rLO2 | r52 | lMI | rPreS | rV3 | l3b |
| l9.46d | rPoI2 | r7Am | rp47r | r1 | ra24pr | rTE2a | rVIP | lFEF | l5L |
| lp9.46v | lIPS1 | rs6.8 | rVIP | r5L | lPFm | lPBelt | ld32 | rTPOJ2 | r10v |
| lp32pr | l5mv | r10d | rOFC | lTPOJ1 | lEC | rTPOJ1 | r7Am | r45 | lProS |
| r6r | ra32pr | ld32 | r10v | r6v | rPGp | rV7 | l43 | rPBelt | lMT |
| rPGp | rIPS1 | r31pd | rEC | lFEF | la24pr | lFOP3 | rPFt | r8Av | rV2 |
| lSTV | rTE2a | lPOS2 | l6d | rTE1p | rPOS2 | rTE1m | rPeEc | l31pd | rPHA2 |
| lA5 | lVVC | rFFC | lVIP | r44 | lMBelt | lTE1m | rDVT | rH | rPFop |
| lp10p | r43 | rPSL | l6r | lV4t | rPF | rPSL | rpOFC | lPCV | l3a |
| r23d | l7AL | r46 | l4 | lTE2p | rTGd | lPoI2 | r7AL | lV8 | l6d |
| rPF | rIFSa | lH | lAVI | rA1 | lSTSda | la10p | rTE2p | rV6A | r6a |
| lPreS | l52 | l31pv | rTGv | r4 | rV3B | lVVC | lPoI1 | lOP1 | rFOP3 |
| l23c | lPFcm | rOP2.3 | l25 | l3b | lPGi | lV6 | rFOP4 | lSTSvp | lV4t |
| lSTSda | lTPOJ3 | rRSC | la32pr | rAAIC | lTGv | rTE1a | lA4 | l55b | r3b |
| lV6 | l8Av | lPFt | l10v | r6mp | lPir | lTF | lMST | rTE1p | r6d |
| l43 | lMBelt | lOP1 | rAVI | la24 | rPoI2 | lPFop | r43 | rMT | rSFL |
| r7PL | rIP0 | lFOP1 | r24dv | lFFC | lp10p | l46 | r31a | r1 | lLO1 |
| ld23ab | rMIP | lSTSva | rPHA2 | l10pp | r6r | lv23ab | lIFJp | r6ma | l6mp |
| rPHT | lTE1m | ra10p | rLO1 | rV6A | l23c | rFOP1 | lOP2.3 | lFFC | rV4 |
| la24pr | lTE1p | rSTSdp | lV3 | l47l | li6.8 | l8Av | ra47r | ri6.8 | rPIT |
| lPGi | r23c | lOP4 | lV6A | l13l | rPFcm | lH | lPOS2 | rp47r | lLO3 |
| rIFJp | rIFSp | lPIT | lA1 | lOFC | l9a | rIP0 | lAAIC | rIFSa | lSFL |
| lDVT | lLIPv | lpOFC | lMST | rTPOJ2 | lPreS | r9m | l7m | lV1 | rFST |
| rPFm | lv23ab | l9p | rPH | l6v | lSTV | lSTSva | rp10p | r55b | rPH |
| lVMV2 | la47r | rA5 | lIFJp | rV8 | rIP1 | lPFcm | r8BM | rV1 | l25 |
| l7Am | rv23ab | lPEF | rH | rp24pr | r13l | rPCV | lSTSdp | r47m | rLO1 |
| lIP1 | r7m | r47s | lOP2.3 | lV4 | rPFm | lVIP | rV3CD | r31pd | r4 |
| lPHT | r7PC | r5mv | rDVT | rSFL | l45 | rIFJa | rLO3 | rTPOJ3 | lp32 |
| l8C | lFOP2 | lLBelt | rPFop | r24dd | l7Am | rSTSdp | rEC | lSCEF | rProS |
| rd23ab | rSTSda | rPir | lSTSdp | r25 | lTGd | r33pr | l6r | rAVI | rPHA3 |
| rSTV | rVVC | rPeEc | lSFL | l6mp | rSTV | l24dv | lA1 | r47s | rSCEF |
| r33pr | ra47r | lPI | lA4 | r10pp | ld23ab | rp32pr | l9p | l24dd | ls32 |
| lFOP4 | r9.46d | lProS | r6ma | lLO1 | lRSC | rPir | lIFJa | lMIP | l6ma |
| rPHA1 | lIFSa | lPBelt | rV4t | r47l | lIP1 | la9.46v | lV3CD | rVMV3 | r3a |
| rIP1 | r2 | la10p | l6ma | rOP4 | rMIP | l47m | rAAIC | ra9.46v | l8BL |
| r8C | lIg | ri6.8 | l55b | rFST | rPOS1 | lp47r | rA4 | r44 | lPHA2 |
| ls6.8 | li6.8 | lLO2 | r7AL | lLO3 | rMI | l7PL | lPGp | l7Pm | rp32 |
| lEC | l24dv | rV1 | lSCEF | l31pd | l44 | r10d | l9m | r10r | rp24pr |
| rTF | l8Ad | lPGp | l7Pm | rV4 | r9p | rRSC | rOP2.3 | lV6A | r5m |
| lLIPd | l44 | l47m | lFOP5 | lPHA1 | lIg | lFOP2 | lPH | rV3A | l6v |
| rPFcm | rTE1m | l9m | rSCEF | lV2 | rSTGa | lRI | rd32 | rV6 | rV8 |
| rV3B | rd32 | lPCV | ls32 | l3a | rSTSda | l5mv | l10v | rOFC | rV4t |
| rLO3 | lMI | rV6 | rFOP5 | lMIP | rVVC | lFOP5 | ls6.8 | r7Pm | lV2 |
| rPOS1 | lIFJa | rSTSva | lV3CD | rV3 | l8Ad | lPHT | rTGv | rVMV2 | l1 |
| rIFJa | lIP0 | l24dd | ra9.46v | rVMV3 | lIFSa | l11l | lVMV3 | lTE2a | r47l |
| lp24 | lRSC | rTPOJ1 | rMT | rLBelt | lFOP4 | r2 | rMST | l5m | lV4 |
| rV7 | rTA2 | rV3A | l2 | lPHA2 | r7PL | rIP2 | rp24 | rLBelt | rLO2 |
| rPCV | rMBelt | lV8 | r6a | r10r | rPHA1 | lTE1p | rs6.8 | l10pp | r24dd |
| lPFop | rPI | rPHA3 | rLIPv | ra24 | la47r | lFST | rFEF | lPHA1 | l4 |
| lAIP | r9p | r55b | rProS | lPHA3 | rTF | rFOP5 | lIPS1 | r5mv | lV3 |
| lp47r | l7PC | r11l | lMT | lp32 | rd23ab | lp24pr | l10d | r24dv | l10r |
| rMST | r31pv | rAIP | l5L | rp32 | rMBelt | l8BM | lTE2p | r10pp | la24 |

**S1.15 Ranked features for both the EBM and FBM feature selection methods for the Lpba40 FA model.**

| **EBM** | **FBM** |
| --- | --- |
| Right middle temporal gyrus | Left middle temporal gyrus |
| Left middle temporal gyrus | Right middle temporal gyrus |
| Right inferior temporal gyrus | Right caudate |
| Right caudate | Left inferior temporal gyrus |
| Left angular gyrus | Left hippocampus |
| Left middle orbitofrontal gyrus | Right insular cortex |
| Right hippocampus | Right inferior temporal gyrus |
| Left gyrus rectus | Right hippocampus |
| Right middle occipital gyrus | Left caudate |
| Left inferior temporal gyrus | Left gyrus rectus |
| Right cingulate gyrus | Left putamen |
| Right inferior frontal gyrus | Right superior temporal gyrus |
| Right insular cortex | Right cingulate gyrus |
| Right middle frontal gyrus | Right inferior frontal gyrus |
| Left caudate | brainstem |
| Left lateral orbitofrontal gyrus | Left lateral orbitofrontal gyrus |
| Left superior frontal gyrus | Left parahippocampal gyrus |
| Left hippocampus | Right gyrus rectus |
| Left insular cortex | Right middle frontal gyrus |
| Left putamen | Left middle orbitofrontal gyrus |
| Right fusiform gyrus | Left insular cortex |
| Right angular gyrus | Left middle frontal gyrus |
| brainstem | Left superior parietal gyrus |
| Left superior temporal gyrus | Right lateral orbitofrontal gyrus |
| Right superior temporal gyrus | cerebellum |
| Left middle frontal gyrus | Left cingulate gyrus |
| Left middle occipital gyrus | Right superior parietal gyrus |
| Right superior parietal gyrus | Left inferior occipital gyrus |
| Right postcentral gyrus | Left angular gyrus |
| Right lateral orbitofrontal gyrus | Left lingual gyrus |
| Left superior occipital gyrus | Right lingual gyrus |
| Right lingual gyrus | Right postcentral gyrus |
| Right precentral gyrus | Left superior temporal gyrus |
| cerebellum | Left fusiform gyrus |
| Left postcentral gyrus | Left middle occipital gyrus |
| Left inferior occipital gyrus | Left superior occipital gyrus |
| Right gyrus rectus | Left inferior frontal gyrus |
| Right middle orbitofrontal gyrus | Right parahippocampal gyrus |
| Left cingulate gyrus | Right supramarginal gyrus |
| Left parahippocampal gyrus | Right middle occipital gyrus |
| Left precuneus | Left superior frontal gyrus |
| Left precentral gyrus | Right cuneus |
| Left fusiform gyrus | Right angular gyrus |
| Right supramarginal gyrus | Right putamen |
| Left inferior frontal gyrus | Right precuneus |
| Left superior parietal gyrus | Left precentral gyrus |
| Right precuneus | Left cuneus |
| Right cuneus | Right middle orbitofrontal gyrus |
| Right superior occipital gyrus | Left postcentral gyrus |
| Right parahippocampal gyrus | Right fusiform gyrus |
| Left supramarginal gyrus | Left precuneus |
| Right superior frontal gyrus | Right superior occipital gyrus |
| Left lingual gyrus | Left supramarginal gyrus |
| Left cuneus | Right precentral gyrus |
| Right inferior occipital gyrus | Right inferior occipital gyrus |
| Right putamen | Right superior frontal gyrus |

**S1.16 Ranked features for both the EBM and FBM feature selection methods for the Lpba40 MD model.**

| **EBM** | **FBM** |
| --- | --- |
| Left middle temporal gyrus | Left lateral orbitofrontal gyrus |
| Left lateral orbitofrontal gyrus | Left middle temporal gyrus |
| Left hippocampus | Left inferior temporal gyrus |
| Left inferior frontal gyrus | Right cingulate gyrus |
| Left fusiform gyrus | Left inferior frontal gyrus |
| Left inferior temporal gyrus | Left hippocampus |
| Right cingulate gyrus | Left superior temporal gyrus |
| Right hippocampus | Left insular cortex |
| Left cingulate gyrus | Right hippocampus |
| Right insular cortex | Left fusiform gyrus |
| Left superior temporal gyrus | Left cingulate gyrus |
| Right caudate | Left inferior occipital gyrus |
| Left middle orbitofrontal gyrus | Right middle temporal gyrus |
| Left insular cortex | brainstem |
| Right inferior temporal gyrus | Left middle orbitofrontal gyrus |
| Left inferior occipital gyrus | Right middle orbitofrontal gyrus |
| brainstem | Right putamen |
| Right inferior frontal gyrus | Right caudate |
| Right fusiform gyrus | Right inferior temporal gyrus |
| Right angular gyrus | Left caudate |
| cerebellum | Left angular gyrus |
| Right putamen | Right superior temporal gyrus |
| Left superior frontal gyrus | Right insular cortex |
| Right middle temporal gyrus | Right inferior frontal gyrus |
| Left caudate | Right fusiform gyrus |
| Left middle occipital gyrus | Left supramarginal gyrus |
| Right middle occipital gyrus | Left middle occipital gyrus |
| Right middle orbitofrontal gyrus | Left parahippocampal gyrus |
| Right gyrus rectus | Left middle frontal gyrus |
| Left angular gyrus | Left lingual gyrus |
| Right superior temporal gyrus | Right lateral orbitofrontal gyrus |
| Right lingual gyrus | Left putamen |
| Left precentral gyrus | Right angular gyrus |
| Right superior frontal gyrus | Right middle frontal gyrus |
| Left superior occipital gyrus | Right inferior occipital gyrus |
| Right inferior occipital gyrus | Right middle occipital gyrus |
| Left cuneus | Right supramarginal gyrus |
| Right precentral gyrus | Left superior frontal gyrus |
| Left putamen | Right cuneus |
| Left superior parietal gyrus | cerebellum |
| Left supramarginal gyrus | Right precentral gyrus |
| Right superior occipital gyrus | Right precuneus |
| Right cuneus | Right gyrus rectus |
| Right parahippocampal gyrus | Left precuneus |
| Left precuneus | Left cuneus |
| Right postcentral gyrus | Right superior occipital gyrus |
| Right middle frontal gyrus | Left precentral gyrus |
| Left parahippocampal gyrus | Right postcentral gyrus |
| Left gyrus rectus | Left superior parietal gyrus |
| Left lingual gyrus | Right superior parietal gyrus |
| Right lateral orbitofrontal gyrus | Right superior frontal gyrus |
| Left postcentral gyrus | Right lingual gyrus |
| Right supramarginal gyrus | Left superior occipital gyrus |
| Right superior parietal gyrus | Left gyrus rectus |
| Left middle frontal gyrus | Right parahippocampal gyrus |
| Right precuneus | Left postcentral gyrus |

**S1.17 Ranked features for both the EBM and FBM feature selection methods for the Desikan FA model.**

| **EBM** | **FBM** |
| --- | --- |
| Inferior tempora lgyrus (left hemisphere) | Inferior tempora lgyrus (left hemisphere) |
| Pericalcarine cortex (left hemisphere) | Temporal pole (right hemisphere) |
| Entorhinal cortex (left hemisphere) | Middle temporal gyrus (left hemisphere) |
| Left-Hippocampus | Right-Caudate |
| Right-Thalamus-Proper | Left-Hippocampus |
| Middle temporal gyrus (right hemisphere) | Left-Amygdala |
| Insular cortex (right hemisphere) | Pericalcarine cortex (left hemisphere) |
| Right-Caudate | Right-Amygdala |
| Left-Amygdala | Middle temporal gyrus (right hemisphere) |
| Parstriangularis (left hemisphere) | Entorhinal cortex (left hemisphere) |
| Temporal pole (right hemisphere) | Insular cortex (right hemisphere) |
| Banks superior temporal sulcus (left hemisphere) | Caudal anterior-cingulate cortex (right hemisphere) |
| Middle temporal gyrus (left hemisphere) | Right-Cerebellum-Cortex |
| Postcentral gyrus (left hemisphere) | Left-Thalamus-Proper |
| Rostral anterior cingulate cortex (left hemisphere) | Temporal pole (left hemisphere) |
| Right-Cerebellum-Cortex | Parahippocampal gyrus (right hemisphere) |
| Left-Caudate | Medial orbital frontal cortex (right hemisphere) |
| Left-Putamen | Supramarginal gyrus (right hemisphere) |
| Left-Accumbens-area | Parstriangularis (left hemisphere) |
| Inferior parietal cortex (left hemisphere) | Inferior parietal cortex (left hemisphere) |
| Banks superior temporal sulcus (right hemisphere) | Right-Thalamus-Proper |
| Right-Amygdala | Banks superior temporal sulcus (left hemisphere) |
| Lateral occipital cortex (left hemisphere) | Left-Putamen |
| Supramarginal gyrus (left hemisphere) | Left-Caudate |
| Parahippocampal gyrus (right hemisphere) | Insular cortex (left hemisphere) |
| Insular cortex (left hemisphere) | Inferior temporal gyrus (right hemisphere) |
| Caudal anterior-cingulate cortex (left hemisphere) | Parstriangularis (right hemisphere) |
| Inferior temporal gyrus (right hemisphere) | Caudal middle frontal gyrus (left hemisphere) |
| Medial orbital frontal cortex (right hemisphere) | Superior temporal gyrus (right hemisphere) |
| Left-Thalamus-Proper | Transverse temporal cortex (right hemisphere) |
| Transverse temporal cortex (right hemisphere) | Lateral occipital cortex (left hemisphere) |
| Parstriangularis (right hemisphere) | Right-Hippocampus |
| Parsopercularis (right hemisphere) | Lingual gyrus (left hemisphere) |
| Right-Pallidum | Pericalcarine cortex (right hemisphere) |
| Caudal middle frontal gyrus (left hemisphere) | Isthmus-cingulate cortex (left hemisphere) |
| Temporal pole (left hemisphere) | Lateral orbital frontal cortex (left hemisphere) |
| Caudal anterior-cingulate cortex (right hemisphere) | Rostral middle frontal gyrus (right hemisphere) |
| Rostral middle frontal gyrus (right hemisphere) | Posterior-cingulate cortex (left hemisphere) |
| Supramarginal gyrus (right hemisphere) | Postcentral gyrus (right hemisphere) |
| Right-Hippocampus | Parahippocampal gyrus (left hemisphere) |
| Frontal pole (left hemisphere) | Rostral middle frontal gyrus (left hemisphere) |
| Frontal pole (right hemisphere) | Frontal pole (right hemisphere) |
| Posterior-cingulate cortex (left hemisphere) | Rostral anterior cingulate cortex (left hemisphere) |
| Lingual gyrus (right hemisphere) | Frontal pole (left hemisphere) |
| Parsorbitalis (right hemisphere) | Inferior parietal cortex (right hemisphere) |
| Pericalcarine cortex (right hemisphere) | Left-Cerebellum-Cortex |
| Isthmus-cingulate cortex (left hemisphere) | Rostral anterior cingulate cortex (right hemisphere) |
| Superior temporal gyrus (right hemisphere) | Parsopercularis (right hemisphere) |
| Caudal middle frontal gyrus (right hemisphere) | Superior parietal cortex (right hemisphere) |
| Lingual gyrus (left hemisphere) | Precentral gyrus (right hemisphere) |
| Superior parietal cortex (right hemisphere) | Medial orbital frontal cortex (left hemisphere) |
| Postcentral gyrus (right hemisphere) | Lateral orbital frontal cortex (right hemisphere) |
| Medial orbital frontal cortex (left hemisphere) | Entorhinal cortex (right hemisphere) |
| Inferior parietal cortex (right hemisphere) | Cuneus cortex (right hemisphere) |
| Rostral anterior cingulate cortex (right hemisphere) | Caudal middle frontal gyrus (right hemisphere) |
| Lateral orbital frontal cortex (left hemisphere) | Fusiform gyrus (right hemisphere) |
| Superior temporal gyrus (left hemisphere) | Superior parietal cortex (left hemisphere) |
| Precuneus cortex (right hemisphere) | Banks superior temporal sulcus (right hemisphere) |
| Left-Cerebellum-Cortex | Cuneus cortex (left hemisphere) |
| Lateral orbital frontal cortex (right hemisphere) | Fusiform gyrus (left hemisphere) |
| Parahippocampal gyrus (left hemisphere) | Paracentral lobule (right hemisphere) |
| Parsorbitalis (left hemisphere) | Supramarginal gyrus (left hemisphere) |
| Superior frontal gyrus (left hemisphere) | Superior frontal gyrus (left hemisphere) |
| Precentral gyrus (right hemisphere) | Caudal anterior-cingulate cortex (left hemisphere) |
| Superior parietal cortex (left hemisphere) | Superior frontal gyrus (right hemisphere) |
| Precentral gyrus (left hemisphere) | Transverse temporal cortex (left hemisphere) |
| Lateral occipital cortex (right hemisphere) | Paracentral lobule (left hemisphere) |
| Posterior-cingulate cortex (right hemisphere) | Postcentral gyrus (left hemisphere) |
| Fusiform gyrus (right hemisphere) | Left-Accumbens-area |
| Paracentral lobule (left hemisphere) | Precuneus cortex (right hemisphere) |
| Superior frontal gyrus (right hemisphere) | Posterior-cingulate cortex (right hemisphere) |
| Right-Accumbens-area | Precentral gyrus (left hemisphere) |
| Entorhinal cortex (right hemisphere) | Right-Putamen |
| Rostral middle frontal gyrus (left hemisphere) | Parsorbitalis (left hemisphere) |
| Left-Pallidum | Parsorbitalis (right hemisphere) |
| Isthmus-cingulate cortex (right hemisphere) | Lingual gyrus (right hemisphere) |
| Transverse temporal cortex (left hemisphere) | Precuneus cortex (left hemisphere) |
| Paracentral lobule (right hemisphere) | Superior temporal gyrus (left hemisphere) |
| Cuneus cortex (right hemisphere) | Lateral occipital cortex (right hemisphere) |
| Parsopercularis (left hemisphere) | Right-Accumbens-area |
| Cuneus cortex (left hemisphere) | Parsopercularis (left hemisphere) |
| Precuneus cortex (left hemisphere) | Left-Pallidum |
| Fusiform gyrus (left hemisphere) | Right-Pallidum |
| Right-Putamen | Isthmus-cingulate cortex (right hemisphere) |

**S1.18 Ranked features for both the EBM and FBM feature selection methods for the Desikan MD model.**

| **EBM** | **FBM** |
| --- | --- |
| Left-Hippocampus | Left-Amygdala |
| Left-Amygdala | Middle temporal gyrus (left hemisphere) |
| Middle temporal gyrus (left hemisphere) | Parsorbitalis (left hemisphere) |
| Transverse temporal cortex (right hemisphere) | Banks superior temporal sulcus (left hemisphere) |
| Banks superior temporal sulcus (left hemisphere) | Inferior tempora lgyrus (left hemisphere) |
| Entorhinal cortex (left hemisphere) | Lateral orbital frontal cortex (left hemisphere) |
| Parsorbitalis (left hemisphere) | Parstriangularis (left hemisphere) |
| Right-Amygdala | Entorhinal cortex (left hemisphere) |
| Left-Accumbens-area | Caudal anterior-cingulate cortex (right hemisphere) |
| Inferior tempora lgyrus (left hemisphere) | Pericalcarine cortex (left hemisphere) |
| Caudal anterior-cingulate cortex (right hemisphere) | Middle temporal gyrus (right hemisphere) |
| Parstriangularis (left hemisphere) | Left-Hippocampus |
| Lateral orbital frontal cortex (left hemisphere) | Transverse temporal cortex (right hemisphere) |
| Pericalcarine cortex (left hemisphere) | Rostral anterior cingulate cortex (right hemisphere) |
| Middle temporal gyrus (right hemisphere) | Superior temporal gyrus (left hemisphere) |
| Supramarginal gyrus (right hemisphere) | Rostral middle frontal gyrus (left hemisphere) |
| Transverse temporal cortex (left hemisphere) | Right-Hippocampus |
| Cuneus cortex (right hemisphere) | Transverse temporal cortex (left hemisphere) |
| Pericalcarine cortex (right hemisphere) | Insular cortex (left hemisphere) |
| Right-Hippocampus | Right-Amygdala |
| Right-Thalamus-Proper | Inferior parietal cortex (left hemisphere) |
| Superior temporal gyrus (left hemisphere) | Pericalcarine cortex (right hemisphere) |
| Insular cortex (left hemisphere) | Rostral anterior cingulate cortex (left hemisphere) |
| Inferior parietal cortex (left hemisphere) | Lateral occipital cortex (left hemisphere) |
| Right-Caudate | Parahippocampal gyrus (left hemisphere) |
| Parsopercularis (right hemisphere) | Supramarginal gyrus (right hemisphere) |
| Medial orbital frontal cortex (left hemisphere) | Right-Putamen |
| Left-Caudate | Left-Accumbens-area |
| Parsopercularis (left hemisphere) | Right-Caudate |
| Rostral middle frontal gyrus (left hemisphere) | Superior temporal gyrus (right hemisphere) |
| Parsorbitalis (right hemisphere) | Caudal anterior-cingulate cortex (left hemisphere) |
| Left-Thalamus-Proper | Parsopercularis (left hemisphere) |
| Rostral anterior cingulate cortex (right hemisphere) | Banks superior temporal sulcus (right hemisphere) |
| Lateral occipital cortex (left hemisphere) | Cuneus cortex (right hemisphere) |
| Fusiform gyrus (left hemisphere) | Medial orbital frontal cortex (left hemisphere) |
| Right-Putamen | Fusiform gyrus (left hemisphere) |
| Inferior parietal cortex (right hemisphere) | Rostral middle frontal gyrus (right hemisphere) |
| Fusiform gyrus (right hemisphere) | Right-Thalamus-Proper |
| Caudal anterior-cingulate cortex (left hemisphere) | Left-Thalamus-Proper |
| Left-Pallidum | Parsopercularis (right hemisphere) |
| Superior temporal gyrus (right hemisphere) | Isthmus-cingulate cortex (right hemisphere) |
| Rostral anterior cingulate cortex (left hemisphere) | Insular cortex (right hemisphere) |
| Rostral middle frontal gyrus (right hemisphere) | Left-Caudate |
| Isthmus-cingulate cortex (right hemisphere) | Supramarginal gyrus (left hemisphere) |
| Supramarginal gyrus (left hemisphere) | Inferior temporal gyrus (right hemisphere) |
| Banks superior temporal sulcus (right hemisphere) | Entorhinal cortex (right hemisphere) |
| Right-Cerebellum-Cortex | Medial orbital frontal cortex (right hemisphere) |
| Parahippocampal gyrus (left hemisphere) | Lingual gyrus (right hemisphere) |
| Left-Putamen | Posterior-cingulate cortex (right hemisphere) |
| Medial orbital frontal cortex (right hemisphere) | Right-Accumbens-area |
| Right-Accumbens-area | Lateral orbital frontal cortex (right hemisphere) |
| Caudal middle frontal gyrus (left hemisphere) | Left-Putamen |
| Frontal pole (left hemisphere) | Inferior parietal cortex (right hemisphere) |
| Cuneus cortex (left hemisphere) | Parstriangularis (right hemisphere) |
| Frontal pole (right hemisphere) | Parahippocampal gyrus (right hemisphere) |
| Insular cortex (right hemisphere) | Precuneus cortex (left hemisphere) |
| Lateral orbital frontal cortex (right hemisphere) | Paracentral lobule (left hemisphere) |
| Paracentral lobule (left hemisphere) | Fusiform gyrus (right hemisphere) |
| Entorhinal cortex (right hemisphere) | Left-Pallidum |
| Parstriangularis (right hemisphere) | Frontal pole (left hemisphere) |
| Posterior-cingulate cortex (right hemisphere) | Superior frontal gyrus (right hemisphere) |
| Precuneus cortex (left hemisphere) | Caudal middle frontal gyrus (left hemisphere) |
| Precuneus cortex (right hemisphere) | Precuneus cortex (right hemisphere) |
| Lateral occipital cortex (right hemisphere) | Temporal pole (right hemisphere) |
| Inferior temporal gyrus (right hemisphere) | Right-Pallidum |
| Lingual gyrus (left hemisphere) | Parsorbitalis (right hemisphere) |
| Precentral gyrus (left hemisphere) | Isthmus-cingulate cortex (left hemisphere) |
| Lingual gyrus (right hemisphere) | Superior frontal gyrus (left hemisphere) |
| Left-Cerebellum-Cortex | Lateral occipital cortex (right hemisphere) |
| Precentral gyrus (right hemisphere) | Postcentral gyrus (left hemisphere) |
| Posterior-cingulate cortex (left hemisphere) | Cuneus cortex (left hemisphere) |
| Superior frontal gyrus (left hemisphere) | Posterior-cingulate cortex (left hemisphere) |
| Caudal middle frontal gyrus (right hemisphere) | Right-Cerebellum-Cortex |
| Temporal pole (right hemisphere) | Lingual gyrus (left hemisphere) |
| Parahippocampal gyrus (right hemisphere) | Frontal pole (right hemisphere) |
| Postcentral gyrus (left hemisphere) | Paracentral lobule (right hemisphere) |
| Paracentral lobule (right hemisphere) | Caudal middle frontal gyrus (right hemisphere) |
| Superior parietal cortex (right hemisphere) | Temporal pole (left hemisphere) |
| Isthmus-cingulate cortex (left hemisphere) | Superior parietal cortex (left hemisphere) |
| Superior frontal gyrus (right hemisphere) | Precentral gyrus (right hemisphere) |
| Superior parietal cortex (left hemisphere) | Left-Cerebellum-Cortex |
| Postcentral gyrus (right hemisphere) | Superior parietal cortex (right hemisphere) |
| Right-Pallidum | Precentral gyrus (left hemisphere) |
| Temporal pole (left hemisphere) | Postcentral gyrus (right hemisphere) |

**S1.19 Ranked features for both the EBM and FBM feature selection methods for the Destrieux FA model.**

| **EBM** | **FBM** |
| --- | --- |
| ctx_lh_S_circular_insula_sup | ctx_lh_G_temporal_inf |
| ctx_rh_G_temporal_middle | ctx_rh_G_temporal_middle |
| ctx_rh_S_circular_insula_sup | ctx_rh_S_circular_insula_sup |
| Left.Hippocampus | ctx_lh_S_temporal_sup |
| ctx_rh_S_pericallosal | ctx_rh_G_temporal_inf |
| Right.Thalamus.Proper | ctx_lh_S_circular_insula_sup |
| ctx_lh_S_occipital_ant | Right.Caudate |
| ctx_rh_G_temporal_inf | Left.Amygdala |
| Left.Amygdala | Left.Hippocampus |
| Right.Caudate | Right.Amygdala |
| ctx_lh_G_subcallosal | ctx_lh_S_oc.temp_med_and_Lingual |
| ctx_lh_G_temporal_inf | ctx_lh_G_oc.temp_med.Parahip |
| ctx_rh_G_front_middle | ctx_lh_S_front_middle |
| ctx_lh_G_and_S_occipital_inf | ctx_lh_S_occipital_ant |
| ctx_rh_S_front_sup | ctx_rh_S_pericallosal |
| ctx_lh_S_temporal_sup | ctx_lh_G_temporal_middle |
| ctx_rh_G_postcentral | ctx_lh_G_and_S_occipital_inf |
| ctx_rh_S_circular_insula_inf | ctx_lh_G_subcallosal |
| ctx_lh_G_cingul.Post.ventral | Right.Cerebellum.Cortex |
| ctx_lh_S_pericallosal | ctx_lh_S_postcentral |
| ctx_rh_S_front_middle | Left.Thalamus.Proper |
| ctx_rh_G_rectus | ctx_rh_G_oc.temp_med.Parahip |
| ctx_lh_G_temporal_middle | ctx_rh_G_temp_sup.G_T_transv |
| ctx_lh_G_oc.temp_med.Parahip | ctx_rh_S_temporal_sup |
| Left.Putamen | ctx_rh_S_collat_transv_post |
| Right.Cerebellum.Cortex | ctx_lh_Lat_Fis.ant.Vertical |
| Left.Caudate | ctx_lh_S_calcarine |
| ctx_lh_G_rectus | Right.Thalamus.Proper |
| ctx_rh_S_collat_transv_post | ctx_rh_G_front_inf.Opercular |
| ctx_lh_S_postcentral | ctx_rh_S_occipital_ant |
| ctx_rh_S_front_inf | ctx_rh_Lat_Fis.post |
| Left.Accumbens.area | ctx_rh_S_collat_transv_ant |
| ctx_rh_G_oc.temp_med.Parahip | Left.Putamen |
| ctx_rh_S_temporal_sup | Left.Caudate |
| ctx_rh_G_pariet_inf.Angular | ctx_lh_S_orbital.H_Shaped |
| Right.Amygdala | ctx_rh_Lat_Fis.ant.Horizont |
| ctx_rh_Pole_temporal | ctx_rh_G_pariet_inf.Supramar |
| ctx_lh_S_oc.temp_lat | ctx_lh_G_rectus |
| ctx_lh_S_orbital_lateral | ctx_lh_G_cingul.Post.ventral |
| ctx_lh_S_front_middle | ctx_lh_S_temporal_transverse |
| ctx_lh_S_oc.temp_med_and_Lingual | ctx_rh_S_postcentral |
| ctx_rh_G_Ins_lg_and_S_cent_ins | ctx_rh_S_front_sup |
| ctx_lh_S_interm_prim.Jensen | ctx_rh_S_front_inf |
| ctx_rh_Lat_Fis.post | Right.Hippocampus |
| ctx_rh_S_temporal_inf | ctx_rh_G_Ins_lg_and_S_cent_ins |
| ctx_rh_Lat_Fis.ant.Horizont | ctx_rh_G_temp_sup.Plan_polar |
| Left.Thalamus.Proper | ctx_lh_G_pariet_inf.Angular |
| ctx_lh_G_Ins_lg_and_S_cent_ins | ctx_rh_Lat_Fis.ant.Vertical |
| ctx_rh_G_insular_short | ctx_lh_S_circular_insula_ant |
| ctx_rh_S_collat_transv_ant | ctx_rh_S_precentral.inf.part |
| ctx_rh_G_temp_sup.Plan_polar | ctx_rh_G_front_middle |
| Right.Pallidum | ctx_lh_S_suborbital |
| ctx_lh_S_calcarine | ctx_rh_S_temporal_transverse |
| ctx_rh_S_occipital_ant | ctx_lh_G_Ins_lg_and_S_cent_ins |
| ctx_lh_G_pariet_inf.Angular | ctx_rh_Pole_temporal |
| ctx_lh_S_temporal_inf | ctx_lh_G_cingul.Post.dorsal |
| ctx_rh_S_oc.temp_lat | ctx_lh_S_oc.temp_lat |
| ctx_rh_G_precuneus | ctx_rh_S_temporal_inf |
| ctx_rh_G_pariet_inf.Supramar | ctx_lh_S_cingul.Marginalis |
| ctx_rh_G_front_inf.Opercular | ctx_lh_Pole_occipital |
| ctx_rh_G_cuneus | ctx_lh_G_temp_sup.G_T_transv |
| ctx_lh_S_orbital.H_Shaped | ctx_rh_S_orbital_med.olfact |
| ctx_lh_S_cingul.Marginalis | ctx_lh_G_occipital_sup |
| ctx_lh_S_circular_insula_ant | ctx_rh_G_postcentral |
| ctx_lh_S_front_inf | ctx_rh_G_insular_short |
| Right.Hippocampus | ctx_lh_S_front_inf |
| ctx_lh_S_oc_sup_and_transversal | ctx_rh_G_subcallosal |
| ctx_lh_G_temp_sup.G_T_transv | ctx_rh_G_precentral |
| ctx_lh_G_pariet_inf.Supramar | ctx_lh_G_insular_short |
| ctx_lh_S_oc_middle_and_Lunatus | ctx_rh_G_cuneus |
| ctx_lh_G_postcentral | ctx_rh_S_orbital.H_Shaped |
| ctx_rh_G_temp_sup.G_T_transv | ctx_lh_S_pericallosal |
| ctx_rh_S_temporal_transverse | Left.Cerebellum.Cortex |
| ctx_rh_S_subparietal | ctx_lh_S_central |
| ctx_lh_Lat_Fis.ant.Vertical | ctx_rh_G_temp_sup.Plan_tempo |
| ctx_rh_G_front_inf.Triangul | ctx_rh_S_front_middle |
| ctx_lh_Pole_occipital | ctx_lh_S_temporal_inf |
| ctx_rh_S_orbital_med.olfact | ctx_lh_S_collat_transv_ant |
| ctx_rh_S_precentral.inf.part | ctx_lh_G_parietal_sup |
| ctx_rh_Lat_Fis.ant.Vertical | ctx_lh_Lat_Fis.ant.Horizont |
| ctx_lh_G_and_S_subcentral | ctx_rh_G_parietal_sup |
| ctx_lh_S_orbital_med.olfact | ctx_lh_S_precentral.sup.part |
| ctx_lh_G_insular_short | ctx_lh_G_and_S_subcentral |
| ctx_lh_G_temp_sup.Plan_polar | ctx_lh_G_front_middle |
| ctx_lh_G_occipital_middle | ctx_lh_S_oc_middle_and_Lunatus |
| ctx_rh_S_intrapariet_and_P_tran | ctx_rh_G_front_inf.Triangul |
| ctx_lh_G_occipital_sup | ctx_lh_G_front_inf.Orbital |
| ctx_rh_G_precentral | ctx_rh_S_oc_sup_and_transversal |
| ctx_lh_S_central | ctx_rh_S_intrapariet_and_P_tran |
| ctx_rh_S_oc_sup_and_transversal | ctx_rh_G_rectus |
| ctx_lh_G_orbital | ctx_lh_G_and_S_transv_frontopol |
| ctx_rh_S_central | ctx_lh_G_temp_sup.Plan_tempo |
| ctx_lh_G_and_S_frontomargin | ctx_lh_G_cuneus |
| Left.Cerebellum.Cortex | ctx_rh_S_oc.temp_lat |
| ctx_rh_G_oc.temp_lat.fusifor | ctx_rh_S_oc.temp_med_and_Lingual |
| ctx_rh_G_front_inf.Orbital | ctx_lh_S_interm_prim.Jensen |
| ctx_rh_G_and_S_cingul.Mid.Ant | ctx_lh_Lat_Fis.post |
| ctx_lh_S_front_sup | ctx_lh_G_and_S_cingul.Mid.Ant |
| ctx_lh_S_suborbital | ctx_rh_G_pariet_inf.Angular |
| ctx_rh_G_and_S_transv_frontopol | ctx_lh_G_and_S_cingul.Ant |
| ctx_lh_G_front_inf.Orbital | ctx_lh_S_orbital_med.olfact |
| ctx_lh_G_and_S_transv_frontopol | ctx_lh_G_temp_sup.Lateral |
| ctx_rh_G_occipital_sup | ctx_rh_G_and_S_occipital_inf |
| ctx_rh_S_calcarine | ctx_lh_G_oc.temp_lat.fusifor |
| ctx_rh_S_orbital.H_Shaped | ctx_lh_G_temp_sup.Plan_polar |
| ctx_rh_G_front_sup | ctx_rh_G_and_S_subcentral |
| ctx_rh_S_oc.temp_med_and_Lingual | ctx_rh_S_orbital_lateral |
| ctx_lh_G_front_middle | ctx_lh_G_front_inf.Opercular |
| ctx_rh_S_precentral.sup.part | ctx_rh_G_oc.temp_lat.fusifor |
| ctx_lh_S_intrapariet_and_P_tran | ctx_lh_Pole_temporal |
| ctx_rh_S_circular_insula_ant | ctx_rh_G_and_S_frontomargin |
| ctx_lh_G_and_S_cingul.Mid.Ant | ctx_rh_G_orbital |
| ctx_rh_S_suborbital | ctx_rh_G_temp_sup.Lateral |
| ctx_rh_G_temp_sup.Lateral | ctx_rh_G_precuneus |
| ctx_lh_G_parietal_sup | ctx_lh_S_intrapariet_and_P_tran |
| ctx_lh_S_precentral.inf.part | ctx_lh_G_occipital_middle |
| ctx_lh_Lat_Fis.post | ctx_rh_S_calcarine |
| ctx_rh_G_and_S_frontomargin | ctx_rh_S_oc_middle_and_Lunatus |
| ctx_lh_G_and_S_cingul.Ant | ctx_lh_G_and_S_paracentral |
| ctx_rh_Pole_occipital | ctx_rh_G_and_S_paracentral |
| Left.Pallidum | ctx_rh_S_precentral.sup.part |
| ctx_rh_G_and_S_cingul.Mid.Post | ctx_lh_S_orbital_lateral |
| ctx_lh_G_front_inf.Triangul | ctx_rh_G_oc.temp_med.Lingual |
| Right.Accumbens.area | ctx_lh_G_postcentral |
| ctx_lh_G_precuneus | ctx_rh_G_and_S_transv_frontopol |
| ctx_lh_G_temp_sup.Lateral | ctx_lh_G_and_S_frontomargin |
| ctx_lh_G_front_sup | ctx_lh_G_pariet_inf.Supramar |
| ctx_rh_S_interm_prim.Jensen | Left.Accumbens.area |
| ctx_rh_G_cingul.Post.dorsal | ctx_rh_G_cingul.Post.ventral |
| ctx_lh_G_cingul.Post.dorsal | ctx_rh_G_cingul.Post.dorsal |
| ctx_rh_G_occipital_middle | ctx_rh_S_interm_prim.Jensen |
| ctx_lh_G_cuneus | ctx_rh_S_circular_insula_inf |
| ctx_lh_G_and_S_paracentral | ctx_rh_S_subparietal |
| ctx_lh_S_circular_insula_inf | ctx_lh_S_subparietal |
| ctx_rh_G_subcallosal | ctx_lh_G_and_S_cingul.Mid.Post |
| ctx_lh_S_temporal_transverse | Right.Putamen |
| ctx_rh_G_oc.temp_med.Lingual | ctx_lh_G_orbital |
| ctx_lh_G_temp_sup.Plan_tempo | ctx_rh_G_and_S_cingul.Mid.Ant |
| ctx_rh_S_orbital_lateral | ctx_lh_S_front_sup |
| ctx_lh_S_collat_transv_ant | ctx_lh_S_circular_insula_inf |
| ctx_lh_S_precentral.sup.part | ctx_rh_G_occipital_sup |
| ctx_rh_G_orbital | ctx_lh_G_front_sup |
| ctx_lh_Pole_temporal | ctx_rh_S_central |
| ctx_rh_S_parieto_occipital | ctx_rh_Pole_occipital |
| ctx_lh_G_oc.temp_lat.fusifor | Right.Accumbens.area |
| ctx_rh_G_cingul.Post.ventral | ctx_lh_S_precentral.inf.part |
| ctx_rh_G_parietal_sup | ctx_rh_S_circular_insula_ant |
| ctx_rh_G_temp_sup.Plan_tempo | ctx_lh_S_parieto_occipital |
| ctx_rh_G_and_S_paracentral | ctx_lh_G_precentral |
| ctx_lh_S_collat_transv_post | ctx_lh_S_oc_sup_and_transversal |
| ctx_lh_G_front_inf.Opercular | ctx_lh_G_precuneus |
| ctx_lh_S_subparietal | ctx_rh_G_and_S_cingul.Mid.Post |
| ctx_rh_G_and_S_subcentral | ctx_rh_S_cingul.Marginalis |
| ctx_lh_Lat_Fis.ant.Horizont | Left.Pallidum |
| ctx_rh_G_and_S_cingul.Ant | ctx_lh_G_front_inf.Triangul |
| ctx_rh_S_postcentral | ctx_rh_S_suborbital |
| ctx_rh_G_and_S_occipital_inf | ctx_rh_G_and_S_cingul.Ant |
| ctx_lh_G_precentral | ctx_rh_S_parieto_occipital |
| ctx_lh_G_oc.temp_med.Lingual | ctx_rh_G_front_sup |
| ctx_lh_G_and_S_cingul.Mid.Post | Right.Pallidum |
| ctx_lh_S_parieto_occipital | ctx_lh_G_oc.temp_med.Lingual |
| ctx_rh_S_oc_middle_and_Lunatus | ctx_rh_G_front_inf.Orbital |
| ctx_rh_S_cingul.Marginalis | ctx_lh_S_collat_transv_post |
| Right.Putamen | ctx_rh_G_occipital_middle |

**S1.20 Ranked features for both the EBM and FBM feature selection methods for the Destrieux MD model.**

| **EBM** | **FBM** |
| --- | --- |
| Left.Hippocampus | Left.Amygdala |
| Left.Amygdala | ctx_lh_S_temporal_sup |
| ctx_lh_S_circular_insula_sup | ctx_lh_S_orbital.H_Shaped |
| ctx_lh_G_temporal_middle | ctx_lh_G_oc.temp_med.Parahip |
| ctx_rh_S_circular_insula_sup | ctx_lh_G_temporal_middle |
| ctx_lh_S_front_middle | ctx_lh_S_front_middle |
| ctx_lh_S_orbital_lateral | ctx_rh_G_and_S_cingul.Ant |
| ctx_lh_S_orbital.H_Shaped | ctx_lh_G_front_inf.Orbital |
| ctx_lh_S_temporal_sup | ctx_lh_S_circular_insula_sup |
| ctx_lh_G_front_inf.Orbital | ctx_lh_S_orbital_lateral |
| ctx_rh_G_temp_sup.G_T_transv | ctx_rh_G_temp_sup.G_T_transv |
| Right.Amygdala | ctx_lh_G_temporal_inf |
| Left.Accumbens.area | ctx_rh_Lat_Fis.post |
| ctx_rh_Lat_Fis.post | ctx_lh_G_and_S_cingul.Ant |
| ctx_lh_S_front_inf | ctx_lh_S_suborbital |
| ctx_lh_G_oc.temp_med.Parahip | ctx_rh_S_circular_insula_sup |
| ctx_rh_G_and_S_cingul.Ant | ctx_rh_S_temporal_inf |
| ctx_rh_G_cuneus | ctx_rh_S_temporal_sup |
| ctx_lh_S_suborbital | ctx_lh_S_occipital_ant |
| ctx_rh_S_calcarine | Left.Hippocampus |
| ctx_lh_Lat_Fis.ant.Horizont | ctx_rh_G_temporal_middle |
| ctx_rh_S_temporal_inf | ctx_rh_S_suborbital |
| ctx_lh_G_temporal_inf | ctx_rh_G_and_S_cingul.Mid.Ant |
| ctx_rh_G_temporal_middle | ctx_lh_G_orbital |
| ctx_lh_S_oc.temp_lat | ctx_rh_S_calcarine |
| ctx_lh_G_and_S_cingul.Ant | ctx_lh_G_front_inf.Triangul |
| ctx_lh_S_occipital_ant | ctx_lh_S_subparietal |
| ctx_rh_S_occipital_ant | ctx_rh_S_orbital.H_Shaped |
| ctx_rh_G_temp_sup.Plan_tempo | ctx_lh_S_circular_insula_inf |
| ctx_lh_S_circular_insula_inf | Right.Hippocampus |
| ctx_lh_S_circular_insula_ant | ctx_lh_G_and_S_subcentral |
| ctx_rh_S_oc.temp_med_and_Lingual | ctx_rh_Lat_Fis.ant.Horizont |
| Right.Hippocampus | ctx_lh_S_oc.temp_lat |
| ctx_lh_G_oc.temp_lat.fusifor | ctx_lh_S_temporal_inf |
| ctx_lh_S_collat_transv_ant | ctx_lh_G_and_S_occipital_inf |
| ctx_rh_S_front_inf | ctx_rh_G_cuneus |
| ctx_rh_G_and_S_frontomargin | Right.Amygdala |
| ctx_lh_S_temporal_inf | ctx_lh_G_temp_sup.G_T_transv |
| ctx_lh_S_calcarine | ctx_lh_S_front_inf |
| ctx_lh_Lat_Fis.post | ctx_lh_S_circular_insula_ant |
| ctx_lh_G_temp_sup.G_T_transv | ctx_lh_Lat_Fis.post |
| ctx_rh_G_and_S_cingul.Mid.Ant | ctx_rh_G_temp_sup.Plan_tempo |
| Right.Thalamus.Proper | ctx_lh_G_temp_sup.Plan_tempo |
| ctx_rh_S_suborbital | ctx_rh_S_circular_insula_inf |
| ctx_rh_S_front_middle | ctx_lh_Lat_Fis.ant.Horizont |
| ctx_rh_S_temporal_sup | ctx_rh_S_front_inf |
| ctx_lh_G_orbital | ctx_rh_S_front_middle |
| ctx_rh_S_orbital.H_Shaped | ctx_lh_G_front_inf.Opercular |
| ctx_rh_G_temp_sup.Plan_polar | ctx_rh_G_temp_sup.Plan_polar |
| ctx_rh_G_oc.temp_med.Parahip | Right.Putamen |
| ctx_lh_G_and_S_occipital_inf | ctx_lh_G_and_S_transv_frontopol |
| ctx_rh_Lat_Fis.ant.Horizont | ctx_rh_S_temporal_transverse |
| ctx_lh_G_cingul.Post.ventral | Left.Accumbens.area |
| ctx_lh_G_and_S_subcentral | Right.Caudate |
| ctx_rh_G_pariet_inf.Supramar | ctx_lh_G_and_S_frontomargin |
| ctx_rh_S_oc.temp_lat | ctx_rh_G_oc.temp_med.Parahip |
| ctx_rh_S_intrapariet_and_P_tran | ctx_lh_S_parieto_occipital |
| ctx_rh_G_front_inf.Opercular | ctx_rh_S_front_sup |
| Right.Caudate | ctx_lh_G_and_S_cingul.Mid.Ant |
| ctx_lh_G_temp_sup.Plan_tempo | ctx_lh_S_collat_transv_ant |
| ctx_lh_G_front_inf.Opercular | ctx_lh_G_temp_sup.Plan_polar |
| ctx_lh_S_subparietal | ctx_lh_G_subcallosal |
| ctx_lh_S_front_sup | ctx_lh_G_Ins_lg_and_S_cent_ins |
| Left.Caudate | ctx_lh_S_front_sup |
| ctx_lh_G_front_inf.Triangul | Right.Thalamus.Proper |
| Left.Thalamus.Proper | ctx_lh_G_oc.temp_lat.fusifor |
| ctx_rh_S_postcentral | Left.Thalamus.Proper |
| ctx_lh_G_and_S_frontomargin | ctx_lh_S_postcentral |
| Right.Putamen | ctx_lh_G_occipital_middle |
| ctx_lh_G_temp_sup.Plan_polar | ctx_rh_G_and_S_transv_frontopol |
| ctx_lh_G_subcallosal | ctx_rh_S_oc.temp_med_and_Lingual |
| ctx_lh_G_and_S_transv_frontopol | ctx_rh_S_occipital_ant |
| Left.Pallidum | ctx_rh_G_cingul.Post.dorsal |
| ctx_lh_G_pariet_inf.Angular | ctx_lh_S_calcarine |
| ctx_rh_S_circular_insula_inf | ctx_lh_G_temp_sup.Lateral |
| ctx_lh_S_oc.temp_med_and_Lingual | ctx_rh_S_oc.temp_lat |
| ctx_rh_S_orbital_med.olfact | Left.Caudate |
| ctx_rh_S_temporal_transverse | ctx_rh_G_temporal_inf |
| ctx_lh_S_parieto_occipital | ctx_rh_S_orbital_med.olfact |
| ctx_rh_S_front_sup | ctx_lh_Lat_Fis.ant.Vertical |
| ctx_lh_G_pariet_inf.Supramar | ctx_rh_G_front_inf.Opercular |
| ctx_lh_Lat_Fis.ant.Vertical | ctx_lh_G_pariet_inf.Angular |
| ctx_lh_G_rectus | ctx_lh_G_and_S_paracentral |
| Right.Cerebellum.Cortex | ctx_rh_S_intrapariet_and_P_tran |
| Left.Putamen | Right.Accumbens.area |
| ctx_rh_G_subcallosal | ctx_rh_G_pariet_inf.Supramar |
| ctx_rh_G_and_S_subcentral | ctx_lh_G_insular_short |
| ctx_lh_G_insular_short | ctx_rh_S_subparietal |
| ctx_lh_S_orbital_med.olfact | ctx_lh_S_intrapariet_and_P_tran |
| ctx_lh_G_temp_sup.Lateral | ctx_rh_S_pericallosal |
| ctx_lh_G_Ins_lg_and_S_cent_ins | ctx_lh_G_front_middle |
| ctx_rh_S_collat_transv_post | ctx_lh_S_orbital_med.olfact |
| ctx_rh_S_central | ctx_rh_G_and_S_frontomargin |
| ctx_rh_S_parieto_occipital | ctx_rh_S_interm_prim.Jensen |
| ctx_lh_S_postcentral | Left.Putamen |
| ctx_rh_G_and_S_cingul.Mid.Post | ctx_lh_G_pariet_inf.Supramar |
| ctx_rh_G_cingul.Post.dorsal | ctx_lh_S_interm_prim.Jensen |
| ctx_lh_S_intrapariet_and_P_tran | ctx_rh_Lat_Fis.ant.Vertical |
| ctx_lh_S_precentral.inf.part | ctx_rh_G_subcallosal |
| ctx_lh_S_interm_prim.Jensen | ctx_lh_S_oc_middle_and_Lunatus |
| ctx_lh_G_and_S_paracentral | ctx_lh_S_precentral.inf.part |
| Right.Accumbens.area | ctx_rh_S_postcentral |
| ctx_rh_S_interm_prim.Jensen | ctx_rh_G_and_S_cingul.Mid.Post |
| ctx_lh_G_occipital_middle | ctx_rh_S_collat_transv_post |
| ctx_rh_S_oc_middle_and_Lunatus | ctx_lh_S_pericallosal |
| ctx_lh_G_and_S_cingul.Mid.Ant | ctx_rh_S_circular_insula_ant |
| ctx_lh_S_central | ctx_rh_S_central |
| ctx_rh_G_and_S_transv_frontopol | ctx_lh_G_cingul.Post.ventral |
| ctx_rh_S_circular_insula_ant | Left.Pallidum |
| ctx_rh_S_subparietal | ctx_lh_S_central |
| ctx_rh_G_temporal_inf | ctx_lh_G_cingul.Post.dorsal |
| ctx_rh_S_precentral.inf.part | ctx_rh_S_precentral.sup.part |
| ctx_lh_G_precentral | ctx_rh_G_oc.temp_med.Lingual |
| ctx_rh_G_orbital | ctx_rh_S_oc_middle_and_Lunatus |
| ctx_lh_G_postcentral | ctx_lh_G_and_S_cingul.Mid.Post |
| ctx_rh_G_pariet_inf.Angular | ctx_rh_S_parieto_occipital |
| ctx_rh_G_occipital_sup | ctx_lh_Pole_temporal |
| ctx_rh_G_front_middle | ctx_rh_G_Ins_lg_and_S_cent_ins |
| ctx_lh_G_occipital_sup | ctx_rh_S_collat_transv_ant |
| ctx_rh_S_collat_transv_ant | Right.Pallidum |
| ctx_lh_G_cingul.Post.dorsal | ctx_rh_S_orbital_lateral |
| ctx_rh_G_precuneus | ctx_rh_G_occipital_middle |
| ctx_rh_G_rectus | ctx_rh_G_occipital_sup |
| ctx_rh_G_occipital_middle | ctx_lh_G_precentral |
| ctx_rh_G_front_inf.Triangul | ctx_rh_G_oc.temp_lat.fusifor |
| ctx_lh_G_front_middle | ctx_lh_G_precuneus |
| ctx_rh_S_oc_sup_and_transversal | ctx_lh_Pole_occipital |
| ctx_rh_Lat_Fis.ant.Vertical | ctx_rh_G_cingul.Post.ventral |
| ctx_lh_G_cuneus | ctx_lh_G_occipital_sup |
| ctx_rh_G_and_S_paracentral | ctx_rh_G_and_S_paracentral |
| ctx_rh_G_insular_short | ctx_lh_G_cuneus |
| Left.Cerebellum.Cortex | ctx_rh_G_front_inf.Triangul |
| ctx_rh_G_temp_sup.Lateral | ctx_lh_S_temporal_transverse |
| ctx_lh_G_and_S_cingul.Mid.Post | ctx_rh_G_front_middle |
| ctx_rh_G_precentral | ctx_rh_G_pariet_inf.Angular |
| ctx_rh_G_oc.temp_lat.fusifor | ctx_lh_G_parietal_sup |
| ctx_rh_G_front_sup | ctx_lh_G_oc.temp_med.Lingual |
| ctx_rh_G_oc.temp_med.Lingual | ctx_rh_G_and_S_occipital_inf |
| ctx_lh_G_precuneus | ctx_rh_G_and_S_subcentral |
| ctx_rh_Pole_temporal | ctx_rh_G_orbital |
| ctx_lh_S_oc_sup_and_transversal | ctx_rh_G_precentral |
| ctx_lh_S_oc_middle_and_Lunatus | ctx_lh_S_collat_transv_post |
| ctx_lh_S_precentral.sup.part | Right.Cerebellum.Cortex |
| ctx_rh_S_orbital_lateral | ctx_rh_G_temp_sup.Lateral |
| ctx_lh_Pole_temporal | ctx_lh_G_postcentral |
| ctx_rh_G_postcentral | ctx_rh_G_postcentral |
| ctx_lh_G_parietal_sup | ctx_rh_S_precentral.inf.part |
| ctx_rh_G_front_inf.Orbital | ctx_rh_G_front_sup |
| ctx_rh_S_precentral.sup.part | ctx_lh_G_rectus |
| ctx_rh_G_parietal_sup | ctx_lh_S_oc_sup_and_transversal |
| ctx_lh_S_cingul.Marginalis | ctx_rh_G_insular_short |
| ctx_lh_G_oc.temp_med.Lingual | ctx_rh_G_precuneus |
| ctx_lh_S_pericallosal | ctx_rh_G_parietal_sup |
| ctx_rh_S_cingul.Marginalis | ctx_rh_S_cingul.Marginalis |
| ctx_lh_G_front_sup | ctx_lh_S_precentral.sup.part |
| ctx_rh_S_pericallosal | ctx_rh_S_oc_sup_and_transversal |
| ctx_rh_G_Ins_lg_and_S_cent_ins | ctx_rh_G_front_inf.Orbital |
| ctx_rh_G_cingul.Post.ventral | ctx_lh_S_oc.temp_med_and_Lingual |
| ctx_rh_Pole_occipital | ctx_lh_S_cingul.Marginalis |
| ctx_lh_Pole_occipital | Left.Cerebellum.Cortex |
| ctx_rh_G_and_S_occipital_inf | ctx_rh_Pole_occipital |
| ctx_lh_S_temporal_transverse | ctx_lh_G_front_sup |
| Right.Pallidum | ctx_rh_G_rectus |
| ctx_lh_S_collat_transv_post | ctx_rh_Pole_temporal |

**S1.21 Ranked features for both the EBM and FBM feature selection methods for the Hammers FA model.**

| **EBM** | **FBM** |
| --- | --- |
| Left Anterior Cinguli Gyrus | Left Anterior Cinguli Gyrus |
| Right Corpus Callosum | Left Corpus Callosum |
| Left Corpus Callosum | Right Corpus Callosum |
| Right Lateral Occipital Lobe | Right Lateral Temporal Ventricle |
| Right Posterior Temporal Lobe | Right Brainstem |
| Right Thalamus | Left Brainstem |
| Right Anterior Cinguli Gyrus | Right Caudate Nucleus |
| Left Middle Frontal Gyrus | Right Anterior Cinguli Gyrus |
| Left Brainstem | Left Lateral Temporal Ventricle |
| Right Middle Frontal Gyrus | Left Posterior Temporal Lobe |
| Right Lateral Temporal Ventricle | Right Middle Frontal Gyrus |
| Left Caudate Nucleus | Right Thalamus |
| Left Lateral Occipital Lobe | Right Superior Temporal Gyrus |
| Right Inferior Frontal Gyrus | Left Middle Frontal Gyrus |
| Right Caudate Nucleus | Right Inferior Middle Temporal Gyri |
| Left Ambient and Parahippocampus Gyri | Left Ambient and Parahippocampus Gyri |
| Left Posterior Temporal Lobe | Right Lateral Occipital Lobe |
| Left Lateral Temporal Ventricle | Left Caudate Nucleus |
| Right Brainstem | Right Posterior Temporal Lobe |
| Left Thalamus | Left Inferior Middle Temporal Gyri |
| Right Insula | Left Thalamus |
| Right Inferior Middle Temporal Gyri | Left Superior Frontal Gyrus |
| Left Hippocampus | Left Lateral Occipital Lobe |
| Left Putamen | Left Insula |
| Right Cerebellum | Right Third Ventricle |
| Left Inferior Middle Temporal Gyri | Left Superior Parietal Gyrus |
| Right Superior Parietal Gyrus | Left Third Ventricle |
| Left Orbito-Frontal Gyri | Left Inferior Lateral Pariatal Lobe |
| Left Accumbens Nucleus | Left Inferior Frontal Gyrus |
| Left Pallidum | Right Inferior Frontal Gyrus |
| Left Inferior Lateral Pariatal Lobe | Left Hippocampus |
| Left Insula | Left Pallidum |
| Right Inferior Lateral Pariatal Lobe | Right Hippocampus |
| Right Fusiform Gyrus | Left Fusiform Gyrus |
| Right Superior Temporal Gyrus | Left Orbito-Frontal Gyri |
| Right Lingual Gyrus | Right Pallidum |
| Left Anterior Medial Temporal Lobe | Right Ambient and Parahippocampus Gyri |
| Left Superior Frontal Gyrus | Right Superior Parietal Gyrus |
| Left Precentral Gyrus | Right Insula |
| Right Ambient and Parahippocampus Gyri | Right Fusiform Gyrus |
| Left Fusiform Gyrus | Left Superior Temporal Gyrus |
| Right Anterior Lateral Temporal Lobe | Left Cerebellum |
| Right Putamen | Right Cerebellum |
| Left Cuneus | Left Lingual Gyrus |
| Left Inferior Frontal Gyrus | Right Putamen |
| Right Third Ventricle | Right Cuneus |
| Right Gyrus Rectus | Right Precentral Gyrus |
| Right Hippocampus | Left Cuneus |
| Left Superior Temporal Gyrus | Right Superior Frontal Gyrus |
| Left Anterior Lateral Temporal Lobe | Left Anterior Lateral Temporal Lobe |
| Left Superior Parietal Gyrus | Left Amygdala |
| Right Cuneus | Right Inferior Lateral Pariatal Lobe |
| Left Amygdala | Left Posterior Cinguli Gyrus |
| Left Cerebellum | Left Gyrus Rectus |
| Right Postcentral Gyrus | Left Accumbens Nucleus |
| Left Third Ventricle | Right Amygdala |
| Right Superior Frontal Gyrus | Left Precentral Gyrus |
| Right Pallidum | Right Anterior Medial Temporal Lobe |
| Right Posterior Cinguli Gyrus | Right Anterior Lateral Temporal Lobe |
| Right Precentral Gyrus | Right Posterior Cinguli Gyrus |
| Left Lingual Gyrus | Right Lingual Gyrus |
| Left Posterior Cinguli Gyrus | Left Postcentral Gyrus |
| Right Orbito-Frontal Gyri | Right Gyrus Rectus |
| Right Anterior Medial Temporal Lobe | Left Anterior Medial Temporal Lobe |
| Right Amygdala | Right Postcentral Gyrus |
| Left Gyrus Rectus | Right Orbito-Frontal Gyri |
| Left Postcentral Gyrus | Right Accumbens Nucleus |
| Right Accumbens Nucleus | Left Putamen |

**S1.22 Ranked features for both the EBM and FBM feature selection methods for the Hammers MD model.**

| **EBM** | **FBM** |
| --- | --- |
| Left Inferior Frontal Gyrus | Left Superior Temporal Gyrus |
| Right Posterior Temporal Lobe | Left Fusiform Gyrus |
| Left Amygdala | Left Inferior Middle Temporal Gyri |
| Right Putamen | Left Inferior Frontal Gyrus |
| Left Fusiform Gyrus | Left Posterior Temporal Lobe |
| Left Superior Temporal Gyrus | Left Anterior Cinguli Gyrus |
| Left Inferior Middle Temporal Gyri | Right Anterior Cinguli Gyrus |
| Left Ambient and Parahippocampus Gyri | Left Amygdala |
| Left Anterior Cinguli Gyrus | Right Hippocampus |
| Left Posterior Temporal Lobe | Right Posterior Temporal Lobe |
| Left Orbito-Frontal Gyri | Left Inferior Lateral Pariatal Lobe |
| Right Superior Temporal Gyrus | Left Hippocampus |
| Left Putamen | Right Inferior Middle Temporal Gyri |
| Right Gyrus Rectus | Right Putamen |
| Right Fusiform Gyrus | Left Anterior Lateral Temporal Lobe |
| Right Anterior Cinguli Gyrus | Right Fusiform Gyrus |
| Left Hippocampus | Left Orbito-Frontal Gyri |
| Left Insula | Right Superior Temporal Gyrus |
| Left Anterior Lateral Temporal Lobe | Left Insula |
| Left Caudate Nucleus | Right Gyrus Rectus |
| Right Hippocampus | Left Middle Frontal Gyrus |
| Left Cerebellum | Left Ambient and Parahippocampus Gyri |
| Right Thalamus | Left Accumbens Nucleus |
| Right Inferior Frontal Gyrus | Right Thalamus |
| Right Inferior Middle Temporal Gyri | Left Lateral Occipital Lobe |
| Left Inferior Lateral Pariatal Lobe | Left Gyrus Rectus |
| Left Middle Frontal Gyrus | Right Amygdala |
| Left Gyrus Rectus | Left Putamen |
| Right Corpus Callosum | Right Inferior Lateral Pariatal Lobe |
| Left Accumbens Nucleus | Right Lateral Temporal Ventricle |
| Right Posterior Cinguli Gyrus | Right Middle Frontal Gyrus |
| Right Amygdala | Right Caudate Nucleus |
| Right Middle Frontal Gyrus | Left Caudate Nucleus |
| Right Inferior Lateral Pariatal Lobe | Right Inferior Frontal Gyrus |
| Left Lateral Occipital Lobe | Right Pallidum |
| Right Third Ventricle | Left Posterior Cinguli Gyrus |
| Right Cuneus | Right Lateral Occipital Lobe |
| Right Caudate Nucleus | Right Insula |
| Left Cuneus | Right Posterior Cinguli Gyrus |
| Right Pallidum | Right Orbito-Frontal Gyri |
| Right Lateral Temporal Ventricle | Right Cuneus |
| Right Anterior Lateral Temporal Lobe | Right Accumbens Nucleus |
| Left Posterior Cinguli Gyrus | Right Corpus Callosum |
| Left Precentral Gyrus | Left Thalamus |
| Right Orbito-Frontal Gyri | Left Superior Frontal Gyrus |
| Right Accumbens Nucleus | Right Anterior Lateral Temporal Lobe |
| Right Precentral Gyrus | Left Corpus Callosum |
| Left Anterior Medial Temporal Lobe | Left Pallidum |
| Right Cerebellum | Right Lingual Gyrus |
| Left Superior Parietal Gyrus | Right Superior Frontal Gyrus |
| Right Insula | Right Ambient and Parahippocampus Gyri |
| Left Corpus Callosum | Left Superior Parietal Gyrus |
| Right Lingual Gyrus | Left Lateral Temporal Ventricle |
| Right Lateral Occipital Lobe | Right Superior Parietal Gyrus |
| Right Ambient and Parahippocampus Gyri | Left Postcentral Gyrus |
| Right Anterior Medial Temporal Lobe | Left Cuneus |
| Left Postcentral Gyrus | Right Anterior Medial Temporal Lobe |
| Left Superior Frontal Gyrus | Left Anterior Medial Temporal Lobe |
| Right Superior Parietal Gyrus | Left Third Ventricle |
| Right Postcentral Gyrus | Left Lingual Gyrus |
| Right Superior Frontal Gyrus | Right Third Ventricle |
| Left Pallidum | Left Precentral Gyrus |
| Left Lingual Gyrus | Right Precentral Gyrus |
| Left Lateral Temporal Ventricle | Right Cerebellum |
| Left Third Ventricle | Left Brainstem |
| Right Brainstem | Right Brainstem |
| Left Thalamus | Right Postcentral Gyrus |
| Left Brainstem | Left Cerebellum |

**S1.23 Ranked features for both the EBM and FBM feature selection methods for the JHU FA model.**

| **EBM** | **FBM** |
| --- | --- |
| Superior fronto occipital fasciculus Left | Anterior corona radiata Right |
| Anterior corona radiata Right | Superior fronto occipital fasciculus Left |
| Medial lemniscus Left | Genu of corpus callosum |
| Splenium of corpus callosum | Splenium of corpus callosum |
| Fornix Stria terminalis Right | Fornix |
| Genu of corpus callosum | Medial lemniscus Left |
| Fornix | Superior cerebellar peduncle Left |
| Cingulum hippocampus Right | Fornix Stria terminalis Right |
| Anterior corona radiata Left | Anterior corona radiata Left |
| Superior cerebellar peduncle Left | Cingulum hippocampus Right |
| Body of corpus callosum | Body of corpus callosum |
| Posterior thalamic radiation Left | Posterior thalamic radiation Left |
| Cingulum cingulate gyrus Left | Fornix Stria terminalis Left |
| Anterior limb of internal capsule Left | Cingulum cingulate gyrus Right |
| Inferior cerebellar peduncle Left | Inferior cerebellar peduncle Left |
| Cingulum cingulate gyrus Right | Rightetrolenticular part of internal capsule Left |
| Superior cerebellar peduncle Right | Superior cerebellar peduncle Right |
| Pontine crossing tract | Pontine crossing tract |
| Fornix Stria terminalis Left | Posterior thalamic radiation Right |
| Middle cerebellar peduncle | Medial lemniscus Right |
| Anterior limb of internal capsule Right | Inferior cerebellar peduncle Right |
| Posterior thalamic radiation Right | Anterior limb of internal capsule Left |
| Superior corona radiata Left | Corticospinal tract Left |
| Medial lemniscus Right | Cerebral peduncle Right |
| Rightetrolenticular part of internal capsule Left | Posterior corona radiata Left |
| External capsule Right | Cerebral peduncle Left |
| Corticospinal tract Left | Cingulum cingulate gyrus Left |
| Uncinate fasciculus Right | Tapetum Left |
| Superior corona radiata Right | Cingulum hippocampus Left |
| Posterior limb of internal capsule Left | Uncinate fasciculus Right |
| External capsule Left | Sagittal stratum Left |
| Posterior corona radiata Left | Anterior limb of internal capsule Right |
| Inferior cerebellar peduncle Right | Rightetrolenticular part of internal capsule Right |
| Tapetum Left | Middle cerebellar peduncle |
| Rightetrolenticular part of internal capsule Right | Tapetum Right |
| Cerebral peduncle Left | Corticospinal tract Right |
| Uncinate fasciculus Left | Posterior corona radiata Right |
| Sagittal stratum Left | Superior corona radiata Right |
| Posterior limb of internal capsule Right | Posterior limb of internal capsule Right |
| Cingulum hippocampus Left | External capsule Right |
| Superior longitudinal fasciculus Left | External capsule Left |
| Superior fronto occipital fasciculus Right | Superior corona radiata Left |
| Corticospinal tract Right | Posterior limb of internal capsule Left |
| Cerebral peduncle Right | Sagittal stratum Right |
| Posterior corona radiata Right | Uncinate fasciculus Left |
| Superior longitudinal fasciculus Right | Superior fronto occipital fasciculus Right |
| Sagittal stratum Right | Superior longitudinal fasciculus Right |
| Tapetum Right | Superior longitudinal fasciculus Left |

**S1.24 Ranked features for both the EBM and FBM feature selection methods for the JHU MD model.**

| **EBM** | **FBM** |
| --- | --- |
| Tapetum Right | Genu of corpus callosum |
| Cingulum cingulate gyrus Right | Cingulum hippocampus Right |
| Cingulum hippocampus Right | Fornix Stria terminalis Right |
| Uncinate fasciculus Right | Medial lemniscus Left |
| Superior fronto occipital fasciculus Left | Cingulum cingulate gyrus Right |
| Anterior corona radiata Right | Anterior corona radiata Right |
| Posterior thalamic radiation Right | Posterior thalamic radiation Right |
| Fornix Stria terminalis Right | Fornix Stria terminalis Left |
| Medial lemniscus Left | Tapetum Right |
| External capsule Left | Uncinate fasciculus Right |
| Fornix Stria terminalis Left | Posterior corona radiata Left |
| Genu of corpus callosum | Corticospinal tract Right |
| Anterior limb of internal capsule Left | Superior corona radiata Left |
| Anterior corona radiata Left | Cingulum cingulate gyrus Left |
| Inferior cerebellar peduncle Left | Splenium of corpus callosum |
| Posterior limb of internal capsule Left | Anterior limb of internal capsule Right |
| Uncinate fasciculus Left | Rightetrolenticular part of internal capsule Right |
| Splenium of corpus callosum | Superior fronto occipital fasciculus Left |
| Medial lemniscus Right | Rightetrolenticular part of internal capsule Left |
| Rightetrolenticular part of internal capsule Right | Anterior corona radiata Left |
| Superior corona radiata Left | External capsule Right |
| Corticospinal tract Left | Superior longitudinal fasciculus Right |
| Posterior thalamic radiation Left | Middle cerebellar peduncle |
| Middle cerebellar peduncle | Fornix |
| Superior longitudinal fasciculus Right | Medial lemniscus Right |
| Cingulum cingulate gyrus Left | Corticospinal tract Left |
| Rightetrolenticular part of internal capsule Left | Superior corona radiata Right |
| Posterior corona radiata Left | Superior fronto occipital fasciculus Right |
| Fornix | Sagittal stratum Right |
| Posterior corona radiata Right | Posterior corona radiata Right |
| Sagittal stratum Right | Sagittal stratum Left |
| Sagittal stratum Left | Posterior thalamic radiation Left |
| Superior corona radiata Right | Pontine crossing tract |
| Superior fronto occipital fasciculus Right | Superior longitudinal fasciculus Left |
| External capsule Right | Anterior limb of internal capsule Left |
| Superior cerebellar peduncle Left | Posterior limb of internal capsule Left |
| Anterior limb of internal capsule Right | Inferior cerebellar peduncle Left |
| Corticospinal tract Right | Tapetum Left |
| Body of corpus callosum | Body of corpus callosum |
| Cerebral peduncle Right | Cerebral peduncle Left |
| Superior longitudinal fasciculus Left | Uncinate fasciculus Left |
| Tapetum Left | External capsule Left |
| Pontine crossing tract | Cerebral peduncle Right |
| Inferior cerebellar peduncle Right | Posterior limb of internal capsule Right |
| Cingulum hippocampus Left | Superior cerebellar peduncle Left |
| Superior cerebellar peduncle Right | Superior cerebellar peduncle Right |
| Cerebral peduncle Left | Inferior cerebellar peduncle Right |
| Posterior limb of internal capsule Right | Cingulum hippocampus Left |

**S1.25 Ranked features for both the EBM and FBM feature selection methods for the SUVR Cerebellum model.**

| **EBM** | **FBM** |
| --- | --- |
| Precuneus | Parietal Superior |
| Parietal Inferior | Parietal Inferior |
| Prefrontal Cortex | Precuneus |
| Parietal Superior | Occipital Cortex |
| Anterior Cingulate | Prefrontal Cortex |
| Occipital Cortex | Anterior Cingulate |
| Putamen Anterior Left | Putamen Anterior Left |
| Putamen Anterior Right | Putamen Anterior Right |
| Putamen Entire Right | Putamen Entire Left |
| Putamen Middle Right | Putamen Middle Left |
| Putamen Middle Left | Putamen Entire Right |
| Putamen Entire Left | Putamen |
| Putamen | Putamen Middle Right |
| Pons | Posterior Cingulate |
| Putamen Posterior Left | Putamen Posterior Right |
| Posterior Cingulate | Putamen Posterior Left |
| Putamen Posterior Right | Pons |
| Caudate | Mesial Temporal Cortex |
| Mesial Temporal Cortex | Caudate |

**S1.26 Ranked features for both the EBM and FBM feature selection methods for the SUVR GM model.**

| **EBM** | **FBM** |
| --- | --- |
| Pons | Pons |
| Mesial Temporal Cortex | Mesial Temporal Cortex |
| Prefrontal Cortex | Parietal Inferior |
| Parietal Inferior | Parietal Superior |
| Parietal Superior | Prefrontal Cortex |
| Precuneus | Precuneus |
| Caudate | Putamen Posterior Left |
| Putamen Posterior Right | Putamen Posterior Right |
| Anterior Cingulate | Caudate |
| Putamen Posterior Left | Anterior Cingulate |
| Posterior Cingulate | Posterior Cingulate |
| Putamen Anterior Left | Putamen Anterior Left |
| Putamen Anterior Right | Putamen Middle Left |
| Putamen Middle Left | Putamen |
| Occipital Cortex | Putamen Anterior Right |
| Putamen Middle Right | Occipital Cortex |
| Putamen Entire Left | Putamen Middle Right |
| Putamen Entire Right | Putamen Entire Right |
| Putamen | Putamen Entire Left |

**S1.27 Ranked features for both the EBM and FBM feature selection methods for the SUVR WM model.**

| **EBM** | **FBM** |
| --- | --- |
| Prefrontal Cortex | Anterior Cingulate |
| Anterior Cingulate | Precuneus |
| Pons | Pons |
| Parietal Inferior | Putamen Anterior Left |
| Precuneus | Parietal Inferior |
| Putamen Anterior Right | Parietal Superior |
| Putamen Anterior Left | Putamen Anterior Right |
| Parietal Superior | Prefrontal Cortex |
| Putamen Entire Left | Putamen Entire Left |
| Putamen | Putamen |
| Putamen Middle Left | Occipital Cortex |
| Mesial Temporal Cortex | Putamen Middle Left |
| Putamen Entire Right | Putamen Entire Right |
| Putamen Middle Right | Mesial Temporal Cortex |
| Occipital Cortex | Putamen Middle Right |
| Caudate | Caudate |
| Posterior Cingulate | Posterior Cingulate |
| Putamen Posterior Right | Putamen Posterior Left |
| Putamen Posterior Left | Putamen Posterior Right |
