# Supplementary material for: Combined Structural MR and Diffusion Tensor Imaging Classify the Presence of Alzheimer’s Disease With the Same Performance as MR Combined With Amyloid Positron Emission Tomography: A Data Integration Approach
Source: Front Neurosci. 2022 Jan 5;15:638175. doi: 10.3389/fnins.2021.638175 (PMC8766722; doi:10.3389/fnins.2021.638175)
Supplement: Supplementary file 4 [file Table_2.docx]

| Imaging Modality | Atlas | Accuracy | Sensitivity | Specificity | AUC |
| --- | --- | --- | --- | --- | --- |
| MRI Structural | Cobra GM | 76.13% | 76.06% | 76.19% | 0.87±0.13 |
|  | Cobra WM | 78.43% | 79.04% | 77.04% | 0.88±0.12 |
|  | Hammers GM | 74.79% | 66.14% | 83.44% | 0.85±0.15 |
|  | Hammers WM | 58.36% | 58.24% | 58.49% | 0.51±0.24 |
|  | Hammers CSF | 75.37% | 77.01% | 73.73% | 0.85±0.14 |
|  | Lpba40 GM | 70.28% | 61.16% | 79.40% | 0.76±0.20 |
|  | Neuromorphometrics GM | 92.05% | 86.78% | 97.32% | 0.96±0.04 |
|  | Neuromorphometrics CSF | 76.13% | 80.34% | 71.91% | 0.84±0.15 |
| MRI Surface | a2009 Gyrification | 83.01% | 86.98% | 79.04% | 0.91±0.10 |
|  | a2009 Thickness | 81.89% | 81.93% | 81.87% | 0.90±0.11 |
|  | Dk40 Gyrification | 69.13% | 73.14% | 65.11% | 0.75±0.19 |
|  | Dk40 Thickness | 72.19% | 71.01% | 73.36% | 0.79±0.17 |
|  | HCP Gyrification | 83.11% | 73.65% | 92.56% | 0.94±0.08 |
|  | HCP Thickness | 84.27% | 83.05% | 85.49% | 0.93±0.09 |
| DTI | Lpba40 FA | 71.34% | 65.37% | 75.81% | 0.72±0.24 |
|  | Lpba40 MD | 75.29% | 67.32% | 81.04% | 0.86±0.15 |
|  | Desikan FA | 79.84% | 76.67% | 82.23% | 0.86±0.15 |
|  | Desikan MD | 62.00% | 52.70% | 68.98% | 0.66±0.24 |
|  | Destrieux FA | 65.66% | 63.23% | 67.49% | 0.66±0.25 |
|  | Destrieux MD | 77.60% | 72.15% | 81.69% | 0.84±0.15 |
|  | Hammers FA | 76.77% | 63.80% | 86.50% | 0.79±0.19 |
|  | Hammers MD | 69.52% | 55.63% | 79.94% | 0.72±0.22 |
|  | JHU FA | 77.24% | 67.15% | 84.81% | 0.80±0.19 |
|  | JHU MD | 63.86% | 47.47% | 76.16% | 0.61±0.26 |
| PiB-PET | SUVR Cerebellum | 87.71% | 95.92% | 81.26% | 0.94±0.09 |
|  | SUVR GM | 91.98% | 98.66% | 87.01% | 0.97±0.06 |
|  | SUVR WM | 90.68% | 92.78% | 89.10% | 0.93±0.10 |

**Table S2.1. Classifier’s performance using embedded-based feature selection and the RBF kernel for all atlases.**

**Table S2.2. Classifier’s performance using embedded-based feature selection and the linear kernel for all atlases.**

| Imaging Modality | Atlas | Accuracy | Sensitivity | Specificity | AUC |
| --- | --- | --- | --- | --- | --- |
| MRI Structural | Cobra GM | 74.31% | 70.78% | 77.84% | 0.83±0.15 |
|  | Cobra WM | 79.19% | 75.29% | 83.10% | 0.88±0.11 |
|  | Hammers GM | 74.71% | 69.81% | 79.61% | 0.84±0.15 |
|  | Hammers WM | 61.15% | 55.02% | 67.27% | 0.62±0.22 |
|  | Hammers CSF | 73.08% | 65.94% | 80.23% | 0.84±0.16 |
|  | Lpba40 GM | 67.51% | 62.06% | 72.96% | 0.76±0.18 |
|  | Neuromorphometrics GM | 82.36% | 75.49% | 89.24% | 0.90±0.11 |
|  | Neuromorphometrics CSF | 75.38% | 74.46% | 76.29% | 0.84±0.15 |
| MRI Surface | a2009 Gyrification | 83.16% | 83.41% | 82.91% | 0.89±0.12 |
|  | a2009 Thickness | 82.16% | 82.49% | 81.83% | 0.91±0.11 |
|  | Dk40 Gyrification | 66.81% | 68.71% | 64.90% | 0.73±0.18 |
|  | Dk40 Thickness | 69.51% | 67.89% | 71.12% | 0.81±0.15 |
|  | HCP Gyrification | 85.08% | 83.31% | 86.84% | 0.93±0.09 |
|  | HCP Thickness | 83.83% | 79.97% | 87.69% | 0.90±0.12 |
| DTI | Lpba40 FA | 70.74% | 60.75% | 78.24% | 0.75±0.21 |
|  | Lpba40 MD | 72.48% | 64.18% | 78.70% | 0.80±0.18 |
|  | Desikan FA | 76.51% | 72.17% | 79.76% | 0.80±0.19 |
|  | Desikan MD | 65.04% | 55.73% | 72.01% | 0.68±0.22 |
|  | Destrieux FA | 57.14% | 53.32% | 60.00% | 0.51±0.24 |
|  | Destrieux MD | 73.43% | 66.33% | 78.75% | 0.79±0.18 |
|  | Hammers FA | 69.65% | 63.15% | 74.52% | 0.60±0.28 |
|  | Hammers MD | 63.86% | 48.00% | 75.76% | 0.56±0.24 |
|  | JHU FA | 75.23% | 67.50% | 81.03% | 0.78±0.20 |
|  | JHU MD | 64.01% | 60.20% | 66.88% | 0.67±0.23 |
| PiB-PET | SUVR Cerebellum | 89.38% | 100.00% | 81.41% | 0.96±0.07 |
|  | SUVR GM | 90.47% | 98.23% | 84.65% | 0.96±0.08 |
|  | SUVR WM | 90.64% | 91.50% | 90.00% | 0.95±0.08 |
