# Supplementary material for: Combined Structural MR and Diffusion Tensor Imaging Classify the Presence of Alzheimer’s Disease With the Same Performance as MR Combined With Amyloid Positron Emission Tomography: A Data Integration Approach
Source: Front Neurosci. 2022 Jan 5;15:638175. doi: 10.3389/fnins.2021.638175 (PMC8766722; doi:10.3389/fnins.2021.638175)
Supplement: Supplementary file 5 [file Table_3.docx]

| Imaging Modality | Atlas | Accuracy | Sensitivity | Specificity | AUC |
| --- | --- | --- | --- | --- | --- |
| MRI Structural | Cobra GM | 82.08% | 73.63% | 90.54% | 0.87±0.13 |
|  | Cobra WM | 77.06% | 77.69% | 76.44% | 0.86±0.13 |
|  | Hammers GM | 72.92% | 67.26% | 78.57% | 0.77±0.20 |
|  | Hammers WM | 58.27% | 56.57% | 59.96% | 0.50±0.23 |
|  | Hammers CSF | 71.65% | 72.24% | 71.06% | 0.79±0.17 |
|  | Lpba40 GM | 73.93% | 67.23% | 80.64% | 0.78±0.17 |
|  | Neuromorphometrics GM | 86.02% | 82.70% | 89.34% | 0.93±0.09 |
|  | Neuromorphometrics CSF | 60.94% | 64.78% | 57.10% | 0.61±0.23 |
| MRI Surface | a2009 Gyrification | 76.45% | 77.70% | 75.20% | 0.86±0.13 |
|  | a2009 Thickness | 81.47% | 82.76% | 80.18% | 0.84±0.15 |
|  | Dk40 Gyrification | 69.57% | 73.99% | 65.15% | 0.78±0.18 |
|  | Dk40 Thickness | 75.07% | 74.86% | 75.28% | 0.82±0.15 |
|  | HCP Gyrification | 85.59% | 79.51% | 91.68% | 0.96±0.07 |
|  | HCP Thickness | 88.07% | 84.59% | 91.55% | 0.93±0.09 |
| DTI | Lpba40 FA | 69.26% | 53.77% | 80.88% | 0.67±0.26 |
|  | Lpba40 MD | 73.76% | 67.78% | 78.25% | 0.75±0.20 |
|  | Desikan FA | 79.68% | 72.48% | 85.08% | 0.86±0.14 |
|  | Desikan MD | 76.66% | 63.72% | 86.38% | 0.85±0.15 |
|  | Destrieux FA | 66.31% | 55.63% | 74.31% | 0.66±0.24 |
|  | Destrieux MD | 88.14% | 81.68% | 92.68% | 0.93±0.10 |
|  | Hammers FA | 74.12% | 57.68% | 86.45% | 0.72±0.23 |
|  | Hammers MD | 70.83% | 53.67% | 83.70% | 0.73±0.22 |
|  | JHU FA | 79.35% | 71.05% | 85.58% | 0.83±0.17 |
|  | JHU MD | 69.22% | 53.53% | 80.99% | 0.68±0.25 |
| PiB-PET | SUVR Cerebellum | 88.06% | 96.67% | 81.60% | 0.95±0.07 |
|  | SUVR GM | 87.15% | 95.73% | 80.71% | 0.97±0.06 |
|  | SUVR WM | 93.52% | 99.72% | 88.90% | 0.94±0.10 |

Table S3.1 and S3.2 show the results for the models built with the features obtained from the filter-based methods (FBM) and using a SVM model built with an RBF and linear kernel, respectively. The hyperparameters gamma and C of both SVM models were set to “scale” and 0.1, respectively. The SVM models were constructed using the scikit-learning package on a python environment.

**Table S3.1. Classifier’s performance using filter-based feature selection and using the SVM classifier with an RBF kernel for all atlases.**

| Imaging Modality | Atlas | Accuracy | Sensitivity | Specificity | AUC |
| --- | --- | --- | --- | --- | --- |
| MRI Structural | Cobra GM | 71.19% | 67.58% | 74.81% | 0.81±0.15 |
|  | Cobra WM | 80.61% | 77.06% | 84.16% | 0.91±0.10 |
|  | Hammers GM | 69.49% | 66.09% | 72.89% | 0.81±0.15 |
|  | Hammers WM | 62.76% | 52.48% | 73.05% | 0.66±0.25 |
|  | Hammers CSF | 71.41% | 69.03% | 73.80% | 0.85±0.13 |
|  | Lpba40 GM | 73.13% | 72.62% | 73.64% | 0.83±0.14 |
|  | Neuromorphometrics GM | 84.98% | 81.30% | 88.66% | 0.94±0.09 |
|  | Neuromorphometrics CSF | 66.56% | 66.35% | 66.77% | 0.76±0.18 |
| MRI Surface | a2009 Gyrification | 81.61% | 80.85% | 82.36% | 0.89±0.12 |
|  | a2009 Thickness | 76.56% | 75.49% | 77.62% | 0.83±0.15 |
|  | Dk40 Gyrification | 67.31% | 68.39% | 66.24% | 0.76±0.17 |
|  | Dk40 Thickness | 70.81% | 69.76% | 71.86% | 0.78±0.17 |
|  | HCP Gyrification | 84.56% | 84.19% | 84.94% | 0.94±0.08 |
|  | HCP Thickness | 86.91% | 83.11% | 90.70% | 0.93±0.10 |
| DTI | Lpba40 FA | 72.48% | 66.68% | 76.83% | 0.71±0.25 |
|  | Lpba40 MD | 75.85% | 69.00% | 80.99% | 0.82±0.16 |
|  | Desikan FA | 77.46% | 74.20% | 79.91% | 0.83±0.16 |
|  | Desikan MD | 67.13% | 57.47% | 74.38% | 0.75±0.19 |
|  | Destrieux FA | 68.84% | 64.33% | 72.21% | 0.69±0.24 |
|  | Destrieux MD | 85.51% | 80.12% | 89.56% | 0.91±0.13 |
|  | Hammers FA | 77.05% | 69.98% | 82.35% | 0.77±0.21 |
|  | Hammers MD | 66.11% | 47.78% | 79.86% | 0.61±0.24 |
|  | JHU FA | 75.41% | 71.98% | 77.99% | 0.81±0.18 |
|  | JHU MD | 67.06% | 58.32% | 73.61% | 0.71±0.22 |
| PiB-PET | SUVR Cerebellum | 88.90% | 99.57% | 80.90% | 0.98±0.05 |
|  | SUVR GM | 87.91% | 93.07% | 84.04% | 0.96±0.08 |
|  | SUVR WM | 90.36% | 91.88% | 89.22% | 0.96±0.07 |

**Table S3.2. Classifier’s performance using filter-based feature selection and using the SVM classifier with a linear kernel for all atlases.**
